# Cell–cell communication: new insights and clinical implications

**DOI:** 10.1038/s41392-024-01888-z

**Published:** 2024-08-07

**Authors:** Jimeng Su, Ying Song, Zhipeng Zhu, Xinyue Huang, Jibiao Fan, Jie Qiao, Fengbiao Mao

**Affiliations:** 1 Institute of Medical Innovation and Research, Peking University Third Hospital, Beijing, China; 2 Cancer Center, Peking University Third Hospital, Beijing, China; 3 College of Animal Science and Technology, Yangzhou University, Yangzhou, Jiangsu China; 4 Biomedical Research Institute, Shenzhen Peking University-the Hong Kong University of Science and Technology Medical Center, Shenzhen, China; 5 State Key Laboratory of Female Fertility Promotion, Department of Obstetrics and Gynecology, Peking University Third Hospital, Beijing, China; 6 National Clinical Research Center for Obstetrics and Gynecology (Peking University Third Hospital), Beijing, China; 7 Key Laboratory of Assisted Reproduction (Peking University), Ministry of Education, Beijing, China; 8 Beijing Key Laboratory of Reproductive Endocrinology and Assisted Reproductive Technology, Beijing, China

**Keywords:** Gene expression analysis, Genome informatics, Epigenetics

## Abstract

Multicellular organisms are composed of diverse cell types that must coordinate their behaviors through communication. Cell–cell communication (CCC) is essential for growth, development, differentiation, tissue and organ formation, maintenance, and physiological regulation. Cells communicate through direct contact or at a distance using ligand–receptor interactions. So cellular communication encompasses two essential processes: cell signal conduction for generation and intercellular transmission of signals, and cell signal transduction for reception and procession of signals. Deciphering intercellular communication networks is critical for understanding cell differentiation, development, and metabolism. First, we comprehensively review the historical milestones in CCC studies, followed by a detailed description of the mechanisms of signal molecule transmission and the importance of the main signaling pathways they mediate in maintaining biological functions. Then we systematically introduce a series of human diseases caused by abnormalities in cell communication and their progress in clinical applications. Finally, we summarize various methods for monitoring cell interactions, including cell imaging, proximity-based chemical labeling, mechanical force analysis, downstream analysis strategies, and single-cell technologies. These methods aim to illustrate how biological functions depend on these interactions and the complexity of their regulatory signaling pathways to regulate crucial physiological processes, including tissue homeostasis, cell development, and immune responses in diseases. In addition, this review enhances our understanding of the biological processes that occur after cell–cell binding, highlighting its application in discovering new therapeutic targets and biomarkers related to precision medicine. This collective understanding provides a foundation for developing new targeted drugs and personalized treatments.

## Introduction

The coordination of cellular activities, essential for multicellular existence, is contingent upon cell–cell interactions (CCIs) among a variety of cell types and tissues throughout an organism.^1–3^ Cell–cell communication (CCC) is an essential process that profoundly influences an organism’s homeostasis, development, and disease processes.^4^ Typically, CCC involves interactions with secreted ligands and plasma membrane receptors, yet it also includes secretases, extracellular matrix proteins, transporters, and direct cell-to-cell contact mechanisms.^5^ Different cells employ different CCCs to ensure biological development, homeostasis, and tissue repair.

Essentially, CCC is a fundamental characteristic of multicellular organisms.^6^ The dynamic communication network established between cells through collaboration plays a pivotal role in various biological processes.^6–8^ This interaction is integral to the functioning of living organisms, influencing cellular metabolism, energy transformation, maintenance of physiological functions, regulation of growth, development, immune responses, single-cell functions, and other critical life processes.^9^ For example, during immune responses, CCCs enable immune cells to recognize and combat pathogens. In growth and development, CCCs regulate cell proliferation and differentiation, facilitating the normal development of organs and tissues. Diseases typically manifest when cells fail to interact correctly or misinterpret molecular information.^5^

CCCs reflect the fundamental level of physiological communication, triggering responses to internal or external environments essential for survival. When cells communicate with each other, extracellular signals typically induce intracellular signal transduction cascades, leading to cellular responses such as changes in the cytoskeleton, metabolism, or gene expression.^10^ The regulation and feedback mechanisms at various levels of these transduction cascades modulate the pathway’s activity over time.^11^ Signal pathways are the basis of internal communication and response to the external environment in organisms. They are responsible for converting extracellular signals into intracellular responses, thereby regulating cell behavior and function. These pathways involve a series of precise molecular events, including the reception of signals, amplification, distribution, and the triggering of specific cellular responses.^12,13^ Critical cellular determinations, such as cytoskeletal reorganization, cell cycle checkpoints, and programmed cell death, are contingent upon the stringent temporal regulation and the specific spatial distribution of activated signal transducers.^14^ Understanding how these pathways are disrupted in diseases offers the possibility for developing new therapeutic approaches.^15–17^

The complexity of CCC has been recognized as part of the molecular mechanisms of developmental biology, carcinogenesis, and organ dysfunction.^18^ Exploring CCC dynamic changes under different conditions provides deeper insights into the underlying mechanisms of diverse biological processes and helps elucidate the mechanisms behind the onset and progression of diseases. Over the decade, single-cell RNA sequencing has gained widespread use across multiple research fields to investigate the critical role of ligand–receptor dynamics in intercellular communication.^19^ Technologies like single-cell RNA sequencing (scRNA-Seq) empower researchers to explore the intricate communication patterns between different cell types within multicellular organisms, offering fresh perspectives on cell communication mechanisms, cell functions, and the organization of cell populations. The analysis of intercellular communication assists in understanding the interplay between cells, dissecting communication networks, uncovering various cell interactions in the developmental process, exploring the tumor immune microenvironment, and identifying potential therapeutic targets for diseases.^20^ Therefore, identifying and quantifying intercellular signaling pathways have become standard practices across diverse disciplines.

Activating specific cell signaling pathways through ligand–receptor interactions (LRIs) constitutes a fundamental mode of cell communication and is intricately linked to various degenerative processes and diseases. Different cell types share common biological elements facilitating these interactions, encompassing ligands, surface receptors, adhesion proteins, intracellular adaptors, as well as glycans, lipids, cytoskeletons, and scaffolding proteins.^21^ Comprehending the orchestration of biophysical, genetic, and biochemical events in CCCs by these shared components across various cell types is crucial for developing clinical therapies based on proteins and cells that either modulate or utilize intercellular communication.^22,23^ The analysis of LRIs provides the foundation for comprehending cell behavior and responses to neighboring cells.^24^

Historically, CCC research has primarily been confined to experiments conducted in vitro involving one or two types of cells and a limited set of genes. With advancements in science and technology, data acquisition at the single-cell level enables the detection of low-abundance genes and provides a robust foundation for cell communication study. In recent years, multiple research efforts have concentrated on intercellular signaling by employing either the co-expression of all genes or particular cell markers,^25,26^ the resemblance in expression patterns.^27^ or the characteristics of regulatory networks.^28^ Understanding LRIs is an effective approach to understand cellular communication at the single-cell level, and a multitude of research endeavors are dedicated to formulating strategies to construct cellular communication networks based on these interactions. Harnessing these technologies, many laboratories have developed various algorithms and softwares for cell communication research.

The review comprehensively outlines the experimental and computational CCC methods rooted in chemistry and biology to decode the complexities of CCCs. It extensively examines how biological functions rely on CCCs to regulate crucial physiological process, including tissue homeostasis, cell development as well as immune responses.^5,24^ Furthermore, this review sheds light on the role of CCC mechanisms in regulating various diseases, which have not only expanded our understanding of CCC but also paved the way for innovative clinical treatments.

## Research history and milestone events of CCC

**Fig. 1:**
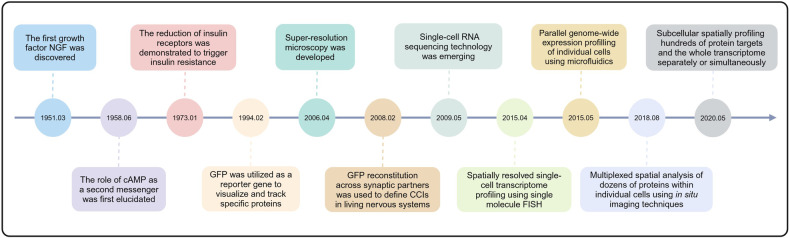
Milestone events of CCC research. Key events in the development of CCC were retrospectively summarized from 1951 to the present day. Detailed information on milestone events are narrated in this review

In multicellular organisms, cells have evolved different intercellular communication modes to develop and regulate their coordinated functions.^29^ At the macroscopic level, direct physical contacts between adjacent cells lead to the formation of tissues and barrier structures, while at the microscopic scale, they drive changes in cellular signaling pathways and activation states.^24^ Comprehending how biological components synergize to orchestrate biochemical, genetic, and biophysically mediated cell interaction events among diverse cell types constitutes the essence of enhancing our understanding of the biology underlying CCCs (Fig. 1).

Identifying cell surface receptors and their ligands, such as growth factors, is crucial for understanding how cells perceive and respond to external signals. During a research endeavor exploring the specific growth-stimulating impacts of mouse sarcoma on the sensory and sympathetic nervous systems of chick embryos, it was unveiled that mouse sarcoma had the ability to generate a specific factor to specifically promote the growth and development of nerve cells.^30^ Later, this factor is well-known as nerve growth factor (NGF) and stands as the inaugural growth factor to be discovered, unveiling the pivotal role of extracellular factors in modulating cell growth and differentiation. This revelation has exerted a profound impact on the evolution of neuroscience and cellular biology. With a deepening understanding of cellular signaling molecules, researchers have begun to investigate how signal transduction pathways alter in human diseases. Disrupting and/or altering these cell interaction events can lead to severe downstream pathophysiological effects. Research on obese hyperglycemic mouse models has revealed that insulin resistance is associated with dysfunction of insulin receptors.^31^ Insulin exerts its effects by binding to its receptors on the cell surface. Insulin resistance may be caused by a reduction of insulin receptors or receptor dysfunction, leading to decreased efficiency of insulin signal transduction. Even if insulin successfully binds to its receptor, certain components of the signaling pathway may be impaired, affecting the biological effects of insulin. The studies of the molecular mechanisms of insulin resistance enhance our understanding of how cell function and disease states can be influenced at the single-cell level by regulating the interactions between signaling molecules and receptors, offering potential new targets for the treatment of metabolic diseases. Communication errors can lead to diseases such as cancer metastasis, motor neuron diseases, virus-host interactions, and diabetes. Therefore, research into CCCs can enhance understanding of disease mechanisms and facilitate the development of pharmaceuticals.^32–35^

Optical microscopy has been widely used as a powerful means for over a century to visualize the sites of CCCs and understand the spatial or organizational structures underlying these interactions. Early examples of studying cell contact interfaces based on microscopy include the direct observation of cell dissociation in sponges and the aggregation of cells into tissue-like structures in higher animals using optical microscopy.^36,37^ With the improvement of fluorescent dyes and optical systems, scientists began to utilize fluorescent materials to study cells and tissues. An early study first reported the primary structure of the green fluorescent protein (GFP) from the jellyfish Aequorea victoria.^38^ It not only identified the amino acid sequence of GFP but also laid the foundation for subsequent research utilizing GFP as a reporter gene to visualize and track specific proteins, organelles, and intercellular communication within cells. GFP not only enables the direct observation of gene expression in living cells but also allows for the tracking of specific processes within cells through GFP-tagged proteins. Without affecting the growth and development of the host cell, the fluorescence can be stably inherited by offspring, making it an ideal tool for tracking and studying the dynamics of gene expression.^39^

Super-resolution methodologies have made significant strides, transcending this limitation through techniques such as near-field,^40^ stimulated emission depletion,^41^ structured illumination,^42,43^ and reversible saturable optical fluorescence transitions microscopy.^44^ Yet, the objective remains to achieve a fluorescence technology capable of attaining resolutions closer to the molecular scale. A technique known as GFP reconstitution across synaptic partners (GRASP) employs the fusion of two nonfluorescent split GFP fragments onto interacting partners on opposing cells to detect CCCs.^45^ Upon close contact between cells, the split proteins associate, reconstituting the GFP. This method has been expanded to include other split fluorescent protein fragments, YFP (yellow) and CFP (cyan), for the simultaneous imaging of multiple synaptic interaction factors.^46^ An advanced imaging technique known as optical super-resolution microscopy, specifically photoactivated localization microscopy, enables the observation of fluorescent proteins within cells at nanometer resolution.^47^

On the other hand, high-throughput technologies are powerful and economical tools for ultra-high-throughput transcription and protein analysis,^48–52^ which have greatly accelerated our understanding of the gene expression, regulation and network complexity of mammalian cells.^53–59^ For example, the development of Drop-Seq.^60^ and inDrop.^61^ has enabled simultaneous analysis of a large number of single cells, significantly improving sequencing efficiency and enhancing our understanding of complex tissues and cellular biology. Understanding the spatial organization of cells within tissues and how they communicate is essential for deciphering the principles of tissue architecture and organ function.^62,63^ Thus, spatial transcriptome technology, namely multiplexed error-robust fluorescence in situ hybridization (MERFISH), was developed to simultaneously measure the copy numbers and spatial distribution of hundreds to thousands of RNA species in individual cells by using single-molecule FISH fluorescent probes that bind with high specificity to the desired RNA targets.^64^ Due to its high specificity, sensitivity, and spatial resolution, MERFISH has a wide range of applications in basic biology and medical fields.^65,66^

Meanwhile, spatial proteome approaches are emerging research fields focusing on understanding the qualitative and quantitative aspects of protein composition within single cells. Immunohistochemistry and immunofluorescence techniques, combined with microscopic imaging technology, can display the distribution and density of cells in tumor tissue samples, as well as the physiological and biochemical activities involved by different cells.^67,68^ For example, CODEX (CO-Detection by indEXing)^62^ and Cell DIVE^69^ are cutting-edge high-dimensional imaging technologies that have revolutionized the study of cell communication and tissue analysis. The core design principle of CODEX is to label each antibody with a specific oligonucleotide “barcode”, of which the complementary sequence is bound with the fluorescent dyes used for subsequent imaging.^62,70^ In contrast, each antibody used in Cell DIVE is directly labeled with fluorescent dyes, followed by multiple rounds of staining, imaging and fluorescence quenching. Therefore, both CODEX and Cell DIVE offer high-dimensional imaging of dozens of proteins within individual cells, enabling researchers to analyze the spatial organization of cells, their interactions, and signaling states within tissues. Furthermore, novel single-cell spatial in situ imaging technologies, such as GeoMx DSP spatial multi-omics technology, break through the limitations of the number of detected proteins and enable spatially profiling 570+ protein targets and the whole transcriptome separately or simultaneously.^63,71^ Collectively, these advanced spatial single-cell imaging technologies will drive deeper insights for cell typing, tissue phenotyping, cell–cell interactions, cellular processes, and biomarker discovery.

## Molecular mechanism of CCC

**Fig. 2:**
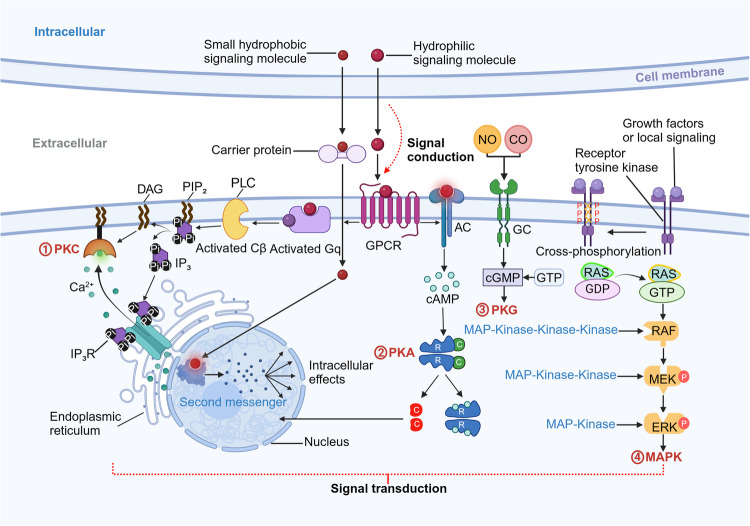
Representative signal pathways of CCC. Cellular communication is the process of signal construction to signal transduction. The interactions of ligands and receptors usually affect cell metabolism and energy transformation of different cell types to maintain the normal function of organisms. Ligands are active substances capable of specifically binding to receptors. Receptors specifically recognize and bind to signaling molecules, converting them into intracellular signals to perform specific physiological functions. One of the major signaling pathways within the signal transduction pathway are GPCRs pathways, including PKA and PKC systems. The others are enzyme-coupled receptor pathways, including PKG and MAPK systems. AC adenylate cyclase, cAMP cyclic adenosine monophosphate, cGMP cyclic guanosine monophosphate, CO carbon monoxide, DAG diacylglycerol, ERK extracellular regulated protein kinases, GC guanylate cyclase, GDP guanosine-5’-diphosphate, GPCR G-protein-coupled receptor, GTP guanosine triphosphate, IP3 inositol trisphosphate, IP3R inositol trisphosphate receptor, MAPK mitogen-activated protein kinase, MEK mitogen-activated extracellular signal-regulated kinase, NO nitric oxide, Pi PIP2 phosphatidylinositol-4,5-bisphosphate, PKA protein kinase A, PKC protein kinase C, PKG protein kinase G, PLC phospholipase C

Cell signaling, which involves transmitting information between cells or subcellular components, is an inherent characteristic of living organisms. In diverse tissues and organs, cell signaling facilitates communication and homeostasis, which are vital for cellular interactions within their local environment. Signals can propagate through various mechanisms, including chemical alterations, mechanical forces, or their synergistic effects. A multitude of extracellular signals and cellular membrane proteins trigger selective intracellular pathways, influencing crucial cellular outcomes such as survival, apoptosis, growth, motility, differentiation, and specific functions like muscular contraction, synaptic activity, or thrombocyte activation.^72^ Typical examples are the triggering of the transforming growth factor-β (TGF-β) signaling pathway in the context of tissue fibrosis.^73^ and the excessive activation of the Ras signaling pathway in numerous cancer types.^74^ Over the last decades, extensive research into cell signaling pathways has culminated in the creation of therapeutics rooted in biological science, and the complexity revealed by drugs currently in clinical use continues to uncover further insights into the extent of interactions between signaling networks.^75^ Cellular communication encompasses two essential processes: cell signal conduction, focusing on the generation and intercellular transmission of signals, and cell signal transduction, which emphasizes the reception of signals and how signals are converted and processed upon receipt (Fig. 2).

### Mechanisms of CCC in physiological homeostasis

Cellular communication involves cells recognizing, receiving, and responding to external signal molecules, which can be light, electricity, or chemical molecules. Finally, the interaction of receptors can activate a series of downstream physiological and biochemical processes, which play an important role in coordinating cell function. Cellular communication involves the transmission of signals from signal generation to signal transduction. Cell signal transduction is the process through which biological information (excitation or inhibition) is transformed and transmitted between cells or within cells, leading to various biological effects. This typically refers to transmembrane signal transduction, wherein bioactive substances activate or inhibit cell function through receptors or ion channels. Generally, the chemical substances involved in intercellular signal communication or intracellular signal transduction are known as signal molecules, while small molecules specialized in carrying biological information are called messenger molecules. The chains of signal molecules that complete the conversion and transmission of biological information within or between cells are referred to as signal transduction pathways. Therefore, the essence of cell signal transduction lies in the intracellular transformation and transmission of biological information through specific signal transduction pathways, which may involve regulating the gene expression processes of related functional proteins.

#### The steps of cell signal conduction

##### Production and release of signaling molecules

Certain cells, such as neurons or endocrine cells, react to external stimuli or internal needs by creating and releasing signaling molecules like neurotransmitters, hormones, or chemokines. These molecules serve as messengers to communicate messages to neighboring cells.

Neurons, as the primary cells that participate in information processing, come in a myriad of distinct cell types differentiated by morphology, location, connectivity, and chemical properties.^76^ The various chemicals that transmit information between neurons are known as neurotransmitters. Owing to the central role of neurotransmitters in cerebral function, neurotransmitter receptors along with other proteins engaged in the synthesis and deactivation of neurotransmitters emerge as critical targets in the development of curative medications for mental and nerve disorders, ache and many other conditions.^77^ As gaseous neurotransmitters, such as nitric oxide (NO) and carbon monoxide (CO), play a regulatory role in vasodilation and neural transmission.^78,79^ The nervous system typically enables information to be transmitted rapidly between different regions of the body.

In contrast, hormonal communication is predicated on the synthesis and dissemination of a plethora of glandular hormones, coupled with their transportation via the bloodstream, making it more suited to situations requiring broader and more sustained regulatory actions. These two systems of communication are mutually complementary, with neural stimuli capable of affecting the secretion of certain hormones, and conversely.^80^ Certain hormones are tailored to interact exclusively with a limited array of target cells, whereas others exert influence across a broad spectrum of cell types throughout the organism. To preserve homeostasis and adapt effectively to environmental alterations, the biosynthesis and release of hormones are subject to rigorous regulation. This regulatory mechanism is achieved through a complex interplay among multiple hormones, which reciprocally regulate one another, rather than being governed by a solitary hormone. Hormones play a pivotal role in orchestrating a multitude of bodily functions, encompassing growth and development, metabolic processes, electrolyte equilibrium, and reproductive functions.

Cytokines are produced by specific cells (such as immune cells, endothelial cells, etc.) in response to specific stimuli, such as infection, injury, inflammatory responses, or the action of other cytokines, and are released into the extracellular environment. Cytokines are capable of activating a variety of cell types within a specific tissue or initiating diversified signaling pathways within a specific cell type, exemplified by interleukins and interferons, regulating various physiological processes, encompassing immunity, development, growth, and tissue repair.^81,82^ Serving as pivotal mediators of intercellular communication within the immune system, dysregulation in cytokine expression or their intracellular signaling pathways disrupts immune homeostasis, precipitating the onset of pathologies such as chronic inflammation, autoimmune syndromes, and malignant tumors.^83^

The production of signaling molecules initiates with gene expression. Specific stimuli, such as extracellular signals or changes in internal states, trigger the transcription and translation of specific genes, leading to the production of proteins or small molecules as signaling molecules. Prior to maturation and activation, these protein signaling molecules typically go through a sequence of post-transcriptional modifications (such as splicing) and posttranslational modifications (such as phosphorylation and glycosylation). Meanwhile, some small-molecule signaling substances are synthesized within the cell through specific biochemical pathways. Before their release, signaling molecules accumulate and are stored in specific organelles within the cell. For instance, neurotransmitters are usually stored in synaptic vesicles of presynaptic neurons.^84^

When cells receive stimuli to release signaling molecules, such as electrical, chemical, or mechanical signals, the vesicles storing these signaling molecules move to the vicinity of the cell membrane and fuse with it. Through exocytosis, the signaling molecules are released outside the cell, a process that is particularly important for proteins and certain large molecular signaling molecules. On the other hand, some small molecules and lipophilic signaling molecules directly pass through the cell membrane to enter or exit the cell without vesicle-mediated processes. Besides NO and CO, hydrogen sulfide is another well-recognized gaseous signaling molecule.^85^ These gaseous molecules are unique in their mode of action as they can freely diffuse across cell membranes, enabling rapid signaling without the need for specific receptors or transport mechanisms for their release and action.

##### Transmission of signaling molecules

Signaling molecules can reach target cells via diffusion, blood circulation, direct contact, or intercellular junctions. In the context of multicellular organisms, four fundamental forms of intercellular signaling exist: autocrine signaling, paracrine signaling, signaling through gap junctions, and endocrine signaling. Autocrine signaling is prevalent in tumor cells, wherein cells secrete ligands to induce responses via homologous receptors expressed on the same cell. Paracrine signaling affects nearby cells by secreting local chemical mediators into the extracellular fluid, which act on adjacent target cells through local diffusion. Signal transmission across gap junctions involves molecules passing directly between cells. Endocrine signaling occurs when endocrine cells secrete substances into the bloodstream, distributing them to various body parts via circulation.

For long-distance signals, the bloodstream is the primary mode of transmission. Endocrine cells regulate the production and release of hormones by monitoring the concentration of specific substances in the blood, such as glucose, electrolytes, and other hormones. These hormones are then transported to various parts of the body through the bloodstream, exerting regulatory effects on distant target cells, thereby maintaining physiological equilibrium and adapting to changes in the external environment.^86^ For local signals, transmission through intercellular spaces or direct cell contact is more common. Neurotransmitters primarily transmit information through paracrine signaling. They are produced and released by neurons at the presynaptic end, and transmitted across the synaptic gap to the adjacent postsynaptic neuron or effector cell. Once released, neurotransmitters diffuse across synapses. In conjunction with the primarily chemical synapses, electrical synapses also exist, facilitating the intercellular flow of ions via gap junctions. Electrical synapses enable the transmission of simple electrical signals among neurons, while chemical synapses allow excitation, inhibition, and complexity of biochemical information to be passed among cells.^77^ Cytokines typically act on neighboring cells through paracrine signaling, especially during immune responses and inflammation. However, in some cases, they can also be released into the bloodstream to regulate systemic immune responses and inflammation. Certain cytokines that exist in a membrane-bound form require direct contact with receptors on adjacent cells to transmit signals.^87,88^

##### Binding of signaling molecules to receptors

Signaling molecules bind specifically to receptors on the surface or inside the target cell, forming receptor-ligand complexes that activate the receptor. Receptors are proteins or glycoproteins designed to recognize and bind specific signaling molecules, converting external signals into internal ones. Hormones travel to target cells via the bloodstream and bind to specific receptors located on the cell surface or within the cell. These receptors have binding sites that are highly complementary to the hormone molecules, allowing hormones to specifically recognize and bind to their receptors. Upon binding to its receptor, the hormone typically induces a conformational change in the receptor, thereby activating it. For cell surface receptors, this conformational change can directly trigger intracellular signaling pathways. For intracellular receptors such as steroid hormone receptors, the hormone–receptor complex often translocates to the nucleus, interacts with DNA, and influences the expression of specific genes.

Neurotransmitter molecules, liberated from presynaptic vesicles, traverse the synaptic cleft and bind to proteins on the postsynaptic neuron’s surface membrane known as neurotransmitter receptors, altering the function of the postsynaptic neuron. There are two types of neurotransmitter receptors: ligand-gated ion channel receptors (LGICs) and G-protein-coupled receptors (GPCRs). LGIC receptors are proteins specifically designed to rapidly transduce chemical signals of neurotransmitters directly into electrical responses.^89,90^ A part of the protein is dedicated to binding with neurotransmitter molecules on the extracellular side of the protein. The portion of the protein embedded within the cell membrane acts as an ion channel, creating a fluid-filled passage in the membrane for the transit of charged ions, but ions are unable to pass across lipid or other solid membrane constituents. This synaptic transmission modality allows for the direct flow of ions across the outer cell membrane rapidly. When a neurotransmitter binds to the receptor, the exchange of nucleotide guanosine-5’-diphosphate (GDP) and guanosine triphosphate (GTP) on the protein’s intracellular side is expedited, culminating in the split of the G protein into α- and β/γ-subunits, both two types of subunits can act upon “effector” proteins, altering cellular biochemistry, physiology, and gene expression, initiating intracellular chemical signaling events.^84,89,90^

Cytokines are classified as secreted growth factors that instigate signal transduction within target cells through binding with the extracellular domains of cell surface receptors, forcing receptor dimerization.^91^ Most cytokines contain binding sites for both high-affinity and low-affinity receptors.^92,93^ Typically, the high-affinity receptor subunit acts as a cytokine-specific, private receptor that determines cell specificity as well as the cytokine’s dose sensitivity.^94^ In contrast, the low-affinity receptor subunits are common to be shared among various cytokines and mainly have an impact on the efficiency of complex assembly, thereby affecting the maximum strength and duration of receptor signaling.^92,95^

#### The steps of cell signal transduction

##### Signal transduction and amplification

The activation of receptors sets off a cascade of cellular responses internally. Signals are transduced and amplified within the cell through mechanisms like second messengers, switch proteins, enzyme cascades, etc. Second messengers are small molecular compounds that carry information within the cell. The second messengers play a crucial role in cell signaling, capable of transforming the activation of a cell surface receptor into the activation of multiple molecules within the cell, thereby amplifying and conveying signals internally. This process activates or inhibits specific target proteins and enzymes, triggering a cascade of downstream reactions. Through these reactions, second messengers are instrumental in regulating a myriad of cellular functions. The discovery of this signal transduction pathway has laid the foundational framework for understanding how cells communicate via chemical signals.

An early study elucidated for the first time the role of cyclic adenosine monophosphate (cAMP) as a second messenger within the cell, demonstrating its capacity to translate the cell surface receptor’s response to hormones and neurotransmitters into intracellular biochemical activities.^96^ When external signaling molecules such as adrenaline bind to GPCRs, the associated G proteins are activated. The α subunit of the G protein activates adenylate cyclase (AC), converting ATP into cAMP. cAMP, serving as a second messenger, activates protein kinase A (PKA). In cardiac cells, the increase in cAMP leads to the activation of PKA, which phosphorylates L-type calcium channels, increasing the influx of calcium ions, thereby enhancing the contractile force of the heart.^97–99^

Calcium ions (Ca^2+^) are important second messengers involved in various signaling pathways. When external signals such as neurotransmitters or hormones act on cells, the opening of calcium channels or the release of Ca^2+^ from the endoplasmic reticulum can cause an increase in intracellular Ca^2+^ concentration. Subsequently, Ca^2+^ binds to calmodulin and then activates downstream enzymes like Ca/calmodulin-dependent protein kinase II (CaMKII), affecting various processes within the cell. During neurotransmission, the release of neurotransmitters causes the opening of calcium channels on the postsynaptic membrane, allowing Ca^2+^ to flow into the cell, activating signaling pathways related to learning and memory, such as the activation of CaMKII, which promotes the strengthening of synapses.^100–102^

Switch proteins, like G proteins and Ras, regulate the opening and closing of signaling pathways. Ras protein is a small GTPase involved in regulating signaling pathways for cell proliferation and differentiation. When growth factors such as epidermal growth factor (EGF) bind to their receptor epidermal growth factor receptor (EGFR), EGFR activates Ras, causing Ras to switch from a GDP-bound state to a GTP-bound activated state.^103,104^ The activation of Ras promotes the activation of the mitogen-activated protein kinase (MAPK) or extracellular signal-regulated kinase (ERK) pathway, affecting the expression of cell cycle regulatory proteins such as cyclin D, and facilitating the cell to enter a proliferative state.

Enzyme cascades involve a series of enzymes that activate or inhibit each other, amplifying and regulating signals (e.g., PKA, MAPK). In the MAPK pathway, Raf (MAPKKK) activates MEK (MAPKK), which in turn activates ERK (MAPK). Each step of activation can lead to the phosphorylation of multiple downstream proteins. In response to cellular stress, the p38 MAPK pathway is activated, leading to an increase in the expression of inflammatory response proteins such as tumor necrosis factor α (TNF-α), participating in the cell’s response to stress and inflammation.^105^

##### Cell response

Signal transduction ultimately influences the cell’s physiological functions, including changes in metabolic activity, regulation of gene expression, alterations in shape, or movement. Different cell types and states may exhibit varying responses to the same signaling molecule. Insulin activates the phosphoinositide 3-kinase (PI3K)/Akt (also known as Protein Kinase B, PKB) signaling pathway through the insulin receptor. Then, PI3K/Akt signaling pathway can promote the surface expression of the glucose transporter GLUT4 and increase cellular glucose uptake, supporting energy production, and muscle contraction.^106–108^ Similarly, in liver cells, insulin also enables the activation of the PI3K/Akt signaling pathway and thus promotes the activation of glycogen synthase, increasing glycogen synthesis, and lowering blood glucose levels.^109^ In contrast, TNF-α can activate the nuclear factor kappa-light-chain-enhancer of activated B cells (NF-κB) signaling pathway through its receptor and then promote the expression of inflammatory response proteins, participating in immune responses and cell repair processes.^110^ However, in some cancer cells, the same signaling pathway may promote the survival and proliferation of cancer cells because these cells may have acquired resistance to apoptosis signals induced by TNF-α.^111^

##### Termination or reduction of signaling response

To maintain the cell’s sensitivity and adaptability to external stimuli, responses to signaling molecules must be terminated or reduced. This can be achieved through receptor desensitization, receptor downregulation, degradation, hydrolysis of second messengers or switch proteins, and negative feedback regulation, which involves downstream effector molecules inhibiting upstream signaling molecules, forming a closed loop. Prolonged exposure to high concentrations of agonists (such as adrenaline) leads to desensitization and downregulation of the corresponding GPCRs. The desensitization process often involves phosphorylation of the receptor, which attracts β-arrestin proteins to bind with the receptor, preventing further activation of G proteins while promoting receptor internalization.^112,113^ The internalized receptors may be transported to lysosomes for degradation (downregulation), or be dephosphorylated and recycled back to the cell surface. This process leads to the decrease of receptors on the cell surface, reducing the cell’s sensitivity to the agonist.

The rise and fall of cAMP levels are crucial for signal transmission. The degradation of cAMP is catalyzed by cAMP phosphodiesterase, which converts cAMP to AMP, thereby terminating the signal transmission mediated by cAMP.^114,115^ The MAPK/ERK signaling pathway acts importantly in cell proliferation and differentiation. Activation of this pathway promotes the phosphorylation of a sequence of downstream effector molecules, consisting of transcription factors, thus affecting gene expression. Meanwhile, the MAPK pathway also activates certain phosphatases, such as DUSP (dual-specificity phosphatases), which can dephosphorylate and inhibit components of the MAPK pathway, such as ERK, forming a negative feedback loop to set limits on signal strength and duration, preventing overreaction.^116^ These mechanisms together ensure a dynamic balance of signal transduction, allowing cells to make adaptive adjustments to continuous or excessive stimuli, maintaining the normal functioning of physiological functions.

### Major signaling pathway

Within the signal transduction pathway, a receptor is a protein within a cell responsible for receiving and transducing information. Receptors in the cell membrane are termed membrane receptors, while those in the cytoplasm and nucleus are known as cytoplasmic and nuclear receptors, respectively. Ligands are active substances capable of specifically binding to receptors. Receptors specifically recognize and bind to signaling molecules, converting them into intracellular signals to perform specific physiological functions. Cell signals typically begin with primary messengers like growth factors, hormones, and ions instigate a sequence of signal transduction processes via membrane-bound or intracellular receptors. This mechanism incorporates multiple feedback systems and many intracellular chemicals classified as second messengers, consisting of cAMP, cyclic guanosine monophosphate (cGMP), calcium ions, etc.^117^

The conjunction of a ligand with its specific receptor triggers a distinct cellular signaling route.^118^ There are two types of signal transduction pathways based on the nature of the ligand and receptor. One is the nuclear receptor-mediated signal transduction, wherein lipid-soluble ligands enter the cell through simple diffusion and directly bind to cytoplasmic or nuclear receptors, typically influencing gene expression. Another type involves water-soluble ligands or physical signals, which initially act on the membrane receptor and produce effects through transmembrane and intracellular signal transduction mechanisms. These signal transduction receptors include various types, including GPCRs, enzyme-coupled receptors and ion channel-linked receptors. It should be noted that most membrane receptor-mediated signal transduction pathways can also alter transcription factor activity and affect gene expression.

#### GPCRs pathway

GPCRs constitute the most extensive and varied type of membrane receptors in eukaryotic system. G proteins composed of three subunits separately: alpha (α), beta (β), and gamma (γ) are unique proteins that bind to nucleotides like GTP and GDP. The α and γ subunits are tethered to the cell membrane via lipid links. Upon ligands attachment to a GPCR, ligands change the GPCR conformation, leading to the activation of the G protein. The active G-protein disassociates from the receptor, splitting into α and β/γ subunits. These subunits then stimulate specific effectors, leading to the generation of second messengers, which are detected by various protein kinases, ultimately triggering a cascade of signaling events that drive cellular responses.

GPCRs play a critical role in cellular detection of external stimuli, including odorants, taste compounds, photons, metals, neurotransmitters, biogenic amines, fatty acids, amino acids, peptides, proteins, steroids, and lipids. The vast array of potential ligands and receptors links GPCRs to numerous physiological and pathological states, including pain, asthma, cancer, cardiovascular, gastrointestinal, and neurological disorders. This significance renders GPCRs as prime targets for pharmaceutical intervention.^119^ For instance, the identification of spontaneous GPCR mutations in individuals with various endocrine disorders highlights their importance in endocrinology.^120^

The GPCR pathway regulates multiple signaling cascades, notably involving the PKA system, inositol trisphosphate (IP3) pathway, and the calcineurin-dependent protein kinase (Ca/CaM) pathway. These three pathways together constitute the main framework of GPCRs signal transduction. Through different second messengers and effector proteins, GPCRs participate in adjusting numerous physiological functions of the cell.

The PKA system operates within the cyclic nucleotide system, where extracellular signals bind to corresponding receptors. This activates a signaling pathway that elicits a response by regulating the level of cAMP. Typically, the signaling molecules are hormones, and the cAMP levels are regulated by adenylate cyclase. Following the action of the signaling molecule on the membrane receptor, the G-protein-coupling system is activated. Once cAMP is generated, it will activate PKA to amplify the signal. This PKA signal transduction pathway regulates membrane protein activity, gene expression, and hormone synthesis as well as hormone secretion.^121^

IP3 pathway involves inositol trisphosphate as a second messenger in G-protein-coupled receptor-mediated signal transduction. In the IP3 pathway, extracellular signaling molecules bind to corresponding GPCRs, activating the Gq protein on the membrane. Subsequently, the Gq protein activates Cβ which is the one kind of the isoforms of protein kinase C (PKC) and can dissolve phosphatidylinositol-4,5-bisphosphate (PIP2) into two second messengers. The second messenger IP3 binds to its receptor, promoting the release of Ca^2+^. Another second messenger, diacylglycerol (DAG), synergistically activates PKC along with Ca^2+^ and phosphoacylserine, culminating in a cascade reaction that drives cellular responses.^122^ PKC enzymes take a significant part in the process of cell proliferation, differentiation, and apoptosis. Ca^2+^ plays an important role in neuronal cells, mediating essential physiological processes.^123^ Thus, this pathway contributes to the regulation of cell proliferation, metabolism, and growth, making it a potential target for tumor inhibition and myocardial protection.

Ca/CaM pathway is activated through a series of interactions involving calcium ions, calmodulin, and the phosphatase activity of calcineurin itself. This pathway involves the release of calcium ions from the endoplasmic reticulum or the opening of calcium ion channels on the cell membrane, resulting in an increase in the concentration of calcium ions in the cytoplasm. The calcium ions then bind to calmodulin, thereby activating the phosphorylation activity of protein kinases. This activation can affect the physiological functions of the cell by regulating the phosphorylation state of substrates.^124,125^

#### Enzyme-coupled receptor pathway

Enzyme-linked receptors, commonly single-pass transmembrane proteins, have enzymatic activity within their intracellular regions or directly interact with proteins that have enzymatic activity. The activity of enzyme-linked receptors is strictly regulated, including ligand-binding-induced receptor dimerization, phosphorylation, and negative feedback mechanisms. Ligands, such as growth factors, coupling with the extracellular domain of the receptor, inducing receptor dimerization or oligomerization, thereby activating its intracellular enzymatic activity. Following receptor dimerization, the intracellular enzymatic domains phosphorylate each other, activating the receptor’s enzymatic activity.^126–128^ The activated receptor transmits signals by phosphorylating downstream proteins (such as protein kinases and transcription factors), triggering a series of signal cascade reactions that ultimately lead to changes in the expression of specific genes and regulation of cell behavior. The termination of signal transduction is usually achieved through mechanisms such as receptor dephosphorylation, endocytosis, and degradation, ensuring the precision and timeliness of signal transmission. By activating a variety of downstream signaling molecules and pathways, enzyme-linked receptors participate in the widespread regulation of physiological functions, including cell proliferation, differentiation, migration, metabolism, and survival.

Enzyme-coupled receptors possess distinct molecular structures and properties compared to GPCRs. For instance, tyrosine kinase receptors possess protein tyrosine kinase (PTK) activity. When a hormone binds to the receptor, the PTK segment in the membrane is activated. This activation initiates a series of intracellular information transmission cascade reactions with phosphorylation of peptide chains and tyrosine residues in the membrane protein substrate.^129^ Ultimately, gene transcription processes change within the nucleus and result in corresponding biological effects within the cell. Most growth factors, insulin, and some peptide hormones are transmitted through this receptor type. It has been reported that PTK is pivotal in initiating multicellular responses related to DNA synthesis and cell proliferation. The proteins encoded by many retrovirus oncogenes and the intracellular regions of several growth factor transmembrane receptors exhibit PTK activity. The receptor PTK not only plays a role in transmitting extracellular information, such as hormones and growth factors, but is also involved in the malignant transformation and proliferation of cells. PTK has been identified and characterized as a selective, potent, and well-tolerated kinase inhibitor suitable for cancer therapy.^130^ As PTK takes a critical part in the development and progression of tumors, it serves as a promising therapeutic target in cancer cells. Gene-targeting medications available commercially can effectively reach therapeutic objectives by suppressing its function.

There are many other types of enzyme-linked receptors, among which the more important ones include receptor tyrosine kinases (RTKs) and guanylate cyclase (GC) receptors. RTKs refer to receptors whose intracellular part of the membrane itself possesses tyrosine kinase activity. The extracellular signaling molecules that can bind to these receptors and complete signal transduction are mainly various growth factors, such as epidermal growth factor, platelet-derived growth factor, fibroblast growth factor, and insulin. When the extracellular part of the receptor binds to a ligand, the activation of the tyrosine kinase in the cytoplasmic part of the receptor molecule occurs, thereby triggering various signaling proteins to transmit signals along different pathways reviewed below.

The Ras-MAPK is an important signaling pathway mediated by RTKs, mainly composed of three key kinases: Ras, Raf, and MAPK. When cells are stimulated by growth factors such as EGF, PDGF, the growth factor receptors (e.g., EGFR, PDGFR) are activated, leading to the accumulation of GTP-bound Ras (Ras-GTP). Downstream Raf kinase is then activated by Ras-GTP on the cell membrane, phosphorylating and activating MAPKK (MEK), which in turn phosphorylates and activates MAPK (ERK).^131^ MAPK is able to phosphorylate downstream substrates, which are often transcription factors (such as Elk1, Fos, Jun) or other proteins that change cell behavior. The aberrant activation of the Ras-MAPK signaling pathway has a bearing on many diseases, including cancer and neurodegenerative diseases.^132,133^ Moreover, this pathway also interacts with other signaling pathways such as PI3K–Akt, Janus kinase (JAK)-signal transducers and activators of transcription (STAT), etc., jointly regulating processes such as cell growth, proliferation, and differentiation.

Another pathway activated by RTKs is the PI3K–Akt signaling pathway. When ligands such as growth factors bind to RTKs, the RTKs undergo autophosphorylation and activation, which prompts the recruitment and activation of PI3K near the receptor. The activated PI3K converts the membrane lipid PIP2 into phosphatidylinositol-3,4,5-trisphosphate (PIP3). PIP3, acting as a second messenger, activates Akt, thereby initiating a series of downstream reactions that affect cell survival, proliferation, growth, and metabolism.^134,135^

GC receptors have a single transmembrane α-helix, with the N-terminal ligand-binding site located on the extracellular side and the C-terminal GC domain located on the intracellular side. Once the receptor binds with a ligand, GC activity is activated. Unlike the activation of AC, this process does not require the involvement of G proteins. Once activated, GC catalyzes the conversion of GTP to cGMP within the cytoplasm, which then bind and activate cGMP-dependent protein kinase G (PKG). Similar to PKA and PKC, PKG is a serine/threonine protein kinase that mediates signal transduction through the phosphorylation of substrate proteins. Upon activation by NO and CO, GC augments the production of cGMP. The cGMP binds and activates PKG, which phosphorylates substrate proteins, activating downstream signaling pathways to regulate cell growth and renewal.^136^ The PKG signal transduction system regulates smooth muscle relaxation, nervous system function, and physiological processes such as intestinal secretion, renin release, bone growth, and visual signal transduction.^137,138^ Besides, there are signal transduction pathways directly initiated or propagated by intracellular functional compartments. For instance, the receptor for NO is a type of GC located within the cytoplasm, known as soluble GC. When NO acts on soluble GC, it increases the concentration of cGMP and the activity of PKG within the cytoplasm, leading to responses such as the relaxation of vascular smooth muscle.^139,140^ These pathways are essential for coordinating the activities of each organelle with other cellular components.

In addition to the signaling pathways mentioned above, enzyme-linked receptors also mediate the JAK/STAT pathway and the TGF-β-Smad pathway. The JAK-STAT signaling pathway is typically activated by cytokine receptors, which lacks intrinsic tyrosine kinase activity, but interacts with members of the intracellular tyrosine kinase family JAKs. When cytokines (such as interferons and interleukins) bind to their specific receptors, they promote the activation of JAKs. The activated JAKs phosphorylate the receptors, providing docking sites for STATs to bind and become activated. The activated STATs dimerize and then translocate to the nucleus, where they directly regulate the expression of target genes.^141^ Besides, the TGF-β signaling pathway is primarily mediated by a class of receptors known as Serine/Threonine Kinase Receptors, which possess serine/threonine kinase activity in their intracellular region. When ligands of the TGF-β family, such as TGF-β, bone morphogenetic proteins (BMPs), activins, etc., bind to these receptors, they prompt the receptor kinases to phosphorylate Smad proteins. The phosphorylated Smad proteins further interact with other Smad proteins or DNA-binding proteins, transmitting the signal from the cell membrane to the nucleus, thereby regulating the expression of specific genes.^142,143^

#### Other pathways

External signaling molecules trigger the proteolytic cleavage of a potential gene regulatory protein. Controlled proteolysis modulates the expression of target genes. Signal transduction pathways relying on regulated proteolysis encompass Notch pathway, Hedgehog (Hh) pathway, Wnt pathway and NF-κB pathway.

The Notch signaling pathway is a highly conserved intercellular communication mechanism that is extensively involved in various biological processes, including cell fate determination, embryonic development, and tissue regeneration.^144–146^ A distinguishing trait of this signaling pathway is its reliance on direct cell–cell contact, obviating the requirement for signal molecules to travel long distances between cells. The Notch signaling pathway is initiated by the direct binding of the Notch receptor located on the surface of the signal-receiving cell to its ligand situated on the surface of neighboring cells. Notch receptors and ligands are both transmembrane proteins, with typical ligands belonging to the Jagged and Delta families. After ligand–receptor binding, the Notch receptor undergoes a series of cleavage processes. First, the ADAM (a disintegrin and metalloprotease) family member metalloproteases cleave the Notch receptor in the extracellular region, followed by cleavage by the γ-secretase complex in the transmembrane region, leading to the release of the Notch intracellular domain (NICD).^147^ The released NICD then enters the nucleus, where it binds to the CSL (CBF1/Su(H)/Lag-1) family of DNA-binding proteins and other co-activators to form a transcriptional activation complex, directly regulating the expression of downstream genes.^148,149^

The Hh signaling pathway is a key intercellular signal transduction mechanism, extensively involved in the development of animal embryos and the maintenance of adult tissues. This pathway plays a crucial role in the growth and proliferation of cells during embryonic development and after embryo formation. Aberrant activation of the Hh signaling pathway is associated with various cancers and developmental abnormalities.^150^ Before being secreted outside the cell, Hh proteins undergo a series of posttranslational modifications, including autocleavage and covalent attachment to cholesterol.^151^ In the absence of Hh ligands, the Patched (Ptch) receptor inhibits the activity of Smoothened (Smo). When a Hh ligand binds to Ptch, this inhibition is lifted, allowing Smo to be activated. The activation of Smo triggers a series of intracellular signaling events, ultimately affecting the activity of glioma-associated oncogene homolog (Gli) transcription factors. In the absence of signaling, Gli factors are partially degraded into a repressor form. When the Hh signal is activated, the inhibition of Gli is removed, allowing its full-length form to enter the nucleus. The activated Gli transcription factor enters the nucleus and promotes the expression of downstream target genes which take part in processes such as cell proliferation, differentiation, and survival.

The Wnt signaling pathway is a complex cell signaling system, extensively involved in the embryonic development, cell proliferation, migration, differentiation, and maintenance of adult tissue homeostasis in animals. The name of this pathway originates from a gene discovered in fruit flies called “wingless” and its mouse homolog “Int-1”, collectively known as Wnt.^152^ The Wnt signaling pathway is primarily divided into two pathways: the β-catenin-dependent canonical pathway and the β-catenin-independent noncanonical pathways. In the absence of Wnt ligands, β-catenin is captured in the cytoplasm by a complex (including proteins such as Axin and GSK-3β) and phosphorylated by GSK-3β, leading to its ubiquitination and degradation. When Wnt signals are present, Wnt ligands bind to the Frizzled receptor and LRP5/6 co-receptor, inhibiting the β-catenin degradation complex, preventing the phosphorylation and subsequent degradation of β-catenin. The stabilized β-catenin accumulates and translocates to the nucleus, where it binds to transcription factors of the TCF/LEF family, activating the expression of downstream target genes.^153^ The noncanonical pathways do not rely on β-catenin but are mediated by other signaling molecules such as Ca²^+^, JNK, Rho GTPase, etc., inducing various cellular responses, including cell polarity, movement, and tissue morphogenesis.^153^

The NF-κB signaling pathway is a key cellular signal transduction mechanism, extensively involved in regulating immune responses, inflammatory reactions, cell survival, proliferation, and differentiation among various biological processes.^154–156^ Serving as an immediate response mechanism, it can rapidly respond to a wide range of external stimuli, such as cytokines, pathogens, free radicals, and other stress signals. The NF-κB pathway can be activated by multiple signals, including TNF-α, interleukin 1 (IL-1), lipopolysaccharides (LPS), viral infections, and other stress conditions. When inactive, NF-κB is bound to its inhibitory protein IκB in the cytoplasm. Upon activation by the aforementioned signals, the IκB kinase (IKK) complex is activated, leading to the phosphorylation of IκB and its subsequent degradation via the ubiquitin-proteasome pathway. The degradation of IκB releases NF-κB, allowing it to translocate to the nucleus, bind to κB sites on DNA, and activate the transcription of specific genes.^157^ The termination of the NF-κB signal involves newly synthesized IκBα, which can enter the nucleus, bind to NF-κB, and export it back to the cytoplasm, thereby returning NF-κB to an inactive state. Accurate control of the NF-κB signaling pathway is crucial for maintaining normal cellular functions and preventing the development of disorders, including cancer, autoimmune diseases, and chronic inflammatory diseases.^158,159^ This underscores its significance as a prime target for pharmaceutical interventions.

### Multi-level regulation of CCC and its implications

#### Upregulation and downregulation

The activity of CCC signal transduction can be modulated through upregulation (enhancing signal transmission) and downregulation (weakening signal transmission). This regulation can be achieved by altering the expression level of receptors, modulating receptor activity, or changing the availability of signaling molecules. In certain inflammatory responses, cytokines such as TNF-α induce the upregulation of adhesion molecules, such as intercellular adhesion molecule-1 (ICAM-1) and vascular cell adhesion molecule-1 (VCAM-1), which are located on the surface of endothelial cells.^160–162^ This upregulation enhances the interaction between white blood cells and endothelial cells, promoting the migration of white blood cells and inflammatory responses.^163–165^ The prolonged or excessive use of β-adrenergic receptor (β-AR) agonists for asthma treatment leads to a reduction in the quantity of β-ARs on cardiac and smooth muscle cells (SMCs), achieved through mechanisms of receptor internalization and degradation.^166–168^ GPCRs after being activated over a long period can be internalized through a β-arrestin-mediated pathway. This process gives rise to a decrease in the number of receptors on the cell surface, leading to a dampened receptor activity and ultimately impairing signal transmission.^113,169–171^ This phenomenon diminishing the cell responsiveness to the agonist commonly termed receptor downregulation.^172^

#### Desensitization

Long-term or excessive signal stimulation causes cells to become desensitized to certain signals. Desensitization is a protective mechanism to prevent overreaction, achieved by reducing the surface expression of receptors or inhibiting the activity of signal transduction components. In patients with type 2 diabetes, prolonged high levels of blood glucose and insulin can lead to desensitization of insulin receptors, reducing their sensitivity to insulin and further exacerbating insulin resistance.^173^ In addition, long-term alcohol consumption can increase the nervous system’s tolerance to alcohol, achieved by regulating the expression and sensitivity of neurotransmitter receptors such as GABA receptors and glutamate receptors.^174–177^

#### Upstream regulators and downstream effectors

The regulation of signaling pathways involves multiple upstream regulators and downstream effectors. Upstream regulators are responsible for receiving and integrating external signals, while downstream effectors execute the biological effects of these signals, such as altering gene expression and regulating cell behavior. These signaling pathways are key mechanisms for communication between cells, regulating cell behavior and cell fate through the reception and transmission of external signals. They play roles in a variety of biological processes, including cell proliferation, differentiation, migration, cell death, and the maintenance of tissue and organ homeostasis. The aberrant activation or inhibition of these pathways is closely related to the development of various diseases, especially cancer, inflammatory diseases, neurodegenerative diseases, and congenital developmental abnormalities. They demonstrate the diversity and complexity of signal transduction, including LRIs, subsequent activation of signaling molecules, intracellular signal transmission, and the ultimate activation of effectors. There is also crosstalk and interaction among these signaling pathways, allowing them to influence and regulate each other, forming a complex network to adapt to different physiological and pathological conditions.

#### Spatial distribution

The spatial distribution of CCC components is crucial for the efficiency and specificity of signal transmission. Cells achieve precise signal localization and transmission by restricting the distribution of receptors, enzymes, and other signaling molecules to specific regions within the cell. In many types of cells, specific receptors are localized to particular microdomains of the cell membrane, such as lipid rafts. Lipid rafts are cell membrane regions rich in cholesterol and sphingolipids, capable of aggregating specific signaling proteins, including GPCRs and RTKs.^178,179^ This localization enhances the interaction between signaling molecules, improving the efficiency and specificity of signal transmission. Directed transport allows cells to regulate the activity of signaling molecules within specific temporal and spatial ranges. Certain signaling proteins are transported to the cell poles during specific phases of cell division or concentrated in the leading edge during cell migration, ensuring the correct execution of cell functions. The transmission of signals within neurons depends on the precise release and reception of neurotransmitters, which occur in highly specific spatial locations. Neurotransmitters are stored in synaptic vesicles at the axon terminals, and upon signal arrival, these vesicles fuse with the presynaptic membrane, releasing neurotransmitters into the synaptic cleft. Receptors typically located on the postsynaptic membrane ensure rapid and accurate signal transmission.

The spatial distribution of signaling molecules also involves the localization and transfer of nuclear receptors. Steroid hormone receptors, such as estrogen and androgen receptors, are usually located in the cytoplasm in their inactive state. Upon hormone binding, the receptor–hormone complex moves into the nucleus, directly regulating the transcription of target genes. This transfer from the cytoplasm to the nucleus is a key step in the signal transmission process, affecting changes in gene expression. Furthermore, the spatial distribution of CCC components affects the assembly of signaling complexes. In the Wnt signaling pathway, the stabilization and nuclear transfer of β-catenin depend on the interaction of multiple signaling molecules in specific cellular regions. In the absence of Wnt signals, β-catenin is captured and degraded in the cytoplasm. When the Wnt signaling pathway is activated, proteins such as Axin are recruited to the cell membrane, where they impede the degradation of β-catenin. This preservation enables β-catenin to amass and translocate to the nucleus, influencing gene expression.

#### Other regulatory mechanisms

In addition, CCC signal transduction is regulated by posttranslational modifications, synthesis and degradation of signaling molecules, etc. Phosphorylation is a common posttranslational modification that is crucial for the regulation of signaling pathways. For example, in the EGF signaling pathway, the binding of EGF to its receptor EGFR triggers autophosphorylation of the receptor. This process boosts the receptor’s tyrosine kinase activity, leading to the activation of downstream signaling pathways such as Ras/MAPK, which in turn stimulates cell proliferation and differentiation. The dynamic balance between phosphorylation and dephosphorylation regulates the strength and duration of the signal, affecting the determination of cell fate.

Moreover, ubiquitination is another key posttranslational modification that regulates signal transduction by tagging proteins for degradation, thereby modulating signaling. In the NF-κB signaling pathway, the ubiquitination and subsequent proteasome-dependent degradation of IκBα are critical steps for activating NF-κB.^180^

Furthermore, the synthesis of signaling molecules such as neurotransmitters is essential for the transmission of neural signals. For instance, serotonin is synthesized from tryptophan catalyzed by tryptophan hydroxylase. The amount of serotonin synthesized directly affects the strength of neural signal transmission and psychological states, such as mood and sleep.^181^ The timely degradation of signaling molecules is also crucial to ensure the temporariness of the signal and the restoration of the resting state. For example, acetylcholine is rapidly degraded by acetylcholinesterase, ending its signal transmission at the neuromuscular junction.^182^ This process is vital for the proper relaxation of muscles and the prevention of continuous contraction (spasm). These mechanisms work together to ensure the dynamic regulation of signal transmission and the cell’s adaptability to environmental changes.

## The clinical application and research progress of CCC

**Fig. 3:**
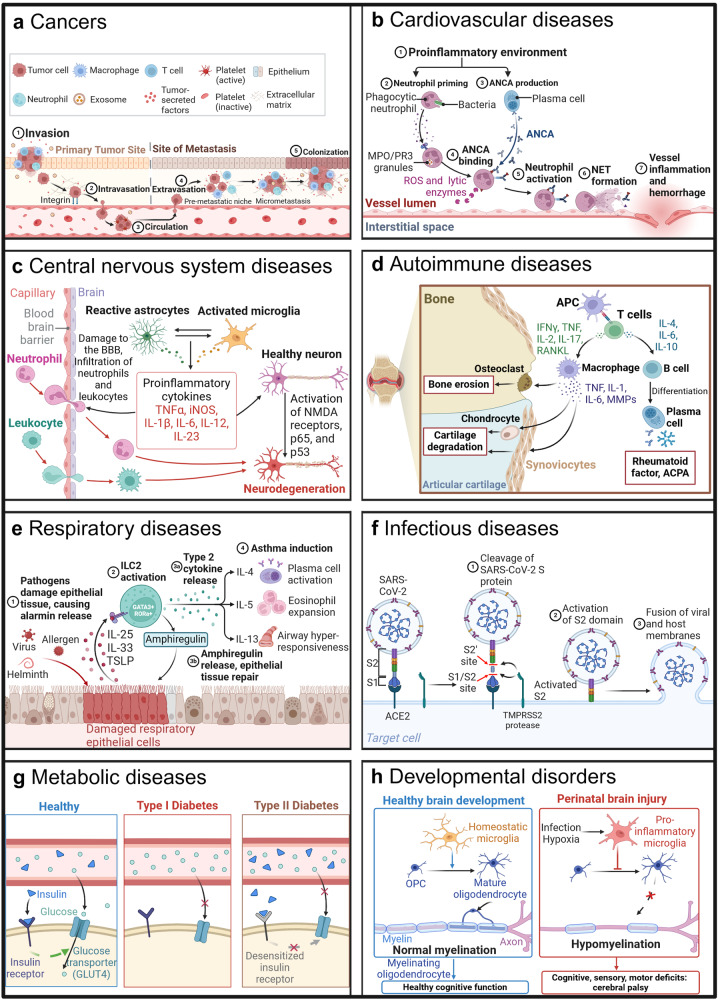
Examples of some diseases caused by representative abnormal CCC. CCC is an essential process that profoundly influences an organism’s homeostasis, development, and disease processes. When cells fail to interact correctly or misinterpret molecular information, diseases typically manifest. **a** Tumor cells invade surrounding tissues and blood vessel walls, infiltrate into blood vessels and spread to other parts of the body along the circulatory system, then interact with original tissue niche cells and migrate to distant tissues to colonize and grow. **b** Antineutrophil cytoplasmic antibody (ANCA)-associated vasculitis: A pro-inflammatory environment initiates the production of ANCA by plasma cells as well as the priming of neutrophils through cytokines. **c** Roles of astrocytes and microglia in neurodegeneration: Danger signals or invading pathogens activate microglia to release pro-inflammatory cytokines, which act on astrocytes, which in turn are activated to release pro-inflammatory cytokines. **d** Pathogenesis of rheumatoid arthritis II: The major cell types and cytokine pathways involved in joint destruction. **e** The role of ILC2s in asthma pathogenesis: Allergens, viruses or helminths provoke the release of alarmins from the damaged epithelium and stimulate the GATA3^+^/RORa^+^ ILC2s to express type 2 cytokines. Interleukins-4, -5, and -13 cause IgE increase from plasma cells, eosinophil expansion, and airway hyper-responsiveness, respectively. **f** Mechanism of SARS-CoV-2 viral entry: SARS-CoV-2 uses its spike (S) protein to adsorb and penetrate cells. S1 binds to the receptor angiotensin-converting enzyme II (ACE2) on the cell membrane through its receptor binding domain (RBD), and S2 mediates the fusion of the viral envelope with the host, allowing the viral nucleocapsid to enter the cytoplasm. **g** Type I vs. type II diabetes: The destruction of the islet cells prevents them from producing insulin, preventing glucose from entering the cells and leading to type 1 diabetes. The reduced responsiveness of the body’s cells to insulin leads to insulin resistance, and the inability to properly use insulin to metabolize glucose results in type 2 diabetes. **h** Differential roles of microglia in the developing brain: During healthy brain development, microglia in its homeostatic state mediates the maturation of oligodendrocyte precursor cells (OPCs) into myelinating mature oligodendrocytes

As the body adapts to internal and external environmental changes, various systems and organs of the body need to coordinate to complete the adaptive response, including nervous, humoral, and self-regulatory systems. At the micro-level, these three regulatory mechanisms rely on the coordinated activities of various functional cells in the body, necessitating a complex signal communication process between different cells, namely cell signal transduction. CCC is so crucial in the development of tissues, organs, and immune responses that diseases can emerge when cells fail to interact correctly or misinterpret molecular information (Fig. 3). Therefore, studying the mechanisms and regulation of cell communication holds great scientific significance for understanding the nature of biology and disease, and has practical application value for clinical trials.

Some pathways may be abnormally activated or inhibited in disease states, and drugs can act by targeting specific cell signaling pathways.^183,184^ However, the use of drugs needs to be strictly controlled to enhance the efficacy of existing treatments and reduce side effects. The United States Food and Drug Administration (FDA) conducts rigorous reviews of medications, and drugs approved by the FDA have passed a series of clinical trials proving their efficacy and safety in treating specific diseases or conditions.^185–188^ Table 1 shows some FDA-approved drugs taking effects through therapeutic CCC targets. In the following sections of this chapter, we will introduce the role of CCC in different human diseases, including disease diagnosis, prevention, treatment, and prediction.

**Table 1:** FDA-approved drugs on various diseases caused by abnormal CCC

FDA-approved drugs on various diseases caused by abnormal CCC				
Generic name of drug	Active ingredients	Initial approval date	Molecular target	Indication
Cancers				
TALZENNA	Talazoparib	03/07/2024	PARP	Metastatic breast cancer
TRUQAP	Capivasertib	11/16/2023	AKT	Breast cancer
AUGTYRO	Repotrectinib	11/15/2023	ROS1, TRKA, TRKB, and TRKC	ROS1-positive non-small cell lung cancer
FRUZAQLA	Fruquintinib	11/08/2023	VEGFR-1, -2, -3	Refractory metastatic colorectal cancer
ZEJULA	Niraparib Tosylate	04/26/2023	PARP-1, -2	Epithelial ovarian, fallopian tube, or primary peritoneal cancer
ORSERDU	Elacestrant	01/27/2023	Erα	Metastatic breast cancer
KRAZATI	Adagrasib	12/12/2022	KRAS G12C	KRAS G12C-mutated locally advanced or metastatic non-small cell lung cancer (NSCLC)
ELAHERE	Mirvetuximab soravtansine-gynx	11/14/2022	FRα	Folate receptor alpha (FRα) positive, platinum-resistant epithelial ovarian, fallopian tube, or primary peritoneal cancer
IMJUDO	Tremelimumab	10/21/2022	CTLA-4	Metastatic non-small cell lung cancer (NSCLC) with no sensitizing epidermal growth factor receptor (EGFR) mutation or anaplastic lymphoma kinase (ALK) genomic tumor aberrations
PLUVICTO	Lutetium lu 177 vipivotide tetraxetan	03/23/2022	PSMA	Prostate-specific membrane antigen (PSMA)-positive metastatic castration-resistant prostate cancer (mCRPC)
OPDUALAG	Nivolumab;Relatlimab-rmbw	03/18/2022	PD-1;LAG-3	Unresectable or metastatic melanoma
RYBREVANT	Amivantamab-vmjw	5/12/2021	EGFR and MET	EGFR exon 20 insertion-mutated non-small cell lung cancer indications
CYTALUX	Pafolacianine	11/29/2021	FR	Ovarian cancer; Known or suspected cancer in the lung
TIVDAK	Tisotumab vedotin-tftv	09/20/2021	TF	Recurrent or metastatic cervical cancer
EXKIVITY	Mobocertinib	09/15/2021	EGFR exon 20 insertion mutations	Locally advanced or metastatic non-small cell lung cancer (NSCLC) with epidermal growth factor receptor (EGFR) exon 20 insertion mutations
JEMPERLI	Dostarlimab-gxly	08/17/2021	PD-1	Endometrial cancer; solid tumors
WELIREG	Belzutifan	08/13/2021	HIF-2α	Von Hippel-Lindau (VHL) dosease; Pancreatic neuroendocrine tumors (pNET)
LUMAKRAS	Sotorasib	05/28/2021	KRAS	KRAS G12C-mutated locally advanced or metastatic non-small cell lung cancer (NSCLC)
TEPMETKO	Tepotinib	02/03/2021	MET	Metastatic non-small cell lung cancer
ORGOVYX	Relugolix	12/18/2020	Pituitary GnRH receptor	Advanced prostate cancer
RIABNI	Rituximab-arrx	12/17/2020	CD20	Non-Hodgkin’s Lymphoma (NHL)
MARGENZA	Margetuximab-cmkb	12/16/2020	HER2	Metastatic HER2-positive breast cancer
GAVRETO	Pralsetinib	09/04/2020	*RET*	Metastatic RET fusion-positive thyroid cancer; Metastatic rearranged during transfection (RET) fusion-positive non-small cell lung cancer
ZEPZELCA	Lurbinectedin	06/15/2020	Guanine residues	Metastatic small cell lung cancer (SCLC)
RETEVMO	Selpercatinib	05/08/2020	RET	Metastatic non-small cell lung cancer (NSCLC); Metastatic medullary thyroid cancer (MTC); Metastatic thyroid cancer; Metastatic solid tumors fusion
TABRECTA	Capmatinib	05/06/2020	MET	Metastatic non-small cell lung cancer (NSCLC)
TRODELVY	Sacituzumab govitecan-hziy	04/22/2020	Trop-2	Metastatic breast cancer; Metastatic urothelial cancer
TUKYSA	Tucatinib	04/17/2020	HER2	Metastatic breast cancer; Unresectable or metastatic colorectal cancer
ENHERTU	Fam-trastuzumab deruxtecan-nxki	12/20/2019	HER2	HER2-positive metastatic breast cancer; HER2-low metastatic breast cancer; Unresectable or metastatic HER2-mutant non-small cell lung cancer
PADCEV	Enfortumab vedotin-ejfv	12/18/2019	Nectin-4	Metastatic urothelial cancer (mUC)
NUBEQA	Darolutamide	07/30/2019	AR	Non-metastatic castration-resistant prostate cancer (nmCRPC); Metastatic hormone-sensitive prostate cancer (mHSPC)
RUXIENCE	Rituximab-pvvr	07/23/2019	CD20	Non-Hodgkin’s lymphoma (NHL)
TRUXIMA	Rituximab-abbs	11/28/2018	CD20	Non-Hodgkin’s lymphoma (NHL)
LORBRENA	Lorlatinib	11/02/2018	ALK and ROS1 as well as TYK1, FER, FPS, TRKA, TRKB, TRKC, FAK, FAK2, and ACK	Metastatic non-small cell lung cancer (NSCLC)
LIBTAYO	Cemiplimab-rwlc	09/28/2018	PD-1	Cutaneous squamous cell carcinoma; Basal cell carcinoma; Non-small cell lung cancer
BRAFTOVI	Encorafenib	06/27/2018	BRAF V600E, as well as wild-type BRAF and CRAF ; JNK1, JNK2, JNK3, LIMK1, LIMK2, MEK4, and STK36	BRAF V600E or V600K mutation-positive unresectable or metastatic melanoma; BRAF V600E mutation-positive metastatic volorectal cancer (CRC); BRAF V600E mutation-positive metastatic non-small cell lung cancer (NSCLC)
VERZENIO	Abemaciclib	02/26/2018	CDK4 and CDK6	Metastatic breast cancer
LYNPARZA	Olaparib	08/17/2017	PARP	Ovarian cancer; Breast cancer; Pancreatic cancer; Prostate cancer
RITUXAN HYCELA	Rituximab; HYALURONIDASE (HUMAN RECOMBINANT)	06/22/2017	CD20	Follicular lymphoma (FL) ; Diffuse large B-Cell lymphoma (DLBCL)
ALUNBRIG	Brigatinib	04/28/2017	ALK, ROS1, insulin-like growth factor-1 receptor (IGF-1R), and FLT-3 as well as EGFR deletion and point mutations	Anaplastic lymphoma kinase (ALK)-positive metastatic non-small cell lung cancer (NSCLC)
RUBRACA	Rucaparib	12/19/2016	PARP	BRCA-mutated recurrent ovarian cancer; BRCA-mutated metastatic castration-resistant prostate cancer
TECENTRIQ	Atezolizumab	10/18/2016	PD-L1	Metastatic non-small cell lung cancer; Locally advanced or metastatic urothelial carcinoma
TAGRISSO	Osimertinib	11/13/2015	EGFR	EGFR mutation-positive non-small cell lung cancer (NSCLC); EGFR mutation-positive metastatic NSCLC
Cardiovascular diseases				
TRYVIO	Aprocitentan	03/19/2024	ET-1	Hypertension
INPEFA	Sotagliflozin	05/26/2023	SGLT2 and SGLT1	Cardiovascular death, hospitalization for heart failure, and urgent heart failure
TAVNEOS	Avacopan	10/07/2021	C5aR	Severe active antineutrophil cytoplasmic autoantibody (ANCA)-associated vasculitis (granulomatosis with polyangiitis [GPA] and microscopic polyangiitis [MPA])
VERQUVO	Vericiguat	01/19/2021	sGC	Cardiovascular death and heart failure (HF) hospitalization
NEXLETOL	Bempedoic acid	02/21/2020	ACL	Primary hyperlipidemia in adults with heterozygous familial hypercholesterolemia (HeFH) or atherosclerotic cardiovascular disease
VYNDAQEL	Tafamidis meglumine	05/03/2019	TTR	Cardiomyopathy of wild-type or hereditary transthyretin-mediated amyloidosis (ATTR-CM)
GIAPREZA	Angiotensin II	12/21/2017	G-protein-coupled angiotensin II receptor type 1	Increase blood pressure
BEVYXXA	Betrixaban	06/23/2017	Fxa	Thromboembolic complications
ENTRESTO	Sacubitril/valsartan	07/07/2015	AT1	Cardiovascular death and hospitalization for heart failure
KENGREAL	Cangrelor	06/22/2015	P2Y12	Periprocedural myocardial infarction (MI), repeat coronary revascularization, and stent thrombosis (ST)
CORLANOR	Ivabradine hydrochloride	04/15/2015	HCN)	Hospitalization for worsening heart failure
SAVAYSA	Edoxaban	01/08/2015	FXa	Stroke and systemic embolism (SE)
Central nervous system diseases				
WAINUA (AUTOINJECTOR)	Eplontersen	12/21/2023	TTR mRNA	The polyneuropathy of hereditary transthyretin-mediated amyloidosis
ZAVZPRET	Zavegepant	03/09/2023	CGRP	Migraine with or without aura
SKYCLARYS	omaveloxolone	02/28/2023	Nrf2	Friedreich’s ataxia
LEQEMBI	Lecanemab-irmb	01/06/2023	Amyloid beta plaques	Alzheimer’s disease
AMVUTTRA	Vutrisiran	06/13/2022	TTR mRNA	The polyneuropathy of hereditary transthyretin-mediated amyloidosis
QULIPTA	Atogepant	09/28/2021	CGRP	Migraine
ADUHELM	Aducanumab-avwa	06/07/2021	Amyloid beta	Alzheimer’s disease
DANYELZA	Naxitamab-gqgk	11/25/2020	GD2	Relapsed or refractory high-risk neuroblastoma in the bone or bone marrow
DETECTNET	Copper Cu 64 dotatate injection	09/03/2020	SSTR2	Positron emission tomography (PET) for localization of somatostatin receptor positive neuroendocrine tumors (NETs)
ENSPRYNG	Satralizumab-mwge	08/14/2020	IL-6	Neuromyelitis optica spectrum disorder (NMOSD)
EVRYSDI	Risdiplam	08/07/2020	SMN2	Spinal muscular atrophy (SMA)
UPLIZNA	Inebilizumab-cdon	06/11/2020	CD19	Neuromyelitis optica spectrum disorder (NMOSD)
TAUVID	Flortaucipir F18	05/28/2020	Aggregated tau protein	Alzheimer’s disease (AD)
ONGENTYS	Opicapone	04/24/2020	COMT	Parkinson’s disease (PD)
KOSELUGO	Selumetinib	04/10/2020	MEK1/2	Neurofibromatosis type 1 (NF1)
NURTEC ODT	Rimegepant	02/27/2020	Calcitonin gene-related peptide receptor	Migraine
VYEPTI	Eptinezumab-jjmr	02/21/2020	CGRP	Migraine
UBRELVY	Ubrogepant	12/23/2019	Calcitonin gene-related peptide receptor	Migraine
REYVOW	Lasmiditan	10/11/2019	5-HT1F	Migraine
NOURIANZ	Istradefylline	08/27/2019	A2A	Parkinson’s disease (PD)
TEGSEDI	Inotersen	10/05/2018	TTR mRNA	Polyneuropathy of hereditary transthyretin-mediated amyloidosis
AJOVY	Fremanezumab-vfrm	09/14/2018	CGRP	Migraine
AIMOVIG	Erenumab-aooe	05/17/2018	CGRP	Migraine
BRINEURA	Cerliponase alfa	04/27/2017	M6P/IGF2	Infantile neuronal ceroid lipofuscinosis type 2 (CLN2)
INGREZZA	Valbenazine	04/11/2017	VMAT2	Huntington’s disease; Tardive dyskinesia
AUSTEDO	Deutetrabenazine	04/03/2017	VMAT2	Huntington’s disease; Tardive dyskinesia
XADAGO	Safinamide	03/21/2017	MAO-B	Parkinson’s disease (PD)
SPINRAZA	Nusinersen	12/23/2016	SMN	Spinal muscular atrophy (SMA)
NUPLAZID	Pimavanserin	04/29/2016	serotonin 5-HT2A	Parkinson’s disease
BRIVIACT	Brivaracetam	02/18/2016	SV2A	Partial-onset seizures
UNITUXIN	Dinutuximab	03/10/2015	GD2	High-risk neuroblastoma
Autoimmune diseases				
BIMZELX	Bimekizumab	10/17/2023	IL-17A, IL-17F, and interleukin 17-AF cytokines	Moderate-to-severe plaque psoriasis
BRIUMVI	Ublituximab-xiiy	12/28/2022	CD20	Relapsing forms of multiple sclerosis (MS)
SOTYKTU	Deucravacitinib	09/09/2022	TYK2	Moderate-to-severe plaque psoriasis
SPEVIGO	Spesolimab-sbzo	09/01/2022	IL-36	Generalized pustular psoriasis (GPP)
VTAMA	Tapinarof	05/23/2022	AhR	Plaque psoriasis
SAPHNELO	Anifrolumab-fnia	07/30/2021	IFN	Moderate-to-severe systemic lupus erythematosus (SLE)
PONVORY	Ponesimod	03/18/2021	S1P	Multiple sclerosis (MS)
ZEPOSIA	Ozanimod	03/25/2020	S1P	Multiple sclerosis (MS); Ulcerative colitis (UC)
RINVOQ	Upadacitinib	08/16/2019	JAK	Rheumatoid arthritis; Psoriatic arthritis; Atopic dermatitis
MAYZENT	Siponimod	03/26/2019	S1P	Multiple sclerosis (MS)
CABLIVI	Caplacizumab-yhdp	02/06/2019	A1-domain of vWF	Acquired thrombotic thrombocytopenic purpura (aTTP)
OLUMIANT	Baricitinib	05/31/2018	JAK	Moderately to severely active rheumatoid arthritis
TREMFYA	Guselkumab	07/13/2017	IL-23	Moderate-to-severe plaque psoriasis
KEVZARA	Sarilumab	05/22/2017	IL-6	Rheumatoid arthritis
OCREVUS	Ocrelizumab	03/28/2017	CD20	Multiple sclerosis (MS)
ZINBRYTA	Daclizumab	05/27/2016	IL-2	Multiple sclerosis (MS)
TALTZ	Ixekizumab	03/22/2016	IL-17A	Moderate-to-severe plaque psoriasis; Psoriatic arthritis; Ankylosing spondylitis; Active non-radiographic axial spondyloarthritis
COSENTYX	Secukinumab	01/21/2015	IL-17A	Moderate to severe plaque psoriasis
Respiratory diseases				
BEYFORTUS	Nirsevimab-alip	07/17/2023	RSV	RSV lower respiratory tract disease
PAXLOVID (COPACKAGED)	Nirmatrelvir, ritonavir	05/25/2023	SARS-CoV-2; CYP3A	COVID-19
TEZSPIRE	Tezepelumab-ekko	12/17/2021	TSLP	Severe asthma
VEKLURY	Remdesivir	10/22/2020	SARS-CoV-2	COVID-19
XENLETA	Lefamulin	08/19/2019	The A- and P-sites of the peptidyl transferase center (PTC) in domain V of the 23 s rRNA of the 50 S subunit	Community-acquired bacterial pneumonia (CABP)
PRETOMANID	Pretomanid	08/14/2019	Mycolic acid	Pulmonary tuberculosis (TB)
AEMCOLO	Rifamycin	11/9/2018	The beta subunit of the bacterial DNA-dependent RNA polymerase	Travelers’ diarrhea
FASENRA	Benralizumab	11/14/2017	IL-5Rα	Severe asthma
CINQAIR	Reslizumab	03/23/2016	IL-5	Severe asthma
NUCALA	Mepolizumab	11/04/2015	IL-5	Severe asthma; Chronic rhinosinusitis
Infectious diseases				
PAXLOVID (COPACKAGED)	Nirmatrelvir, ritonavir	05/25/2023	SARS-CoV-2; CYP3A	COVID-19
SUNLENCA	Lenacapavir sodium	12/22/2022	p24	HIV-1
LIVTENCITY	Maribavir	11/23/2021	CMV	Post-transplant CMV infection/disease
CABENUVA KIT	Cabotegravir and rilpivirine (copackaged)	01/21/2021	Integrase active site; HIV-1 reverse transcriptase (RT)	HIV-1
EBANGA	Ansuvimab-zykl	12/21/2020	Glycan cap and inner chalice of the EBOV GP1 subunit	*Zaire ebolavirus*
VEKLURY	Remdesivir	10/22/2020	SARS-CoV-2 RNA-dependent RNA polymerase (RdRp)	COVID-19
INMAZEB	Atoltivimab, maftivimab, and odesivimab-ebgn	10/14/2020	*Zaire ebolavirus* glycoprotein (GP)	*Zaire ebolavirus*
RUKOBIA	Fostemsavir	07/02/2020	Gp120 subunit within the HIV-1 envelope glycoprotein gp160	HIV-1
PIFELTRO	Doravirine	08/30/2018	HIV-1 reverse transcriptase (RT)	*HIV-1*
TPOXX	Tecovirimat	07/13/2018	Orthopoxvirus VP37 protein	Human smallpox disease
TROGARZO	Ibalizumab-uiyk	03/06/2018	Domain 2 of CD4	HIV-1
MAVYRET	Glecaprevir and pibrentasvir	08/03/2017	HCV NS3/4 A protease	HCV
VOSEVI	Sofosbuvir, velpatasvir and voxilaprevir	07/18/2017	HCV NS5B RNA-dependent RNA polymerase	HCV
ZEPATIER	Elbasvir and grazoprevi	01/28/2016	HCV NS5A; HCV NS3/4 A protease	HCV
DAKLINZA	Daclatasvir	07/24/2015	NS5A	HCV
Metabolic diseases				
RIVFLOZA	Nedosiran	09/29/2023	GalNAc aminosugar residues	Primary hyperoxaluria type 1 (PH1)
POMBILITI	Cipaglucosidase alfa-atga	09/28/2023	M6P	Late-onset Pompe disease
BRENZAVVY	Bexagliflozin	01/20/2023	SGLT2	Type 2 diabetes
TZIELD	Teplizumab-mzwv	11/17/2022	CD3	Type 1 diabetes (T1D)
MOUNJARO	Tirzepatide	05/13/2022	GIP and GLP-1	Type 2 diabetes
LEQVIO	Inclisiran	12/22/2021	GalNAc	Primary hyperlipidemia
NEXVIAZYME	Avalglucosidase alfa-ngpt	08/06/2021	M6P	Late-onset pompe disease
KERENDIA	Finerenone	07/09/2021	MR	Type 2 diabetes (T2D)
ZEGALOGUE	Dasiglucagon	03/22/2021	Hepatic glucagon receptors	Severe hypoglycemia
OXLUMO	Lumasiran	11/23/2020	Hydroxyacid oxidase 1 (*HAO1*)	Primary hyperoxaluria type 1 (PH1)
LOKELMA	Sodium zirconium cyclosilicate	05/18/2018	Potassium	Hyperkalemia
STEGLATRO	Ertugliflozin	12/19/2017	SGLT2	Type 2 diabetes
OZEMPIC	Semaglutide	12/05/2017	GLP-1	Type 2 diabetes
MEPSEVII	Vestronidase alfa-vjbk	11/15/2017	Lysosomes	Mucopolysaccharidosis VII
ADLYXIN	Lixisenatide	07/27/2016	GLP-1	Type 2 diabetes
ZURAMPIC	Lesinurad	12/22/2015	URAT1; OAT4	Hyperuricemia
VELTASSA	Patiromer for oral suspension	10/21/2015	Potassium	Hyperkalemia
TRESIBA	Insulin degludec injection	09/25/2015	Circulating albumin	Diabetes mellitus
Developmental disorders				
DUVYZAT	Givinostat	03/21/2024	Histone deacetylase	Duchenne muscular dystrophy (DMD)
AGAMREE	Vamorolone	10/26/2023	Glucocorticoid receptor	Duchenne muscular dystrophy (DMD)
NGENLA	Somatrogon-ghla	06/27/2023	GH	Growth failure due to inadequate secretion of endogenous growth hormone
VOXZOGO	Vosoritide	11/19/2021	NPR-B	Achondroplasia with open epiphyses
SKYTROFA	Lonapegsomatropin-tcgd	08/25/2021	GH	Growth failure due to inadequate secretion of endogenous growth hormone (GH)
AMONDYS 45	Casimersen	02/25/2021	Exon 45 of dystrophin pre-mRNA	Duchenne muscular dystrophy (DMD)
SOGROYA	Somapacitan-beco	08/28/2020	Dimeric GH receptor	Growth failure due to inadequate secretion of endogenous growth hormone (GH)
VILTEPSO	Viltolarsen	08/12/2020	Exon 53 of dystrophin pre-mRNA	Duchenne muscular dystrophy (DMD)
VYONDYS 53	Golodirsen	12/12/2019	Exon 53 of dystrophin pre-mRNA	Duchenne muscular dystrophy (DMD)
MACRILEN	Macimorelin acetate	12/20/2017	Growth hormone secretagogue receptors	Adult growth hormone deficiency
EMFLAZA	Deflazacort	02/09/2017	Glucocorticoid receptor	Duchenne muscular dystrophy (DMD)
EXONDYS 51	Eteplirsen	09/19/2016	Exon 51 of dystrophin pre-mRNA	Duchenne muscular dystrophy (DMD)

The FDA-approved drugs summarized from 2015 to the present day. These drugs are mainly used to treat cancers, cardiovascular diseases, central nervous system diseases, respiratory diseases, infectious diseases, metabolic diseases, and developmental disorders

### Cancers

Cancer is widely recognized as a cluster of disorders marked by uncontrolled proliferation and dissemination of aberrant cells.^189^ Cancer remains a major global challenge even though significant efforts have been made to develop new cancer treatments. As a result, the discovery of novel therapeutics specifically targeting diverse cancer forms is imperative.^190^ Since various signal transduction pathways regulate cell growth, abnormal activation or suppression of these pathways drives tumorigenesis.^121^ One of the principal pathways is the PI3K–AKT–mTOR signaling that linked to drug resistance and the malignant tumor process in solid cancer patients.^191^ It is vital to use cell signaling molecules to recognize cancer cells to inhibit the expansion and proliferation of cancer cells. Posttranslational protein modification plays a vital role in the control of cellular signaling. Diverse protein kinases and phosphatases regulate the phosphorylation and dephosphorylation of proteins. Tumors frequently exhibit irregular or uncontrolled activation of such kinases and phosphatases, making them as essential targets for targeted cancer therapies. To cite an instance, Imatinib is a BCR–ABL fusion tyrosine kinase inhibitor and represents the first kinase inhibitor successfully applied in treating chronic myeloid leukemia (CML).^192^ Subsequently, inhibitors targeting protein kinases such as mTOR, VEGFR, MAPK, EGFR, CDK12, and ERBB2 have been employed in treating various common malignant tumors.^193–197^ Employing gene-editing technologies like CRISPR/Cas9 to intervene at the genetic level in cancer cells, whether by knocking out or modifying specific genes, holds the promise of hindering cancer cell proliferation and metastasis.^198,199^

While genetic or epigenetic alterations are often cited as the root cause of cancer, the progression of cancer is intricately linked with crosstalk among tumor cells, surrounding stromal cells, and the extracellular matrix (ECM).^200^ Tumor cells promote their own growth and proliferation by communicating with surrounding normal cells, immune cells, and other cell types within the tumor microenvironment (TME), such as fibroblasts and endothelial cells. Cancer cells do not manifest the disease in isolation but rather conscript and corrupt resident and recruited normal cell types.^201^ Cancer cells can select noncancerous cells to engage in extensive chemical and physical interactions, with many types of cells being recruited into solid tumors and participating in complex interactions that enable cancer cells to invade.^202^ Tumor invasion is not a simple autonomous process of cancer cells but relies on a complex network of paracrine interactions.^203^ Moreover, this network can change as cancer cells disseminate. As the constituent cells of blood vessels and lymphatic vessels, endothelial cells not only supply tumor with nutrition and oxygen but also act as an “escape route” for cancer cells, enabling them to metastasize to distant sites.^204^ The intercellular communication between these TME components and cells is a driver of cancer progression and significantly impacts the efficacy of therapeutic interventions.

The study of the TME involves cell communication analysis to select the most interacting cell subsets and further investigate their mechanisms. For example, by using receptor-ligand analysis of different subpopulations in bladder cancer samples, one study showed that inflammatory cancer-associated fibroblasts (iCAFs) specially interact with endothelial cells to promote angiogenesis and tumor proliferation, revealing the role of iCAFs in the immune microenvironment of bladder cancer.^205^ In addition, researchers found the specific expression of ACKR1 in tumor endothelial cells is associated with unfavorable prognostic outcomes in a gastric cancer cohort, providing a new target for treating gastric cancer.^206^ Various characteristics of tumors are primarily regulated by the TME, including dysregulated ECM, sustained activation of proliferative signals, inhibition of suppressors and apoptosis, activated invasion and metastasis, metabolic dysregulation, and evasion of immune destruction. Furthermore, factors secreted by the primary tumor can modify the microenvironment of distant organs, rendering them conducive to subsequently colonized by metastatic cancer cells.^207^ The growth and progression of tumors depend on angiogenesis, with CAFs being a primary source of pro-angiogenic factors such as VEGF or PDGF.^208,209^ Tumor cells discharge pro-angiogenic factors into their surrounding environment, contributing to the secretion of PDGF by endothelial cells, which attracts supporting cells to solidify the nascent blood vessels.^210^ The PDGF released by tumor cells directly binds to receptors on recruited bone marrow progenitor cells and induces differentiation into endothelial cells or SMCs through signal activation, promoting their growth and migration.^211,212^

Mutations in genes and their respective signaling pathways are the primary consequences leading to cell apoptosis, proliferation, cell survival, and differentiation.^213,214^ A significant number of genes frequently mutated in cancer are responsible for encoding components or targets of the PI3K–Akt and Ras-ERK pathways. Typically, these pathways are transiently activated in response to signals from growth factors or cytokines and the occupancy of ligands for integrin adhesion receptors. Subsequently, mutations in the tumor suppressor genes TSC1 and TSC2 lead to the overactivation of mTORC1 signaling, an important target of PI3K–Akt signaling.^215^ The transcription factor Myc is a significant downstream target of the Ras-ERK signal as well as numerous other pathways, and it is often amplified or overexpressed in cancer. Furthermore, the tumor microbiome may also emerge as a critical factor in shaping the local immune response in the TME.^216^ They can enhance anti-tumor immunity through mechanisms such as stimulator of interferon genes signaling activation, T and natural killer (NK) cell activation, tertiary lymphoid structure production, and presentation of tumor microbiome-derived antigens. In addition, they can reduce anti-tumor immune responses and promote cancer progression by increasing reactive oxygen species (ROS) levels, fostering an anti-inflammatory milieu, deactivating T cells, and inducing immunosuppression.^217,218^ Immune checkpoint blocking (ICB) is a revolutionary cancer treatment that blocks the interaction of inhibitory molecules expressed on malignant cells with T cells, rejuvenating T cells in the early stages of dysfunction. The main types of ICB therapy include PD-1/PD-L1 inhibitors and CTLA-4 inhibitors. PD-L1 (the ligand of PD-1) is mainly expressed on tumor cells and tumor-infiltrating immune cells, and it functions to inhibit T-cell activity by binding to PD-1.^219,220^ The action of PD-1/PD-L1 inhibitors is to block the binding between PD-1 and PD-L1, restoring the immune cells’ ability to recognize and kill tumor cells.^221^ CTLA-4 inhibitors work by blocking the binding of CTLA-4 to the B7 molecules (B7-1 and B7-2) on the surface of antigen-presenting cells, thereby relieving the inhibitory state of T cells. In addition, CTLA-4 inhibitors can also reduce the number of regulatory T cells (Tregs) that highly express CTLA-4 in the tumor microenvironment by blocking CTLA-4, thereby relieving the immunosuppressive effects of Treg cells and promoting the activation and proliferation of effector T cells.^222–225^ These two types of inhibitors target different immune checkpoint proteins, but their mechanisms of action are similar, both working by blocking immune checkpoint proteins to activate the immune system. Recent studies have identified IGSF8 as an innate immune checkpoint and tumor immunotherapeutic target.^226^ The newly developed IGSF8.06 antibody can block the inhibitory effect of *IGSF8* expressed on tumors on NK cell function, thus stimulating NK cells to kill malignant cells with antigen presentation defects and stress signals.^226^

Stem cells, a vital component of cell therapy, play a crucial role in restoring organs and tissues, holding immense promise for various applications. It should be noted that stem cells derived from different sources exhibit varying capabilities in terms of proliferation, migration, and differentiation. These differences influence their suitability for deployment in anti-tumor therapy. In detail, cancer stem cells (CSCs) represent a small fraction of cancerous cells characterized by their capacity for multifaceted differentiation, high self-renewal, and tumorigenicity.^227^ The CSC theory postulates the existence of a minor yet crucial cadre of self-perpetuating cancer cells critical in tumor metastasis, recurrence, and resistance to treatment.^227^ Nevertheless, the precision and biological role of CSCs are still ambiguous, prompting some researchers to exercise caution and regard the theory as contentious.^228,229^ Despite the ongoing debate, research on CSCs continues to evolve and uncover new insights.^230^ CSCs were originally extracted from cases of acute myeloid leukemia,^231,232^ possibly emerging from regular tissue-specific stem cells or differentiated cells at the onset of the tumor, triggering survival pathways and perpetual proliferation.^233^ Mechanistic studies suggest dysfunction in some developmental and homeostasis signaling pathways could facilitate uncontrolled self-renewal and differentiation essential for CSC functionality.^234^ Such molecular signaling pathways, including Notch,^17^ Hedgehog,^235^ Wnt/β-catenin,^236^ PI3K/PTEN,^237^ JAK/STAT,^238^ and NF-κB,^239^ are known to regulate normal stem cell proliferation. Further changes in these signaling pathways will lead to the formation of CSCs and subsequent cancer cells. Given that, biomarkers of CSC are instrumental in diagnosing cancer, guiding targeted treatments, and forecasting disease progression since growing evidences indicate CSCs may play pivotal roles in critical disease stages from cancer initiation to metastatic spread.^240^

### Cardiovascular diseases

Vital body functions such as heartbeat and blood pressure maintenance are under the control of the autonomic nervous system. The cardiovascular system respond to sympathetic stimulation of hormones secreted from nerve terminals by adrenergic receptors (ARs), which are the dominant GPCRs in the heart. In vascular smooth muscle, catecholamine stimulation causes vasoconstriction through α-ARs and causes vasodilation through β_2_-ARs. In the heart, catecholamine stimulation causes increased heart rate and myocardial contractility through β-AR. The signaling pathway most extensively researched in cardiac myocytes is activated in response to β-adrenergic stimulation.^241,242^

The contraction of the heart is initiated by an elevation in cytosolic Ca^2+^ concentration within cardiac myocytes following their electrical activation. This process is regulated by a multitude of signaling pathways, which involve cascades of signaling molecules culminating in posttranslational modification (PTM, e.g., phosphorylation) of target proteins.^243^ For example, CaMKII is a pivotal regulator of excitation-contraction coupling and Ca^2+^ cycling, in charge of numerous essential cardiac functions. According to reports, the expression level and activity of the main cardiac subtype CaMKIIδ are unregulated in human heart failure.^244,245^ Chronic overactivation of CaMKII can lead to several other pathological symptoms, including cardiac hypertrophy,^246^ diastolic and systolic dysfunction,^247,248^ arrhythmia,^249,250^, and ischemia/reperfusion injury.^251,252^ Different PTMs of CaMKII lead heart disease through different pathological mechanisms. In details, oxidized CaMKII contributes to apoptosis post-myocardial infarction and atrial fibrillation,^246^ while O-GlcNAcylation contributes to hyperglycemia-induced arrhythmia.^253–256^ Whereas, nitrosylation of CaMKII confers a sex-dependent protective effect against harm from ischemia/reperfusion in females.^257^

Cardiovascular disease encompasses a range of conditions impacting the heart or circulatory system, including heart failure, coronary artery disease, stroke, high blood pressure, and atherosclerosis. Atherosclerosis is a chronic inflammatory disease characterized by the formation of lipid-rich plaques on the walls of blood vessels, which can lead to myocardial infarction, stroke, unstable angina, and sudden cardiac death.^258–260^ Atherosclerosis is not considered simply as a lipid storage disorder any more, as research has reported the involvement of inflammatory mechanisms in the progression of the disease, such as the accumulation of leukocytes at site of lesion.^261,262^ Leukocytes within the plaque produce growth factors, inducing SMC proliferation in advanced lesions.^263^ The flow of atherosclerosis triggers NF-κB activation in endothelial cells, leading to the production of inflammatory cytokines, thereby establishing an environment conducive to atherosclerosis.^264^ A typical atherosclerotic plaque contains a lipid core, with apoptotic macrophages constituting a necrotic core.^265^ Macrophage activation triggers the release of various cytokines, transformation into foam cells, and subsequent necrosis.^266^

In addition, coronary heart disease (CHD) accounts for 42.1% of all cardiovascular disease deaths. High-density lipoprotein-associated cholesterol (HDL-C) is linked to lower risk and enhanced outcomes in CHD patients via CCC. Cholesterol is transported from peripheral tissue cells such as macrophages or vascular SMCs to the liver through HDL-C for recovery or excretion through bile or feces.^267–269^ ApoA-1 is the major HDL structural protein, which has been recognized as an anti-atherosclerotic marker for acquiring cholesterol and phospholipids effluxed by hepatocytes and enterocytes.^270^ The deposition of cholesterol in arteries can initiate the atherosclerotic process, giving rise to the infiltration of multifarious cell types including macrophages, fibroblasts, and SMCs, all of which play a role in plaque formation.^267^

Studies have documented the presence of extracellular vesicles (EVs) within developing plaques and intimal lesions of advanced plaques, indicating their role at both the inception and culmination of plaque formation in humans.^271–273^ EVs originating from foam cells have been identified as catalysts for SMC migration and activation of the ERK pathway, thereby exacerbating lesion progression.^274^ Research has shown that following exposure to an atherogenic trigger like oxidized low-density lipoprotein, macrophage EVs are enriched with numerous miRNAs, including miR-146a, miR-128, and miR-185.^275^ Furthermore, miR-146a has been implicated in accelerating atherosclerosis progression through the promotion of macrophage migration towards the vascular wall.^275^ Intercellular communication between endothelial cells and SMCs is crucial for maintaining vascular homeostasis. The transfer of miR-155 mediated by EVs from SMCs to endothelial cells, driven by KLF5, leads to the disruption of tight junctions and endothelial barrier integrity, promoting atherosclerosis.^276^ The transfer of miR-143 and miR-145 in endothelial cell EVs induced by KLF2 blocks the transdifferentiation of SMCs, thereby mediating a protective effect against atherosclerosis through endothelial cell-SMC communication.^277^

In the heart, increasing evidence suggests the presence of CCC among cardiomyocytes and non-myocyte cells such as fibroblasts and macrophages. Besides, cardiomyocytes and vascular endothelial cells share numerous systems of CCC, including direct communication and paracrine signals such as pansexins, hemichannels, and purinergic signals. It is reasonable to believe that they can regulate each other’s behavior through CCC. For example, vascular dysfunction is related to arrhythmia.^278–280^ With our increasing understanding of CCC, new opportunities will emerge to promote the treatment of various cardiovascular diseases.

### Central nervous system diseases

Almost one-sixth of the people in the world suffer from central nervous system (CNS) diseases, ranging from mild nerve injury to coma and even brain death.^281^ The main cell type in neural tissue is neurons. The primary function of neurons is to be capable of communicating with each other and with other cell types. The axons of neurons release contents to synaptic intervals through exocytosis, transmitting these chemical neurotransmitters to receptors on another postsynaptic cell.^282^

Exosomes are active participants in CCC, being released by a variety of cell types within the body, including neurons. Present in diverse body fluids like blood, cerebrospinal fluid, alveolar lavage fluid, ascites, and amniotic fluid, exosomes influence other cells, triggering a range of physiological or pathological responses.^283^ For example, exosomes secreted by oligodendroglioma cells can induce neuronal death. In conditions such as amyotrophic lateral sclerosis (ALS), frontotemporal dementia (FTD), FTD-ALS, tau protein disease, Parkinson’s disease (PD), and Alzheimer’s disease (AD), exosomes migrate through the blood and cerebrospinal fluid, carrying misfolded proteins or pro-inflammatory molecules.^284–286^ Exosomes released by neurons can be internalized by other neurons, indicating a novel avenue for interneuronal communication.^287^ Neurons in the CNS secrete exosomes to orchestrate intricate communication with astrocytes and microglia, facilitating extensive crosstalk that governs neuronal regeneration and synaptic function throughout both developmental stages and adult life.^288,289^ In their role of regulating microenvironment, astrocytes and oligodendrocytes produce EVs to enhance intercellular communication and the activity of target cells.^290–292^ The release of exosomes is speculated as a critical process in neurogenesis, essential for protein clearance, and is triggered by the fusion of late endosomes and lysosomes during axonal elongation.^293–295^

In the CNS, exosomes potentially play a dual role: they are vital components essential for the CNS development and protection under normal conditions; while their participation in the pathogenesis might worsen the conditions of certain neurodegeneration and neuroinflammation.^296–298^ For instance, elevated levels of microglial exosomes have been observed in Alzheimer’s disease patients, and exosomes from oligodendrocytes have been implicated in inducing neuronal death.^299^ The extensive interactions between glial-derived exosomes and neurons also suggest that these vesicles are instrumental in both the formation and sustenance of neural circuits, evidenced by their promotion of neurite outgrowth in hippocampal neurons and enhancement of cortical neuron viability.^292^ Moreover, exosomes originating from microglia are known to regulate the activity of neuron by enhancing myelin metabolism.^290^ The ability of exosomes to access the bloodstream and cerebrospinal fluid renders these vesicles potential means for remote communication and transportation, facilitating the delivery of bioactive molecules to specific targets.^300^ As exosomes are capable of traversing the blood–brain barrier (BBB) and preserving the characteristics of their originating cells, circulating exosomes can offer insights into the condition of the originating tissue.^301,302^ This feature presents a precise and minimally invasive approach (via peripheral blood sampling) for the early diagnosis of neurological disorders.^303–305^. In this chapter, we focus on the relationship between CNS and exosomes, and review how exosomes affect CCC to lead to CNS diseases. Consequently, various strategies are outlined for diagnosing and treating CNS conditions by leveraging exosomes in the realm of CCC.

Alpha-synuclein (α-syn) plays a central role in the pathogenesis of PD, and its elevated levels are adequate to cause PD.^306–309^ Exosomes in the blood carry α-syn and intensifies the accumulation and aggregation of α-syn through various mechanisms such as miRNAs, consequently triggering inflammation, inhibiting autophagy, and contributing to the pathogenesis of Parkinson’s disease.^306,308,310–317^ Exosomes derived from glial cells transport α-syn and inflammatory factors from glial cells to neurons, exacerbating the progression of PD.^318^ Contents of exosomes derived from various bodily fluids can serve as biomarkers for diagnosing PD. For example, exosomes in the plasma of PD patients were found an elevation expression of α-syn and tau proteins. The presence of α-syn includes additional characteristics such as β-sheet-rich structures and a fibrillary appearance, indicating the pathological transformation of this protein.^308,319,320^ As for the miRNA, the expression of miR-128, miR-505, and miR-19b is downregulated, while the expression of miR-331-5p, miR-24, and miR-195 is increased in the patient’s blood exosomes.^321–323^ Elevated phosphorylation levels of leucine-rich repeat kinase 2, as well as increased levels of synaptosome-associated protein 23 and calbindin proteins linked to PD-related damage, were identified in exosomes isolated from the urine of individuals with PD.^324,325^ In addition, leucine-rich repeat kinase 2 and α-syn as a biomarker has entered the clinical stage.^326^

AD is the most common form of dementia, of which the number will reach 130 million by 2050.^327^ Due to the accumulation of exosomes proteins in amyloid plaques in the brain of AD patients, exosomes are receiving increasing attention. Exosomes derived from different cell types play different functions in AD. High concentrations of microglial exosomes were found in AD patients.^299^ Exosomes derived from neuronal cells contain precursors of amyloid protein and enzymes used for precursor maturation. Plasma exosomes accumulate in amyloid plaques and participate in plaque formation.^328^ Exosomes derived from M1 microglia stimulate activation of resting microglia and enhance pro-angiogenic responses via Irf1/miR-155-5p/Socs1 axis in the retina.^329^ Exosomes spring from M2 microglia mitigate neuronal damage and mitochondrial dysfunction in AD through the PINK1/Parkin pathway.^330^ MicroRNAs that affect the occurrence and development of AD are present in exosomes derived from both peripheral blood and cerebrospinal fluid in patients with AD.^331^ For example, exosomes affect the progression of AD by blocking the transcription of amyloid precursor protein (APP) through miR-185-5p or exosome content miR-193b.^332,333^ In contrast, exosomes come from human cerebral spinal fluid or N2a cells enhance the synaptic plasticity destruction activity of synthesis and AD brain-derived amyloid-β (Aβ) in vivo.^334^ In addition, exosomes from astrocytes with accumulated cholesterol significantly contribute to the transport of APP/Aβ peptides and the influence of neuronal viability in the affected AD brain regions.^335^

The excessive phosphorylation of tau protein is also a characteristic of AD. Exosomes secreted by microglia are involved in the transport of tau protein. Inhibiting the synthesis or secretion of exosomes prevents the aggregation of tau protein in the brain.^336–338^ Inhibition of exosome biosynthesis by blocking the activity of a key enzyme regulating ceramide biosynthesis, neutral sphingomyelinase 2, reduced the proliferation of Aβ plaque and tau in AD mouse model.^339^ Some enzymes carried in exosomes, such as neprilysin and insulin-degrading enzymes, reduce intracellular and extracellular Aβ levels.^340^ In summary, exosomes participate in the pathogenesis of AD by transmitting different substances or information. Therefore, exosomes serve as transmission factors and diagnostic biomarkers for AD.^341,342^

In the exploration of AD diagnosis, some scientists believe that exosomes in the blood of AD patients are fewer and smaller.^343^ while there are also reports that exosomes in AD patients are bigger.^344^ Brain-derived exosomes in AD patients exhibited significant alterations in glycerophospholipid and sphingolipid levels, especially an elevated level of plasmalogen glycerophosphoethanolamine and a reduction in polyunsaturated fatty acyl-containing lipids.^345^ More than 20 exosomal miRNAs in AD patients were found to be significantly different from those in control group by using next-generation sequencing (NGS).^346–348^ The contents of these exosomes may have high potential value in the diagnosis of AD.

In recent years, the roles of mast and microglia in the nervous system have also been discovered. After responding to environmental signals, mast cells secrete different neurotransmitters or neurotrophic factors. This paracrine secretion leads to acute activation and/or long-lasting changes in excitability and phenotype, which is associated with neuroinflammation.^349^ Besides, exosomes derived from glial cells bind to toll-like receptor 2 and the toll-like receptor 4 of neurons, leading to neuroinflammation and even neuronal apoptosis. Exosomes derived from glial cells transport abnormally expressed miRNAs, triggering and spreading neuroinflammation.^311,315^ Understanding neuroinflammation also requires recognizing that the non-neuronal cell–cell interactions between glial cells, mast cells, and the glial cells themselves are integral components of the inflammatory process. In this context, mast cells play a crucial role in orchestrating the inflammatory process, from its initiation to prolonged neuroinflammation.^350^

BBB composed of endothelial cells connected by tight junctions and adherent processes protects potential intruders under physiological conditions. In the treatment of CNS diseases, BBB is an obstacle for drug delivery.^351–353^ In the past few decades, researchers have continuously explored methods for delivering drugs to the brain through BBB. Exosomes have loading and delivery functions, and their lipid bilayer can fuse with membrane-like structures in the body such as the BBB. Being able to pass through BBB and carry goods, exosomes have become a star substance for treating CNS diseases. The first treatment approach involves utilizing exosomes released by specially treated cells, such as exosomes obtained from cells treated with curcumin. These exosomes have shown promise in preventing neuronal death both in vitro and in vivo, alleviating Alzheimer’s disease symptoms by inhibiting tau protein phosphorylation through the activation of the AKT/GSK-3β pathway.^354^ The second treatment avenue involves investigating therapies using exosomes sourced from stem cells, such as exosomes released by human umbilical cord-derived mesenchymal stem cells (MSCs) and adipose-derived mesenchymal stem cells. These exosomes are being explored for their potential to address conditions like neuroinflammation, Alzheimer’s disease, brain injury, and neurodegenerative disorders by reducing Aβ aggregation.^355–357^ Exosomes derived from stem cells sourced from the dental pulp of shed human deciduous teeth exhibited a neuroprotective impact on dopaminergic neurons. Through intranasal delivery, these exosomes demonstrated an improvement in motor function and a reduction in dopaminergic neuron loss in Parkinson’s disease.^326,358^ The third approach involves utilizing exosomes as vehicles to transport various therapeutic agents, including siRNA and other medicinal RNAs, peptides, dopamine, synthetic drugs, bioactive compounds from plants, enzymes, proteins, and antisense oligonucleotides designed to target the human α-synuclein sequence with suitable modifications. This strategy aims to treat central nervous system diseases by leveraging exosomes as carriers for delivering these therapeutic payloads.^359–367^

### Autoimmune diseases

Autoimmune diseases result from an aberrant immune response against the body’s own cells and tissues, impacting conditions like inflammatory bowel disease and rheumatoid arthritis. As soluble messengers, cytokines facilitate communication among immune cells, playing a key role in regulating the body’s response to pathogens.^368^ Although these drugs often result in adverse reactions, current treatments for autoimmune diseases typically involve drugs with anti-inflammatory and immunosuppressive properties. The development of drugs targeting cytokines or receptors, commonly known as “biologics”, represents a significant advance in treating autoimmune and inflammatory diseases. Nevertheless, while biologics have been therapeutically successful, they may not completely eliminate rheumatic pathology in all patients. Moreover, the efficacy of numerous such agents diminishes gradually owing to their immunogenicity.^369^ By modulating cell-to-cell signaling, it is feasible to suppress the hyperactivation of the immune system and diminish inflammatory reactions in autoimmune diseases. For example, introducing molecules like “pseudochain” proteins on the surface of T cells can disrupt signals between T cells and other cells, offering an effective approach to treating autoimmune diseases. Many cytokines hijack JAK and STATs for intracellular signaling in autoimmune and inflammatory diseases.^370^ Genetic mutations in JAK and STAT genes are linked to a range of immune deficiency syndromes and are connected to the development of autoimmune diseases. Given their roles in downstream signaling of cytokine receptors and growth factors, JAK/STAT pathways are considered as promising therapeutic targets for both cancer and autoimmune conditions.^371^ The efficacy of small-molecule JAK inhibitors in treating rheumatologic conditions illustrates the potential of targeting intracellular signaling pathways for autoimmune disease therapy.^372^

NK cells are lymphocytes of the innate immune system that make a rapid respond to diverse insults through cytokine secretion and cytolytic activity.^373–376^ They not only bring cell-mediated cytotoxicity to bear on tumor cells or infected cells but also regulate the effect of other immune cells via the secretion of cytokines and chemokines, thereby playing a regulatory role in the immune response.^377–379^. However, hyperactivation or malfunction of NK cells might be implicated in the etiology of specific diseases.^380,381^ In viral-induced models of autoimmune diabetes, NK cells are likely to contribute pathogenically in the later phases of autoimmunity.^382^ The presence of a large number of circulating NK cells may also be a nonspecific but significant characteristic of a predisposition to miscarriage.^380^ It is clear that NK cells possess dual roles, both protective and pathogenic, across various disease models, and occasionally even within the same disease. The cytokine environment and other stimuli acting upon various cell surface receptors in target organs, such as KIR, may trigger NK cell reactions distinctly and influence their contribution to various autoimmune disorders.^383^

EVs can directly interact with immune cells, activating or regulating their functions by carrying immune-activating molecules (IL-12/15/18).^384^ Some EVs can carry immune-suppressive molecules (TGF-β, PD-L1, etc.),^385^ exerting inhibitory effects on immune cells, helping tumor cells evade immune system surveillance. By delivering specific signaling molecules, EVs can promote the generation and function of regulatory T cells (Tregs), thereby playing a role in maintaining immune tolerance and preventing autoimmune responses. EVs can secrete soluble mediators, bind to receptors, and activate intracellular signaling pathways.^386^ while they can also act through direct membrane contact. Ultimately, this interaction may lead to the activation of membrane receptors on the target cells, thereby activating different signal transduction pathways.^387,388^ EVs express both self-antigens and peptide-MHC complexes. Therefore, EVs may represent a source of self-antigens and could potentially activate autoreactive T cells in the context of MHC.^389^ Exosomes are a subtype of EVs that are known to play a significant role in intercellular communication and antigen presentation. Exosomes secreted by antigen-presenting cells (APCs) theoretically possess the components necessary for antigen presentation and the activation of autoreactive T lymphocytes.^390^ Exosomes may act indirectly through interactions with APCs,^391–396^ especially for the initial activation of T cells.^396,397^ EVs can bind to APCs through adhesion molecules exposed on their surface along with self-antigen/MHC complexes, allowing the T-cell receptor to engage with the APC.^393^ The co-stimulatory molecules expressed by APCs provide the necessary second signal for the activation of T lymphocytes. This explains their involvement in the pathophysiology of autoimmune diseases through participation in phenomena such as inflammation and thrombus formation, vascular dysfunction, and the maintenance of autoimmune responses.^398^

### Respiratory diseases

Evidences suggest that multiple cell populations in the lungs work together to regulate the response of lung inflammation to direct and indirect stimuli.^399^ After infection or trauma, resident cells such as alveolar macrophages and alveolar epithelial cells secrete inflammatory cytokines into the alveoli, which induces a large number of inflammatory cells to migrate to the alveolar space. From these migrating inflammatory cells, inflammatory mediators are released and further lead to tissue damage and the development of acute lung injury and acute respiratory distress syndrome.^400^ The coordinated participation of neutrophils and macrophages in antimicrobial immunity serves as both inducers and effectors of adaptive immunity against extracellular and intracellular microbial pathogens. Neutrophils and macrophages play crucial roles in the innate immune response by phagocytosing pathogens and activating adaptive immune responses through antigen presentation and cytokine secretion. CCC between lung epithelial cells and alveolar macrophages plays an essential role in lung inflammation and injury.^401–404^ Polymorphonuclear neutrophil (PMN) accumulation and rapid infiltration in interstitial and alveolar spaces of the lungs is a hallmark of lung inflammation.^405^ Interactions of PMN with lung vascular endothelial cells contribute to the activation of specific endothelial cells responses involved in innate immunity.^406–408^

Asthma is the most common chronic respiratory disorder. CCC is highly involved in the pathogenesis of asthma in which epithelial-derived cytokines drive dendritic cell activation and phenotypic changes in the airways. These activated dendritic cells then migrate to secondary lymphoid tissues, where they present allergens to naïve T cells, triggering and perpetuating the allergic immune response in asthma.^409–411^ The above process is a typical inflammatory response in asthma-type 2 inflammation. During this process, interleukin (IL)-5 targeting eosinophils and IL-4 targeting lymphocytes are released.^412,413^ In asthma, mast cells and macrophages produce histamine, serotonin, and various inflammatory substances. These mediators promote smooth muscle contraction, increase mucus production, and enhance vascular permeability, ultimately leading to edema and contributing to the characteristic symptoms of asthma.^414^ Exosomes secreted from various cells, including respiratory epithelial cells, lymphocytes, mast cells, eosinophils, respiratory syncytial virus-infected cells, and lung epithelial cells, have the potential to contribute to or exacerbate asthma. These exosomes can carry bioactive molecules and signaling factors that may influence immune responses, inflammation, and airway remodeling in asthma pathogenesis.^415–426^

### Infectious diseases

Human immunodeficiency virus type 1 (HIV-1) causes a chronic infection leading to AIDS via infecting CD4 receptor-expressing (CD4^+^) immune cells. The HIV-1 envelope glycoprotein mediates two kinds of infection. One is called cell-free infection that infected host cells release virions to infect non-adjacent uninfected target cells. The other one is called cell–cell infection that infected host cells transmit HIV-1 to adjacent uninfected target cells via direct cell–cell connections called virological synapses.^427–433^ In addition to CD4^+^ immune cells, myeloid cells such as macrophages, dendritic cells, and osteoclasts are increasingly recognized as important target cells for HIV-1. These myeloid cells can play roles in various stages of the disease, including sexual transmission and early virus dissemination in both lymphoid and non-lymphoid tissues. They can serve as reservoirs for persistent viral infection, contributing to the establishment of a persistent viral library within the host. At least in vitro, these myeloid cells are rarely infected by cell-free infection. On the contrary, virus transmission through cell–cell infection may be the main mode of virus reproduction in vivo through the formation of tunneling nanotubes, homotypic or heterotypic cell–cell fusion and phagocytosis.^434–438^

The chronic infection of hepatitis B virus (HBV) impacts approximately 257 million individuals globally, leading to severe liver diseases, including cirrhosis and liver cancer. The interaction between healthy and HBV-infected cells, along with other cellular players such as innate and adaptive immune cells, is mediated through direct contacts and the exchange of diverse factors. This intercellular communication can be facilitated through various mechanisms, such as the release of metabolites, virions, protein complexes, and exosomes. These elements play crucial roles in modulating the immune response, viral replication, and the overall pathogenesis of HBV infection. Changes associated with HBV infection alter the CCC between hepatocytes and adaptive immune cells, significantly influencing the disease’s progression.^439–442^ Understanding these communication pathways is critical for unraveling the complex interplay between the virus and the host immune system, which can ultimately inform the development of effective therapeutic interventions against HBV infection.

The coronavirus disease 2019 (COVID-19) spread worldwide in a short period, resulting in numerous cases and associated deaths. A previous study revealed that monocytes in severe COVID-19 cases have the capacity to engage with CD8^+^ T cells, B cells and CD4^+^ T cells, chemokine receptors were also enriched in monocytes from severe patients.^443^ Chemokines interact with chemokine receptors to exert their biological effects, suggesting that these cytokines or their receptors could be potential treatments for severe COVID-19 patients and may become therapeutic targets for COVID-19 patients. Syncytia are large multi-nucleated cells produced by the fusion of two or more cells. Syncytial pneumocytes have been observed in patients who have succumbed to the COVID-19. The spike protein of SARS-CoV-2 interacts with the ACE2 receptor and is primed by the serine protease TMPRSS2 on neighboring cells, resulting in syncytia formation.^444^ Syncytia transmit viruses through CCC to immune cells and protecting the virus from neutralizing antibodies, thereby promoting infection. Rapid syncytial collapse trigger inflammatory immune responses which in turn leads to viral pathogenicity.^445–457^ CCC among myeloid, epithelial and T cells can drive tissue damage.^458^ Heterogeneous CCC patterns exist among moderate and severe patients across epithelial and immune cells in lung tissues.^459,460^ Exosomes participate in viral pathogenesis and spreading by CCC and induce cellular damage and multiple organ dysfunction such as inflammation, complement pathway, immune modulation, and coagulation in COVID-19. Moreover, the exosome-based vaccine that contains mRNA encoding the proteins of immunogenic COVID-19 has been developed. Furthermore, mesenchymal stem cell-derived exosomes and convalescent plasma-derived exosomes are new promising therapeutic strategies in severely affected COVID-19 patients.^461–467^

### Metabolic diseases

Metabolic dysfunction encompasses a spectrum of disease risk factors, encompassing hyperglycemia, dyslipidemia, hypertension, obesity, and insulin resistance. The pathogenesis of metabolic dysfunction is complex, involving a diverse range of cell types, tissues, organs, inflammatory signaling pathways, and humoral factors.^468^ EVs can carry substances of their parent cells (RNA, DNA, and lipids) and may provide the value of diagnosis and prognosis in metabolic dysfunction.^469–479^ They can mediate local communication between homologous cells within tissues (such as endothelial cells, hepatocytes, immune cells, or pancreatic cells) and traverse organ systems by entering the peripheral bloodstream.^480,481^ Therefore, EVs hold promise as biomarkers for predicting and diagnosing metabolic diseases.^475,477,482,483^ Specifically, platelet-derived EVs, marked by the presence of CD41, CD42b, and phosphatidylserine, have been implicated in various physiological and pathological processes, including exercise,^484,485^ acute injury,^486^ and diabetes.^487^

The highly complex pathogenesis of type 1 diabetes (T1D) is driven by several immune cells with effective response and regulatory characteristics, ultimately leading to the destruction of insulin-producing β cells. The interactions between immune cell groups and pancreatic islets are multifaceted. In both humans and mice, mutations in the FOXP3 gene, a key regulator of T regulatory (Treg) cell development, maintenance, and functionality, can precipitate severe autoimmunity, including T1D.^488,489^ An expanding corpus of research indicates that disruptions in Treg induction, stability, and functionality are central to the onset of islet autoimmunity and the clinical progression of T1D.^490–492^ Specifically, in the context of islet autoimmunity pathogenesis, the population of insulin-specific Tregs is markedly diminished, and both the induction and stability of Tregs are compromised in humans and mice.^493^ The pivotal function of Tregs in thwarting T1D aligns with the observed rapid disease advancement in NOD mice devoid of Tregs,^494^ and it has been found that Treg deficiency leads to heightened T-cell and NK cell infiltration in the pancreas.^495^ Studies have shown that Tregs from T1D patients exhibit dysfunctional IL-2 receptor signaling pathways.^496^ Administering a low dose of IL-2 for five consecutive days increased the number of pancreatic Tregs in NOD mice at the prediabetes stage by 1.5 times. This intervention prevent the progression of T1D in 60% of the treated animals and restored blood glucose levels to normalcy.^497^ Consequently, the targeted enhancement of Tregs through low-dose IL-2 administration emerges as a viable therapeutic approach.

Recent studies have investigated the role of EVs in regulating systemic metabolism, revealing that EVs originating from adipocytes serve as mediators linking obesity and insulin resistance in peripheral tissues (such as the liver).^498,499^ EVs facilitate communication between adipocytes and various cells within adipose tissue. To give an example, adipocyte-derived EVs can chemotactically attract monocytes, potentially leading to adipose tissue inflammation in obese insulin-resistant animals and humans.^500,501^ The formation of obesity and insulin resistance correlates with an increased infiltration of macrophages into adipose tissue.^502^ A vast body of literature describes the detrimental role of adipose tissue macrophages (ATMs) in regulating systemic metabolism through the overproduction of inflammatory cytokines that can block insulin signaling.^503^ Exosomes released by ATMs play a pivotal role in modulating the functionality of adipose tissue and insulin sensitivity.^504^ Patient plasma and urine enrich the sources of EVs, and studies suggest that EV miRNAs can serve as diagnostic tools for patients with metabolic.^472,477–479^ and cardiovascular diseases.^505,506^ The miRNA-155 is one of the miRNAs overexpressed in exosomes derived from obese ATMs. Studies indicate that miRNA-155 regulates adipose tissue homeostasis by directly inhibiting the adipogenic transcription factors peroxisome proliferator-activated receptor γ (PPARγ) and CCAAT/enhancer-binding protein β (CEBPβ).^507^ These findings shed light on the intricate signaling networks between adipocytes, stromal vascular cells, and distant organs in health and metabolic diseases.

### Developmental disorders

In developmental studies, different developmental time points or subpopulations can be chosen to study the dynamic interplay among diverse cellular varieties. For example, the receptor-ligand interactions analysis used to fine-map mouse hair follicle development indicated strong interactions between different subgroups at different developmental time points.^508^ The enriched receptor-ligand pairs of the same cell subgroups demonstrated strong autocrine signals, suggesting the involvement of robust intercellular communication in early hair follicle development.^508^ In addition, intercellular signaling across various cell types is essential for nervous system development, and ligand engagement plays a pivotal role in these developmental dynamics. Dysfunction of prefrontal cortex attributes to cognitive deficits and most neurodevelopmental disorders, so the intrinsic development-dependent signals that regulate neuron generation and circuit formation was unveiled, which gives a blueprint for comprehending the development of the human prefrontal cortex during the early and mid-gestational periods.^509^

A recent study revealed that five specific ligands (TGFβ2, NLGN1, TSLP, DKK1, and BMP4) have synergistic contributions on the progression of astrocytes in both human cerebral organoids and primary fetal tissues.^510^ Moreover, the synergistic impact of these ligands predominantly targets the mTORC1 signaling pathway, leading to the transcriptomics and morphological characteristics of astrocyte development. Furthermore, reciprocal signaling interactions between fetal germ cells (FGCs) and their gonadal niche cells were observed, showing that the cell proliferation of FGCs was promoted through BMP signaling pathway. Then, BMP signaling pathway regulates the Wnt signaling pathway by coordinating the chromatin accessibility of its ligand genes, providing a comprehensive roadmap for germ cell development in vivo physiologically.^511,512^ In addition, the Wnt/β-catenin pathway plays critical roles in embryonic development and adult tissue homeostasis.^513^ Furthermore, BMP signaling pathway plays a role in developmental stage dependence and cell-type specificity in male germ cells.^514^ On the other hand, analyses of PI3K signaling pathway unmasked the occurrence of specific CCCs during the kidney development of the human fetus, especially the interactions between reciprocal mesenchyme and epithelium cells, which may help the appropriate collecting duct epithelial cell morphogenesis.^515^

### Other applications

#### Biological transport system

The biological transport system plays a vital role in living organisms and enables the effective transmission and transportation of nutrients, gases, and information, thereby maintaining the normal operation of life. Molecular transportation in cells is mainly facilitated by protein channels and transporters. Regarding protein channels, their selectivity and effects on molecules can be further understood through the study of the structure and activity of proteins. As one kind of channel proteins that can transport glucose and other monosaccharides, *SLC2A4* has been found to be significantly downregulated in most cancers and its high mRNA expression is significantly related to improved prognoses in patients with breast cancer.^516^

For transporters, the main purpose of most research is to study their selectivity and activity. For instance, *Glut1*, the glucose transporter found in the caterpillar in Namibian Canyon, has been reported to increase due to gene expression or protein stabilization.^517,518^ Because they can maintain the energy needs of various biochemical procedures in tumor cells, it is important for us to understand how glucose intake of specific cell types affects the behavior of neighboring cells in the same microenvironment.^519^ In addition to intracellular transportation, extracellular transportation also plays a vital role. Extracellular transportation includes the exchange of substances between the matrix and extracellular fluids, the formation of extracellular matrices such as collagen fibers, and their role in tissue structure and cell life activities. The ECM is composed of a protein called collagen, which can support and protect cells, and promote interaction between cells.^520^ At the same time, the ECM can also regulate physiological processes such as signal transmission and the release of mediated factors in cellular activities.

Biological transport system technology leverages cell signaling molecules to regulate the transport and release drugs or other active substances. This approach can enhance the effectiveness and bioavailability of drugs, offering significant potential in disease treatment. For instance, cell signaling molecules can be harnessed to induce cancer cell death. In addition, ROS, produced during oxidative metabolism, serve as cell signaling molecules and are implicated in numerous human pathologies.^521^ They maintain biological system homeostasis through redox reactions and drive cellular regulatory pathways through subsequent signaling.^522^ But accumulation of excessive ROS promotes cell proliferation by triggering the pathological alteration of normal signaling processes, leading to the malignant transformation from normal cells. However, over-activated ROS levels could induce cell death by inflicting damage on cellular structures.^522^ Consequently, therapeutic approaches aimed at reducing excessive ROS to avert early tumorigenesis or enhancing ROS to selectively kill cancer cells show potential in cancer treatment.

#### Prediction of drug side effects

The study of CCC networks can assist in predicting the side effects of drugs. For instance, certain drugs work by activating or inhibiting specific signaling pathways, which may be involved in various physiological processes and result in adverse drug reactions. Various hormones, growth factors, and cytokines regulate cell proliferation and differentiation. These molecules engage with cellular receptors and interface with the cell nucleus through a series of intracellular signal transduction pathways. So when key components of these pathways undergo alterations due to oncogene mutation or over-expression, cancer cells emerge with disrupted cell signaling and uncontrolled cellular growth. These key components mutated in cancer cells present viable selective targets for innovative anticancer treatments, characterized by their tumor specificity and tolerable toxicity.^523^

## Experimental methods for studying CCC

### Visualizing CCC

#### Imaging methods

##### Electron microscopy

In the 1930s, the German scientist Ernst Ruska discovered that electrons could be focused under a magnetic field, leading to the invention of the world’s first transmission electron microscope (EM). Currently, scientists have invented cryo-electron microscopy (cryo-EM) on top of transmission EM, achieving “near-atomic resolution” of biological molecules, finally allowing humanity to glimpse how biological molecules execute their functions. The basic principle of cryo-EM technology involves placing a solution of biological macromolecules on an EM grid to form a very thin layer of water film, which is then rapidly frozen to liquid nitrogen temperature using cryo-techniques. The freezing speed is so fast that the water film does not form crystals but instead forms a vitreous ice layer. Biological macromolecules are immobilized within this thin layer of ice. Observing such frozen samples at low temperatures under a transmission electron microscope allows us to obtain the structure of biological macromolecules.

The nicotinic acetylcholine receptor (nAChR) is a transmembrane protein that facilitates swift cellular communication under the influence of acetylcholine, an endogenous neurotransmitter. As a typical transmembrane macromolecule, it has extensive interactions with the surrounding lipid microenvironment. Recent cryo-EM studies have unveiled the presence of phospholipid and cholesterol sites within the lipid-exposed regions of neuronal and electric organ nAChRs.^524^ These findings are consistent with previous spectroscopy and affinity labeling studies, which suggested that lipid molecules closely interact with the transmembrane segments of the receptor. For example, electron spin resonance (ESR) studies provided preliminary evidence of motion-restricted lipids in contact with nAChRs in native torpedo membranes.^525–527^ In subsequent ESR experiments using recombinant nAChRs, direct contact between the receptor and adjacent or boundary lipids was confirmed.^528^ The emerging data offers structural evidence supporting the proposed “lipid sensor” function of the outer loop of the M4 transmembrane domain and its regulatory impact on nAChR functionality.

For another example, the transient receptor potential vanilloid 1 (TRPV1) channel is a multimodal receptor that can respond to various stimuli, such as heat, capsaicin, and protons, making it a crucial pain sensor and an effective target for anesthetic drugs.^529,530^ With the employment of cryo-EM, the channel structure of the membrane protein TRPV1 was resolved at near-atomic resolution, along with its structure in complex with capsaicin.^531^ As an extracellular chemical signal, capsaicin binds to the membrane protein TRPV1 located at the nerve endings on the tongue, opening a channel on the membrane protein that allows ions to flow from outside the cell membrane to the inside. This ion movement, albeit minor, generates a current that is ultimately transmitted to our brain through nerve fibers, enabling us to experience the sensation of spiciness.

CCC plays a pivotal role in the structuring and functionality of cellular networks and multicellular systems.^532^ This intricate network is coordinated by signals emanating from the microenvironment, such as paracrine or autocrine actions of soluble factors or stimuli mediated by substrates from the ECM.^533,534^ In addition, direct CCC happens through structures such as gap junctions and tunneling nanotubes (TNTs),^535–537^ which are thin bridges formed by the cytoskeletal actin filaments, capable of transferring cytoplasm and organelles between connected cells.^538^ Scanning electron microscopy (SEM) is a crucial tool for directly observing cell-to-cell TNTs.^539,540^ To date, a significant number of TNTs have been observed in single cells or three-dimensional tumor cell aggregates.^537,541,542^ Mesenchymal stem cells have been extensively studied due to their accessibility, multipotency, and potential for anti-inflammatory and pro-angiogenic effects. SEM was first employed to investigate a large population of MSC’s spheroids and revealed the presence of TNTs within homotypic three-dimensional clusters formed by human MSCs. These observations were facilitated through direct visualization using SEM and laser scanning confocal microscopy.^543^

##### Optical microscopy

EM imaging requires fixed cells and special treatments such as dehydration and embedding, making it unsuitable for live-cell imaging, and whether the images obtained can truly reflect the structural information in living cells is also uncertain. Optical microscopes, along with various fluorescence microscopy imaging techniques developed later in combination with fluorescent labeling, are one of the indispensable means for studying life sciences and biomedical issues at the cellular level. In wide-field epifluorescence microscopy, spatial resolution is easily distorted due to defocus blur, especially when fluorescent molecules are distributed in three dimensions and form densely packed structures, as is typical with biological samples. Confocal microscopy uses a pinhole to completely eliminate out-of-focus blur, achieving optical sectioning.^544^ Two-photon microscopy utilizes the two-photon absorption process to excite fluorescent molecules, where fluorescence occurs only at the focal point of the objective, thus providing an optical sectioning effect.^545^ However, the spatial resolution of such microscopy techniques is still limited by diffraction. Therefore, advancements in the life sciences urgently call for innovations that can unveil nanoscale molecular dynamics and structural intricacies within living cells, surpassing the diffraction limit to enhance the resolution of optical microscopes.

As the first far-field microscopy imaging technique to break the optical diffraction limit, stimulated emission depletion (STED) microscopy achieves a three-dimensional resolution of 30–50 nm through the use of nonlinear effects.^546–548^ Due to its high temporal resolution and three-dimensional tomographic capability, it represents an important direction in the development of optical super-resolution techniques. Pellett et al. first achieved live-cell dual-color STED imaging and used improved SNAPf and CLIPf labeling techniques to label EGF and EGFR for observing their interactions.^549^ Furthermore, the use of STED-FCS (fluorescence correlation spectroscopy) combined techniques to study the interactions between membrane proteins or lipid molecules has also become a hot topic in modern biological research.^550,551^

Optical activation techniques, including stochastic optical reconstruction microscopy (STORM), have significantly improved the spatial and temporal resolution available for examining the physical interactions between cells.^24^ STORM selectively activates multiple photo-switchable fluorescent groups to determine the lateral position of each fluorescent source, enabling the reconstruction of individual images with nanoscale resolution.^552^ The three-dimensional extension of STORM, known as 3D-STORM, integrates enhanced axial resolution, offering a valuable tool for probing the organization of proteins at the cell–cell interface within dense tissues or environments characterized by numerous uniform cell interactions.^553^ In brain tissue, this technique facilitates the detailed observation of the organization of scaffolding proteins and neurotransmitter receptors within synapses.^554^

STED and STORM imaging techniques achieve high resolution; however, a drawback is the requirement for intense excitation light for illumination. In addition, the fluorescent groups in the specimen are quickly bleached, and the generated free radicals have the potential to cause damage to the specimen. Therefore, these imaging modes are more suitable for fixed specimens rather than for observing and studying live biological samples. Consequently, another mode of achieving super-resolution imaging through altering illumination has emerged.

Structured illumination microscopy (SIM) applies patterned illumination fields instead of conventional wide-field illumination, improving the spatial resolution of optical microscopy and providing benefits for observing live cells.^42^ In optical microscopy, the objective lens has a limited ability to collect high-frequency information from the sample, resulting in the loss of such details during imaging. SIM technology addresses this limitation by using Moiré fringes to transfer these high-frequency details, which exceed the lens’s collection capacity, to the low-frequency range. This enables the microscope to capture information that was previously unattainable. By applying specific image algorithms to process this combined low-frequency and high-frequency data, SIM produces super-resolution images that are approximately twice as detailed as those obtained through traditional optical microscopy.^42,43^ Because of its quick imaging speed, minimal phototoxicity, and broad dye compatibility, SIM is highly appropriate for prolonged monitoring of dynamic events in living cells.

#### Fluorescence resonance energy transfer

Fluorescence resonance energy transfer (FRET) is the mechanism used to detect interactions between two biomolecules, allowing for the inference of their spatial proximity. This process involves the transfer of energy from an excited donor fluorophore (D) to a compatible acceptor (A) protein or fluorophore through a non-radiative means. The donor absorbs energy at shorter wavelengths, while the acceptor absorbs energy at longer wavelengths.^555,556^ This process only occurs when the two molecules are in very close proximity, a distance that is associated with the formation of complexes and conformational changes involving most biomolecules or their constituent domains.^557^ When the distance is less than 1 nm, the donor and acceptor collide, and when the distance is greater than 10 nm, the donor emits photons. Therefore, FRET only occurs in the near field, within a range of 1–10 nm.^558,559^ When two closely positioned molecules are fluorescent substances, the observable effects of FRET will manifest in the spectral properties of these fluorescent dyes, including alterations in fluorescence intensity, fluorescence lifetime, quantum efficiency, and anisotropy.^560,561^

FRET is exceptionally well-suited for measuring a wide range of dynamic molecular events, including the conformational alterations of macromolecules, both cis and trans binding and/or assembly of macromolecules, as well as the modulation of physiological events across both in vitro and in vivo settings.^556^ Traditional optical microscopes are constrained by lateral diffraction to a spatial resolution of ~250 nm, a scale that exceeds the average size of protein molecules by several orders of magnitude within a range of a few nanometers.^562^ This makes it difficult to predict whether two molecules are interacting in an image obtained by traditional microscopy. In contrast, utilizing FRET increases the accuracy of molecular colocalization within the diffraction limit. In principle, any instrument capable of recording fluorescence emission can be used to measure FRET, given the presence of appropriate fluorophores along with corresponding filters and detectors. Therefore, early FRET experiments were primarily conducted using fluorescence spectroscopy,^563–565^ which gradually evolved to flow cytometry^564,566,567^ and various microscopes,^564,568–571^ and later developed into laser scanning cytometry.^572–574^ FRET allows researchers to directly observe interactions between specific proteins within living cells, which is crucial for understanding intracellular signaling networks. FRET is used to study single-molecule interactions,^575^ within living cells,^576^ and even across entire tissues.^577^

FRET probes have been developed to probe various processes in cellular signal transduction.^578,579^ These powerful approaches allow for in vivo imaging across systems ranging from Caenorhabditis elegans to transgenic mouse models expressing FRET probes.^580–582^ Imaging methods for measuring FRET encompass epifluorescence and confocal microscopy on ex vivo tissues, skin samples, or isolated vessels and/or tissue specimens, extending to multiphoton imaging within intact tissues.^581–584^ In addition, lifetime FRET measurements were achieved based on multiphoton imaging and fluorescence through a cranial window in mouse models of the nervous system.^585^ The potential of utilizing FRET-based probes in combination with in vitro cell cultures, ex vivo tissue preparations, and in vivo model systems for investigating cellular signaling systems is highly compelling.

#### Cell surface detection methods

In supported planar lipid bilayers (SLBs), fluorescently labeled proteins are incorporated into the lipid bilayer to facilitate imaging of protein movement and organization, thereby enabling tracking throughout the entire CCC process.^586^ SLBs have evolved into a platform for studying molecular patterns.^587–589^ Using SLB techniques, it is possible to measure the two-dimensional affinity and kinetic rates of contact areas, thereby providing a quantitative basis for understanding the interactions within contact zones.^590^ Besides, GRASP is a protein complementation strategy that fuses two nonfluorescent fragments of GFP to interacting partners on opposing cells to detect CCCs. When cells are in close contact with each other, the split protein fragments associate and reassemble into GFP.^45^ GRASP has been applied to study both pre- and postsynaptic interactions, enabling the analysis of connectivity and the distribution of inhibitory and excitatory synapses in mouse hippocampal neurons.^591^ This approach has been expanded to include other split fluorescent protein fragments, such as YFP (yellow) and CFP (cyan), allowing for simultaneous imaging of multiple synaptic interaction factors.^46^

Another reported strategy for monitoring interacting cellular partners involves a chemo-genetic system that utilizes fluorogen-activating protein (FAP) in combination with a dye activated by proximal anchoring (DAPA), which is composed of malachite green and chloroalkane.^592^ FAPs serve as a fusion protein tool that acts as a fluorescent marker by binding to nonfluorescent dyes known as fluorogens.^593^ Malachite green is displayed on cells expressing HaloTag through attachment with chloroalkane, and when it comes into contact with adjacent cells expressing FAP, the contact between cells is reported through enhanced fluorescence. When fluorogens bind to FAP, this fluorescent readout can target different subcellular locations.^594^ and be expressed in various model species.^595–598^ Furthermore, enzyme-based amplification methods have also contributed to enhancing the visualization of CCCs.^599,600^

### Chemically tagging CCCs

#### Contact-dependent tagging

Contact-dependent labeling techniques necessitate the physical interaction between an enzyme presented on the surface of one cell and a receptor substrate on a neighboring cell to facilitate cell-to-cell proximity labeling.^24^ The labeling immune partnerships by sortagging intercellular contacts (LIPSTIC) utilizes a modified sortase enzyme derived from Staphylococcus aureus (SrtA) that can be fused to a cell surface ligand. This enzyme transfers a biotinylated substrate to a pentaglycine receptor peptide present on a matching receptor of adjacent cells, enabling the identification of receptor-ligand interactions within living animal cells. LIPSTIC has successfully facilitated the direct biotinylation of various ligand–receptor pairs (LRPs) by exploiting physical interactions.^601^ So LIPSTIC allows for the direct measurement of dynamic CCCs both in vitro and in vivo. Through the application of LIPSTIC, it has been shown that the interactions between dendritic cells and CD4^+^ T cells during T-cell priming in vivo through two distinct phases: an initial, cognate stage marked by CD40–CD40L interactions unique to T cells and antigen-presenting dendritic cells, and a subsequent, non-cognate stage where these interactions no longer require prior activation of the T-cell receptor.^601^

Another technique called enzyme-mediated cellular proximity labeling (EXCELL), which is based on a similar sortase enzyme mechanism, utilizes an enhanced form of the Staphylococcus aureus transpeptidase sortase A enzyme (mgSrtA).^602^ This variant has the ability to covalently tag a range of cell surface proteins that contain a single glycine residue at their terminus. This technique enables high-resolution imaging of CCCs, allowing for in-depth examination of the molecular composition and structure at the contact sites. It also minimizes perturbation to the natural state of cells, ensuring that the observed interactions closely resemble physiological conditions. By avoiding the need for pre-installation of oligoglycine, EXCELL holds the potential to detect novel cell interactions.^602^ In particular, it has been used to monitor CCCs in living mice since the small pentapeptide “LPETG” can be easily conjugated with other molecules.^601^ In short, EXCELL could become a powerful tool for detecting and discovering CCCs in more complex in vivo environments.

The necessity of genetically incorporating labeling enzymes might pose a significant obstacle to the widespread application of these methodologies in the comprehensive study of CCCs. Interaction-dependent fucosylation (FucoID) circumvents this challenge by autonomously anchoring the labeling enzyme Helicobacter pylori α1,3-fucosyltransferase onto the cell surface.^603^ The efficacy of immunotherapies aimed at bolstering endogenous T-cell immunity hinges upon the T cells’ capacity to identify tumor-specific antigens (TSAs).^604^ In the quest to expedite advancements in cancer immunotherapy, the development of a computation-free methodology that enables the swift identification of TSA-reactive T cells, and is straightforward to implement, is highly desirable. FucoID emerges as a pivotal innovation, capable of identifying endogenous tumor antigen-specific T cells through interaction-dependent fucosylation without prior knowledge of TSA identity. Employing this approach facilitates the isolation of TSA-reactive CD4^+^, CD8^+^ T cells, and TSA-suppressive CD4^+^ T cells within tumors.^603^ This technique exhibits wide-ranging utility across multiple mouse tumor models characterized by observable T-cell infiltration, underscoring its significant testing prospects in clinical scenarios.

#### Contact-independent tagging

Non-contact techniques, in contrast to contact-dependent labeling methods, generate highly reactive labels capable of diffusing beyond the catalyst’s immediate vicinity. Proximity labeling provides a method to capture the immediate biochemical environment of proteins in situ, thereby preserving key spatial and temporal contexts.^605^ When integrated with mass spectrometry (MS)-based proteomics, these approaches enable the elucidation of the proteomic landscape of spatially restricted cell–cell interfaces. This integration provides critical insights into the manner in which the structural organization of proteins affects the functional consequences of CCCs.^24^

APEX has been used to capture the entire organellar proteome with high temporal resolution and has become an important tool for proximity labeling. In the presence of H_2_O_2_, APEX converts biotin-phenol (BP) labels into short-lived (t_1/2_ ≈ 100 μs) reactive phenoxy radicals, marking neighboring proteins on tyrosine and other electron-rich amino acid side chains in the mitochondrial matrix.^606^ For example, combining proximity labeling with quantitative proteomics can capture the location and timing of GPCR function in living cells.^606^

The well-known proximity labeling method BioID utilizes a BirA ligase mutant (BirA*) to biotinylate proximal proteins.^607^ In the presence of ATP, BirA* catalyzes the conversion of biotin into active biotin-AMP, which then reacts with nearby nucleophilic lysine side chains. The BioID method is primarily used to identify intracellular binding partners,^605^ including the cytoplasmic region of cadherins.^608–610^ and other adhesion proteins.^611^ BioID fused to the extracellular domain of N-cadherin (Ncad) has also identified proteins secreted by rat neurons.^612^ By combining proximity labeling with single-molecule binding analysis, previously undisclosed direct connections have been unveiled between the extracellular domains of numerous transmembrane proteins and E-cadherin (an essential cell–cell adhesion protein).^613^ As the labeling efficiency with BirA* was found to be slow (taking 18–24 h), a more rapid system called TurboID was created, capable of completing labeling in a mere 10 min.^614^ The TurboID technique has been employed to identify proteins at epithelial cell junctions by fusing the enzyme with the extracellular domain of E-cadherin.^613^

### Mechanical force analysis

Although the shape of organisms is encoded in their genomes, the information coded by DNA is not enough to rule the ultimate architecture of tissues and organs, nor can the cell expression profiles tell us how complex functions are achieved. The developmental trajectories culminating in the definitive morphology of vertebrates involve continuous feedback between dynamic mechanical forces along with cell growth and movement. Mechanical forces are ruled by cells and integrated into tissues through mechanotransduction processes that affect cell shape, proliferation, migration, and programmed cell death, collectively sculpting the final form of organism.^615^ The core of these processes is primarily the myosin motors, and the quasi-stable state of cell tension is maintained by these myosin contraction force mechanosensors, allowing cells to define the shape and tension of organs.^615^ The initial discovery that cancer cells can grow in soft agar in an anchorage-independent manner,^616,617^ while most noncancer cells cannot, sparked interest in the role of mechanotransduction at the cellular level. Red blood cells exposed to anionic and cationic drugs undergo different changes in intracellular and extracellular surface membrane tension, resulting in modifications to cell morphology.^618^ This observation implies the presence of a cellular mechanism capable of detecting changes in membrane tension, which was subsequently demonstrated to be crucial for cell spreading and migration.^619^

Cell migration plays a crucial role in many physiological and pathological processes such as morphogenesis,^620^ wound healing,^621^ and tumor metastasis.^622^ In turn, migration involves a coordinated series of events, including the protrusion of pseudopodia, formation of new adhesions, development of traction forces, and release of old adhesions.^623^ To achieve appropriate physiological outcomes, cell movement must maintain a certain direction and speed in response to environmental stimuli. Traction force microscopy (TFM) is a technique used to measure the forces exerted by cells on their substrate.^624–626^ It is based on the principle that cells generate traction forces on their attached substrate during migration, extension, or contraction. One can infer the magnitude and direction of the forces exerted by the cells by observing the minute deformations on the cell-attached substrate. However, TFM is limited by poor resolution, typically confined to detecting forces on a micrometer scale.^627,628^ Therefore, a strategy has been proposed to enhance the output of TFM by increasing the achievable head density and the accuracy of head tracking.^629–631^ This involves combining an algorithm for fluctuation-based super-resolution (FBSR) imaging with software-enabled super-resolution microscopy. Through the analysis of fluorescence group intensity fluctuations, this approach allows for the resolution of densely packed beads and significantly improves the traction force output.^632^

The arrangement of intermolecular forces in space dictates the interplay among macromolecules, with both long-range and short-range interactions playing pivotal roles in the dynamic behavior of biological systems and their assemblies.^633^ Atomic force microscopy (AFM) is a high-resolution scanning probe microscope used to study the interaction forces between objects at the nanoscale.^625,626,634,635^ AFM enables a direct measurement of intercellular interactions by delicately contacting the probe with the cell surface and subsequently capturing the force-displacement curve as the probe interacts with the cell surface. The method for examining the spatial distribution of forces within a volume using AFM involves collecting a series of force curves on the surface and assembling them into a “force volume” (FV).^633^ Radmacher et al. used the Hertz model to analyze the FV images of platelets and constructed the first cell mechanical properties map based on AFM.^636^ A similar methodology was employed to investigate the contribution of the actin cytoskeleton to the local mechanical characteristics of cardiomyocytes.^637^ and macrophages.^638^ However, achieving atomic resolution imaging has long been challenging when nanoscale manipulation under different environments becomes routine. The initial deficiency was due to the contact with the sample dulling the atomic tip, which is essential for successful atomic resolution imaging.^639^

With the introduction of non-contact atomic force microscopy (NC-AFM), this issue was finally overcome. In NC-AFM, the cantilever oscillates near the surface of the sample without actually “touching” it, allowing the preservation of the tip’s atomic sharpness while quantifying the tip-sample distance using the changes in the cantilever’s resonance frequency caused by interactions.^639^ The latest progress in high-speed atomic force microscopy (HS-AFM) has enabled the examination of conformational dynamics in individual unlabeled transmembrane channels and transporters. The progress in HS-AFM now allows not only for the detection of faster dynamics but also provides sub-molecular structural information in real space,^640–643^ significantly improving temporal resolution.^644^ The emergence of HS-AFM, characterized by unprecedented scanning rates, results from a blend of diverse technological advancements. These include enhancements in cantilever beams, sample stage scanners, cantilever beam deflection detection, and feedback systems.^645^ HS-AFM imaging has succeeded in applying to various biological systems, such as molecular motors,^641^ membrane-associated proteins,^646^ macromolecular systems,^647^ and protein–DNA complexes.^648^

## Downstream analysis and experimental validation

Research on molecules in signaling pathways that play a key role in regulating growth and development has revealed that the response process is not the result of a single pathway’s action but rather the result of crosstalk between different pathways.^649^ Accurate signal transduction requires crosstalk between various pathways, further forming complex intracellular signaling networks. Cellular signal transduction begins at the cell membrane, propagates through the cytoplasm, and ultimately regulates gene expression patterns deep within the nucleus. This process is mediated by a series of typically weak and transient protein–protein interactions, enabling cells to rapidly adapt to changing environmental conditions.^650^ To fulfill their critical roles in cellular processes, these proteins interact with each other stably or transiently, forming a vast network.^651,652^ The application of proteomics and cell manipulation techniques in cell signal transduction research provides important means for revealing complex intracellular signaling networks and identifying signal molecule complexes. They also enable exploration of the molecular basis of protein–protein interactions, discovery of new partner molecules, and study of the crosstalk between known pathways and the dynamic changes in cell signal transduction.

### Co-immunoprecipitation

Co-immunoprecipitation (Co-IP) is one of the strongest methods for identifying physical interactions between two or more proteins in vivo.^653–655^ It is a technique where antibodies are used to precipitate a specific molecule, and other molecules that specifically bind to that molecule are co-precipitated along with it. This technique is commonly used to verify the specific binding between proteins.^656^ Co-IP is able to identify protein interactions involved in the cell communication process, including interactions between receptors and ligands, signal transduction molecules, and the activation of effector proteins. These interactions form the basis of how cells respond to external signals and trigger internal responses. Protein interactions, forming complexes of varying sizes, exhibit spatiotemporal dependency.^657^ The execution of specific protein functions strongly relies on contact with the surfaces of neighboring proteins. Most processes demand direct contact between proteins, either in binary form or as part of large complexes involving multiple proteins.^658^ Co-IP can identify protein complexes formed under specific cellular states or conditions, thereby revealing key participants in the cell communication process. In most cases, these in vitro binding assays are combined with MS. A previous study utilized the Co-IP/MS method to identify BMPR-1B protein–protein interactions (PPIs). In addition, the signal pathway of the target protein was analyzed, and bioinformatics prediction indicated that BMPR-1B interacts with ovulation-promoting proteins in ewe ovaries.^659^ As a transmembrane protein, BMPR-1B mediates signal transduction between the intracellular and extracellular compartments by participating in vital activities and substance exchange.^660^

In addition, Co-IP technology can be used to explore unknown protein interactions, thereby discovering new signaling molecules and pathways involved in cell communication. Through Co-IP and GST pull-down assays, Angiogenin (ANG) was reported for the first time to interact with ribonuclease inhibitor (RI) both endogenously and exogenously.^661^ Upregulating ANG, including the ANG His37Ala mutant, significantly decreased RI expression and activated phosphorylation of key downstream target molecules of the PI3K/AKT/mTOR signaling pathway.^661–663^ This discovery led to the promotion of tumor angiogenesis, tumorigenesis, and metastasis in vivo, highlighting a novel mechanism of ANG in regulating the PI3K/AKT/mTOR signaling pathway via RI. Therefore, PPIs play a crucial role in almost every cellular process since they dictate the specificity of signal transduction, control the strength and duration of signals, and integrate various signaling pathways to orchestrate intricate cellular responses.^664^. In turn, understanding PPIs will help elucidate the pathophysiology and progression of many diseases.^665^

### Functional exploitation

Although the previously mentioned methods are adept at identifying proteins in close proximity and potential ligand–receptor pairs, further approaches are necessary to explore and interfere protein functions at the cell–cell interface. Cell manipulation techniques that induce the loss, obtainment, or modification of protein functionalities offer a direct avenue for investigating ligand–receptor interactions or the ensuing signaling pathways. This approach helps in gaining insights into the types of cellular interactions that take place, the consequences of these interactions, and how to leverage them by manipulating transcriptional programs.^24^ These methods, by altering cell behavior, communication modes, or environmental response capabilities, play a crucial role in both basic research and clinical applications.

CRISPR-Cas9 screening has been employed to comprehend the functional roles of proteins implicated in the evasion, recognition, and clearance of cancer cells during the adaptive immune system.^24^ Alterations in somatic genes can modify the susceptibility of cancer cells to T-cell-based immunotherapy. To identify proteins in tumor cells that regulate and/or are sensitive to T-cell effector functions, a dual-cell-type CRISPR (2CT-CRISPR) screening method was devised for conducting loss-of-function analyses.^666^ Interferon-gamma (IFN-γ) driven phosphorylation of JAK1 stimulates the JAK-STAT signaling cascade to enhance antigen processing and presentation in tumors, thereby enhancing T-cell recognition and cytolysis.^667^ Utilizing a co-culture system of IFN-γ signaling-deficient tumor cells and T cells, CRISPR-Cas9 screening identified several genes within the TNF signaling pathway as critical to rendering tumor cells vulnerable to T-cell-mediated eradication, thereby unveiling potential targets for alternative immune therapeutic pathways.^668^

**Fig. 4:**
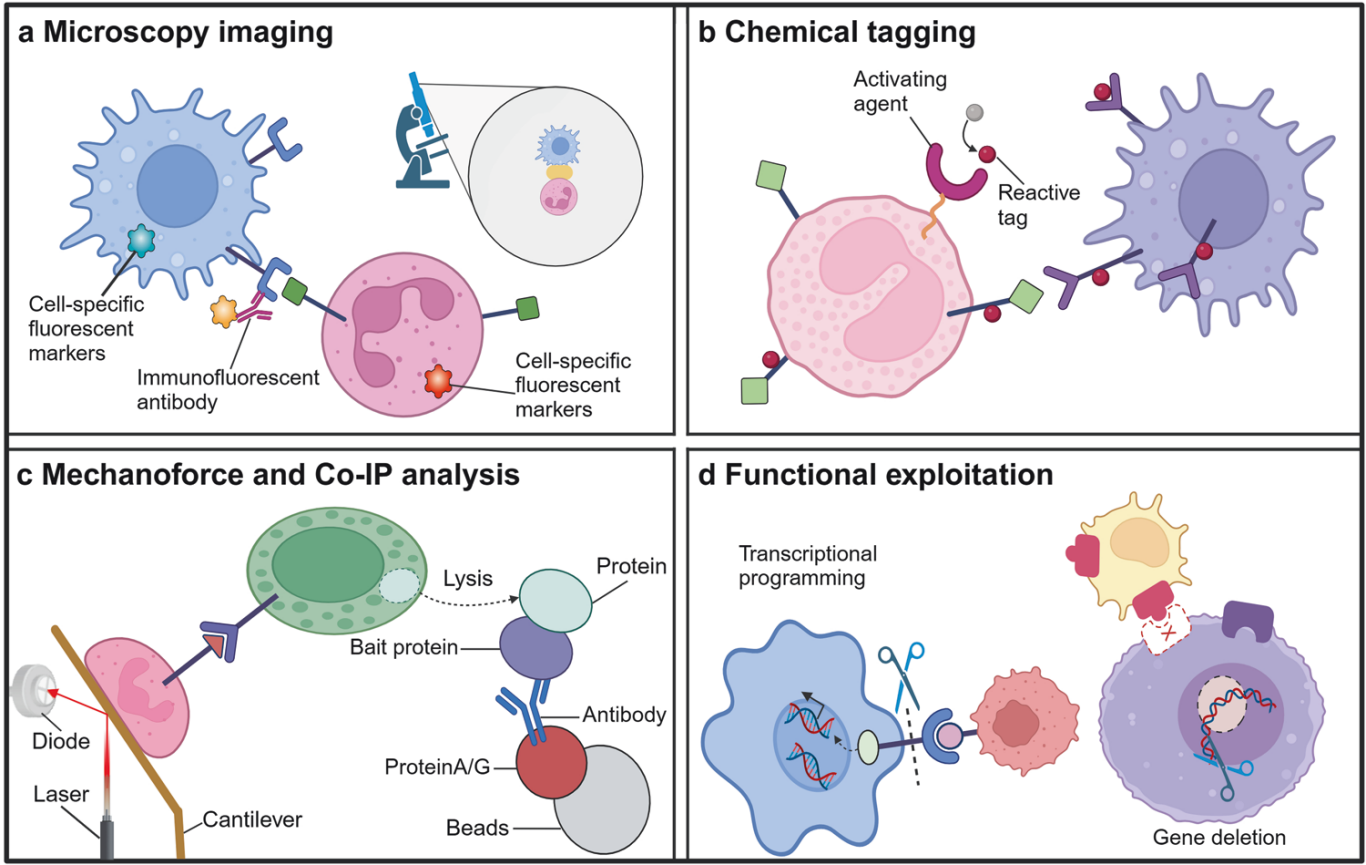
Representative experimental methods for studying CCC. Technologies to expand the molecular-level understanding of cell–cell interaction biology include **a** microscopy imaging, **b** chemical tagging, **c** mechanoforce, and Co-IP analysis, and **d** functional exploitation

Activation of downstream biological processes mediated by the cell surface can also be achieved through the engineering expression of receptors and/or ligands on the cell surface. Synthetic Notch (synNotch) receptors provide extraordinary flexibility in engineered cells, allowing for the customization of sensing/response behaviors based on user-specified extracellular signals.^669–671^ SynNotch receptors are engineered to incorporate the core regulatory domains of the Notch cell–cell signaling receptor, but with synthetic extracellular recognition domains (such as single-chain antibodies) and synthetic intracellular transcriptional domains.^669,672^ This Notch intracellular domain acts as a transcriptional regulator, operational only after its release from the membrane and enable it to enter the cell nucleus to activate genes pivotal for cell–cell signaling during developmental processes.^673^ These synthetic Notch receptors are versatile, functioning across various cell types, including immune cells and neurons. The deployment of multiple synthetic Notch pathways is allowed within the same cell and used to design complex combinatorial sensing circuits. The flexibility of synthetic Notch receptors in engineering new cell behaviors makes them a powerful tool for constructing therapeutic cells, driving the formation of complex multicellular patterns, or regulating or reporting cell behavior in complex in vivo environments.^669^ Natural T-cell response programs lack certain desirable characteristics.^674^ To give an example, even when redirected to identify tumors, T cells have limited ability to overcome the immunosuppressive microenvironment of tumors.^675^ But T cells engineered with synthetic Notch receptors exhibit robust and finely tunable customized functionalities. In addition, T cells equipped with synthetic Notch circuits can precisely home in on solid tumors, enabling the localized delivery of their tailored potent payloads within the body.^670^ (Fig. 4).

## Computational methods for inferring CCC

**Fig. 5:**
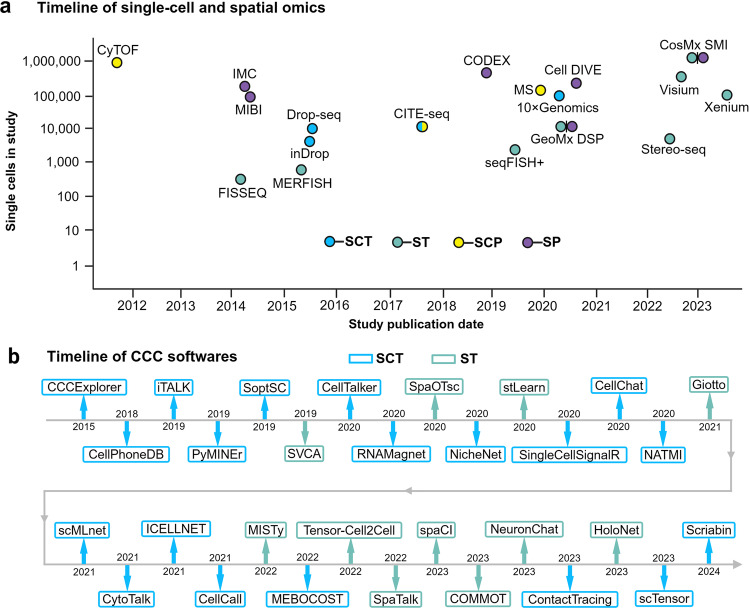
The timeline of single-cell and spatial omics and related CCC softwares. **a** Timeline of the key technologies for single-cell and spatial omics were retrospectively summarized from 2011 to the present day. Cell numbers reported in representative publications by publication date. A full table with corresponding cell numbers is available as Supplementary Table 1. SCT single-cell transcriptome, ST spatial transcriptome, SCP single-cell proteomics, SP spatial proteomics. **b** The history of various bioinformatics and computational methods developed to infer biological cell–cell communications based on single-cell omics data. SCT single-cell transcriptome, ST spatial transcriptome

**Fig. 6:**
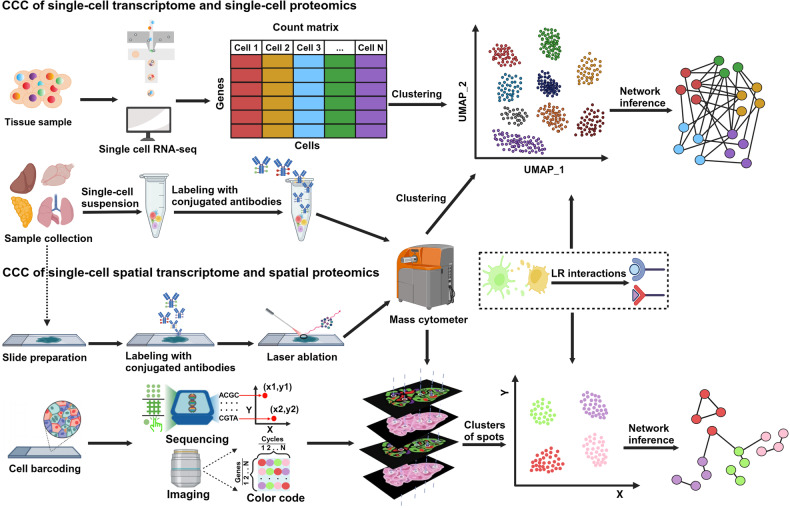
CCC networks inferred from single-cell omics. Intercellular communication networks can be inferred through various single-cell multi-omics techniques and methods. (**1**) CCC of single-cell transcriptome: gene expression matrices of different cell types are obtained by performing single-cell RNA-seq, and then clustering analysis is carried out to infer communication networks of various cell types. CCC of single-cell proteomics: a single-cell suspension is made after collecting samples such as liver, pancreas, lung and mouse brain which labeling with conjugated antibodies tagged with metal isotopes. Then cell–cell communication of different cell types is inferred through mass spectrometry flow cytometry and clustering analysis. (**2**) CCC of spatial proteomics: tissues are prepared on slides followed by labeling of conjugated antibodies tagged with metal isotopes and laser ablation, then protein expression map and CCC network is obtained by analysis of ion mass spectrometry. CCC of single-cell spatial transcriptome: by combining scRNA-seq with spatial localization, gene expression map of various cell types is obtained to infer CCCs in different spatial locations

Using single-cell omics data, various bioinformatics and computational methods have been developed to decipher biological CCCs.^676^ (Table 2). Scientific research commonly adopts two principal approaches: ligand–receptor (LR) signal-based algorithm and physical location-oriented strategy.^7^ The availability of single-cell data, particularly transcriptome data, has led to the development of plenty of computational tools for deciphering CCC (Fig. 5). These tools leverage diverse methods for predicting potential intercellular communication events (CEs) based on prior knowledge of CCCs.^4^ Various mediators facilitate the development of tools for CCC analysis, including Ca^2+^,^677^ lipids,^678^ peptides,^679^ proteins,^680^ EVs,^681^ and electrical signals. These developed tools for CCC analysis utilize different media, algorithms, and data types to infer CCC, leading to the discovery of different types of CCCs based on different principles and resulting in various visualizations (Fig. 6).

**Table 2:** Existing bioinformatic tools for inferring CCC

Existing bioinformatic tools for inferring CCC										
ID	Tool	Feature	Algorithm	Link	Input	Output	Visualization	Available in	URL	Refs
1	CellPhoneDB	A database of ligands, receptors, and their interactions; The subunit architecture of ligands and receptors	Expression permutation	L-R	scRNA-seq	Upregulated and downregulated interactions; List of most statistically significant L-Rr interactions	Heatmap; Dot plots; Cluster combinations	Python and Web interface	https://github.com/Teichlab/cellphonedb	^688^
2	CellChat	Intercellular interactions ; Communication networks; Cell interaction network diagrams and communication pathway	Expression permutation	L-R	scRNA-seq	Likelihood of CCC between all clusters for all interactions	Alluvial and Circos plots; Dot plots	R and Web interface	https://github.com/sqjin/CellChat	^686^
3	ICELLNET	Summing the product of all LRI scores between two clusters to compute an overall CCI score	Expression permutation	L-R	scRNA-seq	Intergroup communication scores; matrix of CCC probabilities	Bar plots; Network visualization	R	https://github.com/soumelislab/ICELLNET	^689^
4	SingleCellSignalR	The ligand–receptor interactions that underlie cellular networks; A new curated LR database and a novel regularized score to perform inferences	Expression permutation; A regularized score to assess the confidence in predicted ligand–receptor interactions	L-R	scRNA-seq	Interaction scores for each LRI between all clusters in the dataset	Circos plots, tables and graph visualizations of interactions between clusters	R	https://github.com/SCA-IRCM	^690^
5	CellCall	Identifing the significantly activated pathways involved in intercellular crosstalk between certain cell types	Expression of ligands/receptors and downstream TF activities	L-R-TF	scRNA-seq	Intracellular signaling and a threshold for intercellular communication scores	Circos plots; Sankey plots; Bubble plots; Ridge plots, etc.	R	https://github.com/ShellyCoder/cellcall	^691^
6	NATMI	Interactions between clusters are modeled, calculated by the product of normalized ligand and receptor expressions of the two clusters	Mean-expression weight; Specificity weight; Cell-connectivity-summary-network edge weights	L-R	scRNA-seq	Summarizing how strongly (or specifically) each cell type is communicating to another cell type	Heatmap; Network-graph; Circos plots	Python	https://github.com/forrest-lab/NATMI/	^692^
7	PyMINEr	Constructing gene co-expression networks, which are then integrated with protein–protein interaction networks	Differentially expressed genes	Protein	scRNA-seq	Likelihood of CCC for all interactions;List of gene-gene interaction networks for each cell cluster	Network visualization and Circos plots	Python and standalone application	https://www.sciencescott.com/pyminer	^697^
8	iTALK	The expression of receptors and ligands in each cell subpopulation; Only focus on the communication between tumor cells and normal cells	Differentially expressed genes	L-R	scRNA-seq	Upregulated and downregulated interactions; CCC probabilities for most significant L-R interactions	CCI networks; Circos plots; Box plots	R	https://github.com/Coolgenome/iTALK	^694^
9	CellTalker	Differentially expressed ligands and receptors in each cluster to identify unique interactions between clusters	Differentially expressed genes	L-R	scRNA-seq	Upregulated and downregulated interactions between all clusters	Circos plots of differential interactions between clusters	R	https://github.com/arc85/celltalker	^698^
10	CCCExplorer	A graph of all signaling pathways; Using ligand, receptor and downstream TF expression to identify significant interactions	Prior network, statistical inference (Fisher’s exact test) and a directed graph	L-R	scRNA-seq	Graph visualizations of all interactions	Interactive directed graphs	Standalone application	https://github.com/methodistsmab/CCCExplorer	^701^
11	NicheNet	Databases from various sources, including ligand–receptor relationships, signaling pathways and transcriptional regulatory relationships	Weighting network	L-R	scRNA-seq	Ligand interaction scores and expressing cell types for provided target pathway	Circos plots of interactions between cells or clusters	R	https://github.com/saeyslab/nichenetr	^699^
12	scMLnet	Functional intercellular communications ; intracellular gene regulatory networks	Cell-type specific gene expression, prior network information and statistical inference	L-R-TF	scRNA-seq	Tissue microenvironment-mediated inter-/intracellular signaling mechanisms of ACE2 regulation	Network diagram; Violin plots; Heatmap	R	https://github.com/SunXQlab/scMLnet	^702^
13	SoptSC	Individual cell CCC probabilities are calculated ; Integrates downstream signaling measurements into an LRI scoring function	Inferring communication networks based on cell-specific expression of ligands, receptors, and target genes	L-R	scRNA-seq	Individual cell CCC probabilities,cell cluster CCC probabilities	Circos plots of interactions between cells	MATLAB/R	https://github.com/WangShuxiong/SoptSC https://github.com/mkarikom/RSoptSC	^703^
14	Scriabin	Complexing communicative pathways ; models of downstream intracellular signaling, anchor-based dataset integratio, and gene network	Network analysis	L-R	scRNA-seq	Cell–cell pairs with different total communicative potential and finds modules of co-expressed ligand–receptor pairs	Dot plots; Bar plots; Box plots	R	https://github.com/BlishLab/scriabin	^704^
15	CytoTalk	Constructs integrated network of intercellular and intracellular gene-gene interactions based on mutual information	Prize-collecting Steiner forest algorithm	L-R	scRNA-seq	Integrated signal transduction gene network	Heatmap; Venn diagrams	MATLAB/Python/R	https://github.com/tanlabcode/CytoTalk	^705^
16	RNA-Magnet	Incorporating information on surface receptors with low mRNA expression; identifying the enrichment of signaling interactions	Network	L-R	scRNA-seq	The sum of interaction probabilities; Average interaction scores in a local neighborhood	Heatmap; Scatter plots	R	http://git.embl.de/velten/rnamagnet/	^706^
17	ContactTracing	Analysis of tumor microenvironments in mouse and patient	Network	L-R	scRNA-seq	Interactions between cells	Heatmap	Python	https://github.com/LaughneyLab/ContactTracing_Tutorial	^707^
18	scTensor	Identify key LRIs present in certain cell types; Interactions modeled using tensor decomposition,which are then scored	Tensor decomposition	L-R	scRNA-seq	HTML file with summaries of clustering, decomposition and interaction components	Many options for interaction, expression and pattern visualization	R	https://github.com/rikenbit/scTensor	^715^
19	MEBOCOST	Identifying cell–cell communications in which metabolites, are secreted by sender cells and traveled to interact with sensor proteins of receiver cells	Metabolitemediated intercellular communications	Metabolites	scRNA-seq	Communication scores, sensors and each metabolite-sensor partner to characterize the communication likelihood	Bar plots; Dot plots; Violin plots; Communication network	Python	https://github.com/zhengrongbin/MEBOCOST	^716^
20	SpaOTsc	Inferring the spatial distance between two cells; quantifying the confidence of the estimated cell–cell distance	Spatial cell–cell distance and average enrichment of genes	L-R	ST	List of inferred ligand and receptor expressions; CCC matrix for a given signaling pathway	Not Mentioned	Python	https://github.com/zcang/SpaOTsc	^737^
21	spaCI	Spatial locations and gene expression profiles of cells to identify the active L–R signaling axis across neighboring cells	Spatial relationships; Network	L-R	ST	Predicting both L–R interactions and their upstream regulators such as transcription factors	Box plots; Heatmap; Scatter plots; Network diagram; String plots; Spatial plots	Python	https://github.com/QSonggithub/spaCI	^738^
22	stLearn	Significant ligand–receptor pairs are determined on normalized gene expression which is normalized across spatial location	Expression permutation	L-R	ST	Ligand–receptor expression across discretized tissue	Gene, SCTP, Cluster and PSTS visualization	Python	https://github.com/BiomedicalMachineLearning/stlearn	^739^
23	Giotto	Generate a null distribution of LRI scores using spatial information	Expression permutation	L-R	ST	Upregulated and downregulated interactions; List of most significant ligand–receptor interactions	Heatmap; Dot plots	Python/R	https://github.com/RubD/Giotto	^740^
24	MISTy	Interactions are calculated by weighting the gene expressions of local cell neighborhood	Random forest methods; Expression permutation	L-R	ST	Network of signaling gene interactions within cell clusters and between cell clusters	Intrinsic (intraview), local niche view (juxtaview), the broader, tissue view (paraview), or others	R	https://saezlab.github.io/mistyR/	^741^
25	SVCA	Accounts for intrinsic effects, environmental effects, and cell–cell interactions	Different dimensions of spatial variation; Expression permutation	L-R	ST	Predicting genes with significant spatial variation	Violin plots	Python/R	https://github.com/damienArnol/svca	^742^
26	SpaTalk	Integrating ligand–receptor proximity and ligand–receptor–target (LRT) co-expression to model and score the LRT signaling network between spatially proximal cells	Cell-type decomposition; Spatial LRI enrichment	L-R	ST	Inferring spatially resolved cell–cell communications and downstream signal pathways	Heatmap; Sankey plot; Diagram of the LRI from senders to receivers in space; LRT signaling pathways	R	https://github.com/ZJUFanLab/SpaTalk	^743^
27	Tensor-Cell2Cell	Modeling interactions scores and optimizes Spearman correlation between distances and interaction scores; Inferring communication distance	Tensor decomposition	L-R	ST	List of enriched and depleted ligand–receptor interactions; Matrix of cell–cell interaction distances	Bar plots; Heatmap; Diagram of the LRI from senders to receivers in space; LRT signaling pathways	Python	http://lewislab.ucsd.edu/cell2cell/	^744^
28	HoloNet	Decoding FCEs by integrating LR pairs, cell-type spatial distribution and downstream gene expression	Network	L-R	ST	Generating target gene expression with the CE networks; Decoding the FCEs for specific downstream genes	Multi-view graph	Python	https://github.com/lhc17/HoloNet	^745^
29	COMMOT	It accounts for the competition between different ligand and receptor species and spatial distances, handles complex molecular interactions and spatial constraints	Collective optimal transport	L-R	ST	Inferring CCC for all ligand and receptor species; Visualizing spatial CCC at various scales; Analyzing downstream effects	Heatmap; Signaling pathways	Python	https://github.com/zcang/COMMOT	^746^
30	NeuronChat	The inference, visualization and analysis of neural-specific communication networks among pre-defined cell groups using single-cell expression data	Network	L-R	ST	A weighted directed graph composed of significant links between interacting cell groups	Circle plots; Heatmap; Chord diagram	R	https://github.com/Wei-BioMath/NeuronChat	^747^

The availability of single-cell transcriptome data and single-cell spatial transcriptome data, have led to the development of plenty of computational tools for reasoning about CCC. These tools leverage diverse methods for predicting potential intercellular communication events based on prior knowledge of ligand–receptor interactions

### Single-cell transcriptome-based tools

Single-cell transcriptome technology conducts large-scale detection of gene expression in a single cell and accurately reveals the activity of transcription factors (TFs) of each cell, which provides great support for an in-depth understanding of cell differentiation, development, and metabolism. The rapid development of single-cell transcriptomics technology, such as Drop-seq, inDrop,^682,683^ CITE-seq,^684^ 10X Genomics,^685^ providing with a deeper and comprehensive understanding in many fields of life sciences.

Several strategies have been employed to construct cellular communication networks based on LRIs using single-cell transcriptome data.^7^ Tools analyzing cellular communication based on single-cell transcriptome data are primarily limited to intercellular communication mediated by protein ligand–receptor complexes, and their analysis relies on gene expression level and ligand–receptor databases.^9^ This limitation significantly increased false-positive predictions of CCC.^686^ Absence of expression in any subunit impedes the inference of interaction between ligand and receptor and the subsequent communication.^687^ So currently developed computational approaches can be classified into four types, dependent on the mathematical frameworks for pinpointing LRIs, including (1) expression permutation-based tools, (2) difference-assembly-based tools, (3) network-based tools, and (4) tensor-based tools.^5^

#### Expression permutation-based tools

Expression permutation-based tools employ various methods to calculate communication scores for each LRP and evaluate the comparative significance to a null model using clustering label arrangement, non-parametric testing, or empirical methods. Examples include CellPhoneDB,^688^ CellChat,^686^ ICELLNET,^689^ SingleCellSignalR,^690^ CellCall,^691^ and NATMI.^692^ Notably, CellPhoneDB, CellChat, and ICELLNET consider multisubunit complexes for ligands and receptors.^5^

CellPhoneDB is at the forefront of heteromer modeling, recognizing that numerous receptors and ligands operate exclusively as heteromers. It stands out for its comprehensive cellular communication ligand–receptor database, which includes receptors and ligands annotated by public sources and specific families of hand-selected proteins involved in cell communication. However, the database does not encompass all potential LRIs and neglects other vital signaling cofactors which CellChat integrates, such as soluble agonists, antagonists, and both stimulatory and inhibitory membrane-bound co-receptors.^693^

CellChat facilitates the analysis of intercellular interactions and communication networks by providing cell interaction network diagrams and communication pathway analysis. It has expanded its coverage to include 229 signaling pathways, classified into three categories: contact between cells, receptors in the ECM, and signaling via secretion,^686^ a notable expansion from the approximate 900 LRPs featured in CellPhoneDB. CellPhoneDB infers the enriched LR interplays among the two cellular groups rooted in pronounced specificity, while CellChat emphasizes differential overexpressed ligands and receptors to measure the association between LRPs under the principle of mass action.

ICELLNET calculates an overall CCC score by summing all LRI production scores across two clusters. Meanwhile, it determines interactions through the multiplication of geometric averages from expressions of both ligands and receptors.^689^ Notably, with the exception in a singular research work,^694^ ICELLNET stands as the sole database that classifies predicted interactions into biological families.^689^ Despite ICELLNET having fewer interactions than its counterparts, it boasts precise and intricate cytokine interactions, extending timely to all chemokines and checkpoint interactions, thereby offering distinct resources for investigating intercellular communication in the immune system. For example, it contains 14 cytokine interactions not included in CellPhoneDB, for instance, MIF/CXCR2 and MIF/CXCR4.^695^

Analogous to CellPhoneDB, SingleCellSignalR introduces a notion of interaction score.^696^ that is defined as the function of the average expression of ligands in type A cells and receptors in type B cells. SingleCellSignalR relies on a new curated LR database and uses regularized expression products to deduce the underlying LRIs within cellular networks. Although the false-positive results are able to be avoided through using the permutation test that utilized in CellPhoneDB, highly representative communications in the dataset may not be statistically significant. To solve it, a definitive cut-off value for scoring score is offered in SingleCellSignalR, capable of attaining a suitable error discovery rate grounded in empirical evidence.^5^

CellCall is a toolkit that can deduce both intercellular and intracellular communication routes by amalgamating coupled LRIs and TF activity. Distinguished from scoring method of cellular interactions in SingleCellSignalR and CellPhoneDB, the algorithm of CellCall uses the expression information of the RegB regulon which are target genes activated by the co-expressed TF.^696^ Furthermore, CellCall utilizes an integrated pathway activity analysis technique to pinpoint notably active pathways in intercellular dialog among distinct cell types. However, CellCall focuses exclusively on the downstream gene regulatory networks (GRNs) related to LRIs. It primarily focuses on LRPs comprising protein-based partners, thereby neglecting non-peptidic entities like lipids, small molecules, nucleic acid ligands, and carbohydrates.^691^ In short, CellCall and SingleCellSignalR can detect a large number of communications, including nonspecific communications, but may miss low-intensity communications.^690^

NATMI can be used to summarize the complete network of communication to display the communication intensity or specificity between each cell type and other cell type in complex samples, so as to identify highly communicative cell pairs or specific communities.^692^ It uses connectomeDB2020 or user-defined LRPs to forecast and visualize cell communication network between cell types in datasets.

These expression permutation-based tools, as mentioned above, typically solve the limitation that most CCC tools do not consider multisubunit protein complexes.

#### Difference-assembly-based tools

Several difference-assembly-based tools have been developed, including PyMINEr,^697^ iTALK,^694^ and CellTalker.^698^ PyMINEr and iTALK aimed to identify the differential expression genes between cellular clusters and used them as candidates for the final LR pair interactions. PyMINEr establishes gene co-expression networks ascertained through Spearman correlation and integrates them with protein–protein interaction networks.^697^ Unlike SingleCellSignalR, which depends on genetic signatures for conducting cell-type identification,^690^ PyMINEr’s characteristic gene signature is not provided. Instead, its approach to delineating cell types hinges upon the enrichment of subgroup-specific gene pathways. Actually, PyMINEr identifies altered signaling pathways based on differentially expressed pairs of ligands and receptors. But iTALK categorizes LRPs into cytokines, growth factors, immune checkpoints, and others, focusing solely on communication between tumor cells and normal cells.^694^ In contrast, CellTalker identifies unique interactions between clusters by using differentially expressed ligands and receptors within individual clusters.^698^ It assumes cellular communication hinges on the uniform expression of ligands and receptors among interacting cells. In short, CellTalker and iTALK employ slightly different downstream analysis techniques to assemble the definitive roster of pivotal interacting LRPs. However, these methods may overlook common interactions among all groups though they excel at identifying LRIs within the dataset’s background.

#### Network-based analysis tools

The network approach is utilized by several tools, leveraging gene connectivity properties. Intercellular communication encompasses intercellular signaling, intracellular transmission, and signal amplification through specific signaling pathways. These pathways often result in activity changes of downstream TF and GRNs.^699,700^ Various approaches have considered intracellular signaling to tackle these complexities, including CCCExplorer,^701^ NicheNet,^699^ scMLnet,^702^ SoptSC,^703^ Scriabin,^704^ CytoTalk,^705^ RNA-Magnet,^706^ and ContactTracing.^707^

CCCExplorer builds a comprehensive graph depicting various signaling pathways and computes a statistic akin to Fisher’s method by employing the expression of ligands, receptors, and downstream TFs to pinpoint key interactions.^701^ It incorporates differentially expressed genes and PPI networks to analyze downstream targets and TFs to determine signal events of cell activation or inactivation. Because a functional understanding of CCC requires knowledge about the effect of ligand on receptor’s gene expression, the expression data of interacting cells is needed to infer the effect of sender-cell ligands on the expression of receptor cell.

To address this problem, a computational method called NicheNet has been developed by integrating data from various sources, including ligand–receptor relationships, signaling pathways, and transcriptional regulatory relationships. It can directly output the inter-relationships among ligands, receptors, and target genes.^699^ Since the prior model of ligand-target regulation potential mainly relies on prior network information instead of expression relationships in specific cells, the construction of context-dependent multilayer, intercellular and intracellular signaling networks is needed to deeply understand CCC through single-cell gene expressions functionally.^702^

Thus, another tool named scMLnet has been developed using specific type of cell gene expression, prior network information, and statistical inference. This approach can not only model communications and GRNs among cells, but also infer how intracellular gene expression is affected by the cellular interactions.^708^

Different from most methods which have tried to predict CCC between various cellular clusters, SoptSC enables to decipher the interactions between individual cells.^703^ In SoptSC, individual cell CCC probabilities are calculated using nonlinear functions involving the products of ligand and receptor expressions, where target gene responses can be weighed. However, it could not automatically detect disconnected lineages and infer bidirectional arrows for certain cell state transitions.^703^

Similar to SoptSC, Scriabin is a flexible and computationally effective approach for analyzing communication pathways using single-cell level information.^704^ It utilizes comprehensive databases of curated LRIs,^688,700,709^ intracellular signaling and anchor points to analyze gene networks.^710^ It should be noted that this method assumes the consistent credibility of LRPs within expertly curated protein–protein interaction repositories. Downstream signaling analyses in Scriabin are dependent on NicheNet’s matrix of ligand-target activities, potentially influenced by the specific cell types and stimulation conditions employed in its creation. In addition, NicheNet’s database lacks capabilities for analyzing inhibitory signaling, leading Scriabin to primarily return CCC edges that are anticipated to activate signals.^704^

In addition, CytoTalk initially builds a comprehensive network containing both intracellular and intercellular communications. Compared with NicheNet and SoptSC, the differential expression of downstream pathway genes is more significant from CytoTalk prediction. Unlike the previous methods using known pathway annotations,^699,703^ CytoTalk is able to construct signal transduction pathways from scratch and compare them in different tissues or conditions, representing a significant improvement over existing algorithms.^705^

Moreover, RNA-Magnet utilizes fuzzy logic for the identification of active ligands and receptors in cellular communication.^5^ This method forecasts potential physical interactions among individual cells and chosen attractor groups by integrating the expression patterns of cell surface receptors with their corresponding surface-expressed mRNA.^688,711,712^ RNA-Magnet assigns scores to indicate the degree of attraction for each cell, along with a direction showing the attractor group to which the cell is primarily drawn. It has been reported that the RNA-Magnet algorithm can accurately infer the three-dimensional organization of bone marrow from the expression data of single-cell genes.^713^ However, RNA-Magnet may have limitations with only heterodimer receptor information for integrins in early version and the installation process may involve multiple dependencies which may be challenging for some users.^706^

Furthermore, ContactTracing represents an innovative systemic method to forecast the impact of condition-dependent cellular interactions within TME.^707^ This method analyzes TME along with varying levels of chromosomal instability by utilizing the inherent variability of scRNA-seq data to infer cell responses to ligand–receptor-mediated interactions, independent of previously existing downstream target gene knowledge.^707^

The advantage of these network-based methods lies in their utilization of ligand and receptor expression levels to calculate interaction score and altered expression of downstream signaling targets. However, such approaches have limitations in addressing signal crosstalk, which may result in the occurrence of false-positive or negative outcomes, especially in cases where intracellular pathways are modulated through posttranslational modifications instead of transcriptional regulation, as observed in certain cytokine signaling pathways.^714^

#### Tensor-based analysis tools

Tools based on tensor analysis constitute the group with the highest mathematical complexity. For instance, scTensor is an innovative approach for deriving representative triadic relationships, encompassing ligand expression, receptor expression, and associated LRPs. One of its attractive features is that LR reference is available for many organisms. This approach utilizes Tucker decomposition on a third-order tensor to pinpoint key ligand–receptor interactions (LRIs) that are specific to particular cell types.^715^ The scTensor utilizes a potential LRP database automatically generated by interactions from STRING and annotations from Swissprot (secreted/membrance), and revealed a significant quantity of presumed LR pairs. The CCC network is constructed as a directed hypergraph with multiple edge types representing different LRPs. Tensor decomposition is used to model these interactions and calculate their scores.^715^ Although these tools can capture communication pathways involving all cell pairs simultaneously and extract relationships between different CEs, interpreting fractions from tensor decomposition might not be as straightforward as other tools.

#### Other principles and strategies

Unlike tools that use LRPs as mediators, MEBOCOST is an algorithm based on computational methodology designed to infer the dynamics of metabolite-driven intercellular communications quantitatively using scRNA-Seq data.^716^ By considering the expression of enzyme production, data regarding the secretion of metabolites has been incorporated in the transmission study, and it’s possible to deduce the synthesis of particular metabolites from transcriptome data.^717–719^ This algorithm detects interactions between cells where sender cells secrete metabolites like lipids, which then engage with the sensor proteins in receiver cells. MEBOCOST identifies cells emitting and receiving an extracellular metabolite, contingent on their respective enzyme and sensor expression levels, thereby identifying communications between cells involving metabolite sensors. The MEBOCOST algorithm accounts for both the synthesis and utilization reactions of metabolites. Its design ensures compatibility with established algorithms that ascertain the existence of single-cell metabolite via flux balance analysis, such as scFEA and COMPASS. Nevertheless, the frequently nonlinear correlation between metabolite quantities and RNA contents of metabolic enzymes poses a challenge that this algorithm cannot provide quantitative calculations of metabolite abundance.^716^

While high-throughput scRNA-seq methods describe cell populations heterogeneity,^720^ they lack the ability to offer phenotypic information, such as cell surface protein levels.^684^ Meanwhile, the targeted method for measuring expressed proteins in a single cell is limited in scale and limited profiling method is achievable for detecting a plentiful of genes and proteins in parallel.^721,722^ Fluorescent-labeled antibodies targeting cell surface proteins serve as reliable indicators of cellular activity and function.^723^ The method known as CITE-seq using oligonucleotide-labeled antibodies through sequencing addresses this limitation by utilizing sequencing-based strategy that simultaneously quantifying transcriptome and cell surface protein in single-cell level. CITE-seq.^684^ not only describes cellular transcriptomes and epitopes indexing but also is compatible with existing single-cell analysis approaches. Compared to separate transcriptome measurements, multimodal data analysis through CITE-seq provides detailed cellular phenotype features.

It is noteworthy that since CCC serve as the downstream of all data analyses, setting thresholds for LRIs can impact the interpretation and explanatory power of CCC results. A lower threshold may lead to the identification of more interactions, including those with lower expression levels or frequencies, aiding in the discovery of potential novel LRPs. However, this may also introduce noise, including false-positive results.^5,9^ Conversely, a higher threshold may reduce false positives but could also result in missing some true LRIs. Therefore, when setting the threshold for LRIs, a balance needs to be struck based on the specific research objectives and characteristics of the data used to achieve the most accurate and interpretable CCC results.

### Spatial transcriptome-based tools

Typically, cellular interactions are confined to restricted areas, which is not captured by scRNA-seq.^5^ In order to minimize incorrect exclusion in CCC analysis, integrating the cell’s medium spatial position is essential.^724^ Spatial transcriptome technology enables transcriptome profiling from cells in different locations on tissue sections, facilitating the analysis of gene expression characteristics at diverse spatial positions within tissues. mRNA serves as the functional copy of active genes, and their localization within living tissues is often related to the regulation of cell and tissue growth and development. Previously, the analysis of multiple mRNAs simultaneously required the crushing of cells, making it impossible to understand the localization of mRNA within cells. Fluorescent in situ RNA sequencing (FISSEQ) can reveal environmentally specific transcripts while preserving the tissue architecture necessary for RNA localization.^725^ This technique is applicable to tissue sections and whole embryos, is not overly limited by optical resolution, and can reduce noise signals in single-molecule detection.^726^ In addition, it enables large-scale parallel detection of genetic elements, assisting researchers in analyzing cell phenotypes, gene regulation, and in situ environments. Currently, the 10X Genomics Visium.^727,728^ technology stands as a mainstream commercial spatial transcription technology; however, its detection resolution remains below the true single-cell level. Conversely, the 10X Genomics Xenium.^729^ technology significantly enhances spatial resolution by in situ fluorescent imaging, capturing RNA expressions at single-cell or subcellular levels. This technology swiftly detects the in situ expression level of numerous targets on fresh frozen (FF) or formalin-fixed paraffin-embedding (FFPE) tissue sections. By employing existing or customized probe panels and targets, this method achieves subcellular resolution, offering insights into cell structure and function. As another image-based spatial approach, MERFISH facilitates the detection and measurement of a multitude of RNA types, ranging from hundreds to thousands, at the single-cell level.^730^ It employs specific fluorescent labeling strategies to simultaneously detect multiple RNA molecules. Notably, MERFISH demonstrates fault-tolerant capabilities, accurately identifying RNA species despite minor fluorescent labeling errors. Xiaowei Zhuang’s team at Harvard University successfully employed MERFISH technology to recognize over 100 neuronal and non-neuronal cell populations in the human brain with high-resolution images.^731^ Currently, fault-tolerant fluorescence in situ hybridization techniques like MERFISH^730^ and seqFISH+^732^ are primary hybridization-based in situ transcriptomic methods. ST technologies can create “atlases” with spatial information, revealing which cells constitute each tissue and how they are organized and communicate.^733^ However, the imbalance between resolution, gene capture, and field of view in current methods hinders the construction of atlases with “higher spatial resolution” and “broader transcriptome coverage”.^734^ The Stereo-seq technique actively addresses these challenges, and is capable of analyzing genes and imaging simultaneously.^735,736^ This technology allows for ultra-high precision analysis of gene and cell changes over time and space during the developmental processes of life, achieving a comprehensive spatiotemporal molecular atlas of life.

So the spatial transcriptome is crucial in locating and distinguishing the active gene function expressed in distinct tissue areas, offering key insights for diagnostic and therapeutic purposes. Creating tools for single-cell ST analysis to clarify regulatory processes controlling cell state changes hold great significance for research in fields such as cancer pathogenesis, neuroscience, developmental biology, and others. Therefore, many tools such as SpaOTsc,^737^ spaCI,^738^ stLearn,^739^ Giotto,^740^ MISTy,^741^ SVCA,^742^ SpaTalk,^743^ Tensor-Cell2Cell,^744^ HoloNet,^745^ COMMOT,^746^ and NeuronChat^747^ have been developed.

Non-spatial single-cell methods frequently yield considerable false positives, as CCC occurs within confined spatial ranges unmeasured in such datasets. Thus, SpaOTsc was developed to infer the spatial distance of two cells by contrasting their predicted spatial distributions, then provide a useful linkage between them and quantify the reliability of the estimated distance.^737^ As a network approach, optimal transportation is used in SpaOTsc to model intercellular communication. However, computational challenges arise as datasets expand beyond manageable sizes. In addition, this approach does not account for potential time delays in CCC. Owing to frequent signal dropouts and noise signals in single-cell ST data, another network-based tool spaCI has been proposed using an adaptive graph model with attention-based mechanisms. It combines the neighboring cells’ spatial position and expression profiles to determine the active LR signaling axis. More importantly, spaCI allows detection of upstream TFs that mediates the LR signaling axis, and enhances comprehension of the potential molecular mechanism of intercellular crosstalk which network-based methods in single-cell transcriptome are blind to.

Methods such as SpaOTsc and spaCI have not combined spatial cell-type distribution and LR interaction to find hotspots that may have high CCC activities. So, a tool based on expression permutation, called stLearn, was developed to automatically scan areas with high cell-type densities and co-expressed LRPs, suggesting a highly interactive area.^732,748,749^ Similarly, Giotto, MISTy and SVCA can infer the interaction in the local cell niche by establishing the statistical significance of the automatically recognized cell-type distribution in neighborhood.^62,740,749–752^ Giotto incorporates spatial expression information with the possibility of cell interactions by creating a null distribution of LRI scores to recognize key interactions. It analyzes and isolates interactions between nearby cell clusters based on the construction of spatial networks from spatial transcriptomics. In contrast, MISTy is an explainable framework for analyzing highly multiplexed spatial data without requiring cell-type annotation. This method identifies crucial marker genes in particular regions through random forest algorithms and calculate interactions by applying weights to gene expressions in local cellular environments. Similar to Giotto and MISTy, another computational framework SVCA was developed to quantify spatial variation in different dimensions by analyzing the interactions between markers within different spatial contexts.

However, Giotto and SpaOTsc are limited to inferring CCC between single-cell ST data rather than the spot-based ST data and between paired cell types rather than paired cells.^743^ It still lacks methods capable of inferring and visualizing spatially resolved CCC at single-cell resolution through ST data. The emergence of SpaTalk enables statistical analysis and visualization of spatially proximal LRIs, forming a dynamic CCC network.^753^ By incorporating spatial information, SpaTalk displays enriched LRIs among spatially proximal co-expressed cell pairs at single-cell resolution, providing an informative method for analyzing and visualizing LRIs and their mediated CCC from different perspectives.^743^ This offers a powerful tool for resolving key CCCs in normal physiology and pathological processes at spatial single-cell resolution. In addition, Tensor-Cell2Cell is an unsupervised approach based on tensor decomposition and unravels context-specific CCC by analyzing various cell stages, states, or locations concurrently.^744^ In short, these methods facilitates the integration of spatial location, structural characteristics, and expression patterns to address significant biological questions including cell-type identification and intercellular communications.^739^

Although SVCA and Tensor-Cell2Cell was built to characterize the dependencies of sender-receiver cell as well as the related phenotypes, a method for systematically decoding functional CEs was still lacking. Considering only functional but not irrelevant CEs involving specific biological processes can help to better understand the role of intercellular communication in shaping certain cell phenotypes and formulate possible disease interventions.^745^ Then HoloNet was developed to characterize communication landscape and identify both cell types serving as main sender and LRPs serving as core mediators of the specific downstream gene in functional CEs.^745^

Multiple ligands can bind to multiple receptors, thereby generating competition, a ubiquitous and crucial biophysical process among multiple molecular species.^746^ However, current methods examine CCC on local and independent cell pairs, focusing on information between cells or near individual cells. Thus, collective or global information in CCC, such as competition between cells, is overlooked. To address this issue, COMMOT was developed by taking into account the competition between different ligand and receptor species as well as the spatial distance between cells.^746^ Besides, brain function depends on signal transmission between a vast number of neurons and non-neuronal cells. The connectome—the connective tissue of neural connections—is subject to transcriptional regulation.^754,755^ Emerging spatial transcriptomics methods,^66,732^ besides measuring gene expression within cells, also measure the spatial location of neuronal cells, providing a rich resource for dissecting neuronal heterogeneity. However, these methods are not suitable for characterizing communication between neurons, as neurons can extend axons and dendrites over long distances to form synapses and primarily communicate through neurotransmitter signals.^756–758^ The development of NeuronChat took into account neurotransmitter signaling and system-level neuron-specific cell-to-cell communication networks, incorporating the process of neural signal transmission to infer intercellular communication. This makes NeuronChat distinct from existing methods for inferring intercellular communication that do not account for neuronal activity.^747^

However, the limited ability of in situ hybridization technology and the applicability of NGS solely to homogenized tissues fail to fully capture the complexity of a human TME.^759^ To overcome this challenge, GeoMx DSP spatial multi-omics technology^71^ and CosMx SMI single-cell space in situ imaging technology.^760^ are developed for spatial analysis of multiple targets, which realize the direct evaluation of complete tissue microenvironment and local drug effects in situ of patients’ tumor tissues.^761^ Furthermore, these methods capable of simultaneously detecting mRNA and protein by binding oligomer antibodies,^762^ offer a comprehensive view of the full transcriptome, successfully applied in spatial gene expression studies across various organs and tissues.^763^

### Single-cell proteomics and spatial proteomics

Focusing on understanding the qualitative and quantitative aspects of protein composition within single cells, proteomic analysis at the single-cell level and spatial proteomic studies are emerging. This analysis unveils differences in the proteome between individual cells, providing a detailed molecular map of proteins. This information aids in comprehending cellular variations in phenotype and function. In the realm of cell communication, single-cell proteomics offers in-depth insights into how cells interact through specific proteins and signaling pathways. These interactions involve the “secret language” composed of signals like cytokines and membrane proteins, which connect cells to ensure the efficient functioning of life, a core aspect of cell communication research.

Single-cell proteomics technologies, such as mass cytometry, enable the simultaneous analysis of 50 parameters at the single-cell level, encompassing proteins, nucleic acids, and small molecules, all achieved with a high signal-to-noise ratio. CyTOF,^764^ which stands for Cytometry by Time of Flight, employs mass cytometry to quantify labeled targets on both the surface and interior of individual cells. This technology enables the simultaneous quantification of multiple cellular components by employing a detector based on inductively coupled plasma MS (ICP-MS). The principal benefit of CyTOF lies in its capacity to leverage immunolabeling to quantify proteins, carbohydrates, or lipids within a cell. This innovative technology has revolutionized discovery and clinical research by allowing researchers to simultaneously interrogate over 50 markers on millions of individual cells. Furthermore, CyTOF combines MS and flow cytometry principles, enabling single-cell protein expression analysis, which is crucial for advancing studies in both steady-state and pathological processes. Most importantly, MS flow cytometry allows high-throughput and high-resolution detection of multiple parameters in a single cell, making it invaluable for studying CCC. This technology can not only detect receptors and ligands on the cell surface but also identify signaling molecules within the cell. One significant advantage is that it’s not constrained by overlapping fluorescence spectra, allowing the simultaneous detection of more parameters. This comprehensive information provides deeper insights into the mechanisms underlying cell communication.

Spatial proteomics explores the spatial distribution and function of proteins within cells, considering that eukaryotic cells are highly compartmentalized, and different biological processes occur in distinct cellular compartments. A protein’s function depends closely on its sub-localization within the cell, as different compartments provide varying chemical environments, such as pH and redox conditions. Proteins are functional molecules of all cellular functions and processes. Thus, the spatial expression of proteins is essential for determining their precise locations and roles in tissues. Proteins can change depending on cell-type, cycle stage, disease state, and treatment methods. Consequently, spatial proteomics serves as an effective approach for examining alterations in spatial expression pattern of proteins associated with diseases, offering new perspectives for biomarker discovery and therapeutic development. Recently, the field of spatial proteomics has achieved significant advancements in the aspects of microenvironment and disease development, mechanisms and drug targets, organ structural heterogeneity, and tissue or organ spatial mapping.

Traditional proteomic techniques primarily focus on detecting protein expression levels in cell or tissue lysates, lacking essential spatial location information. Spatial proteomics technologies have emerged to address the limitation. Nevertheless, these technologies form the groundwork for two essential spatial imaging techniques, imaging MS cytography (IMC) and multiple ion beam imaging (MIBI).^763^ IMC integrating immunocytochemistry and immunohistochemistry techniques with high-resolution laser ablation into CyTOF MS flow cytometry.^765^ It complements existing imaging methods, delineates cell subgroups and intercellular interactions, and accentuates tumor heterogeneity. Similarly, MIBI employs secondary ion MS for imaging antibodies tagged with metal isotopes and analyzes samples marked with as many as 100 different metal isotope-labeled antibodies.^766^ This technique is compatible with conventional FFPE tissue slices, a prevalent specimen type in clinical repositories worldwide.^767^ The emerging MS.^768^ technology for identifying and quantifying proteins can not only measure the abundance of proteins and PTMs in individual cells, but also measure their complexes and subcellular localization.

However, these methods based on ion MS encounter limitations concerning the availability of sufficient pure metals. On the other hand, traditional fluorescence immunohistochemistry technology faces limitations in achieving single-cell analysis due to optical constraints and difficulty in imaging more than seven biomarkers in a sample. In contrast, Cell DIVE circumvents this hurdle through multiple rounds of staining, enabling the imaging detection of 60 biomarkers in a single sample by direct labeling with fluorescent dyes.^69^ Similarly, another single-cell proteomic analysis platform CODEX enables to offer intricate details regarding protein distribution in 2D space.^62,769^ The fundamental design principle underlying CODEX involves marking specific oligonucleotide “Barcode” on individual antibodies,^770–772^ instead of direct labeling with fluorescent dyes used in Cell DIVE.^69^ The fluorescent dye necessary for imaging selectively binds to the complementary oligonucleotide sequence of the “Barcode”. In summary, these innovative approaches allow us to surpass limitations associated with the number of visible spectral fluorescence imaging channels, facilitating the simultaneous detection and analysis of 50 or more protein indicators. Meanwhile, pathological analysis software facilitates the semi-quantitative analysis of diverse biomarker molecules in each cell.

### Single-cell multiomic tools

The continuous advancement of single-cell omics technology has equipped us with a potent tool to explore cell interaction and communication. Techniques including single-cell transcriptomics and proteomics, offer detailed observations of gene expression and protein synthesis in single cells, respectively. These technologies have broad applications in biological and medical research, offering fresh insights and possibilities for deepening our understanding of cell interaction and communication.

Single-cell sequencing is an advanced biotechnology that facilitates detailed decoding of gene expression and genetic alteration for each cell in a tissue sample. For example, single-cell DNA sequencing permits accurate DNA mapping for each unique cell. Going beyond this, the more intricate single-cell multi-omics sequencing, this is to say single-cell multimodal omics analysis, facilitates the simultaneous acquisition of various dimensions of omics data from an individual cell. Practically, single-cell multi-omics analysis efficiently records different features of the identical cell in multiple tissue samples, encompassing DNA, RNA, epigenetic regulations, and protein patterns. This powerful approach has found extensive application in systematically unraveling the intricate interaction mechanisms of critical components and pathways within cells, contributing significantly to our understanding of complex cellular processes.^773^

Single-cell multi-omics research integrates diverse techniques from various disciplines to scrutinize the variability among different cells. Simultaneously, this integration enables a comprehensive and quantitative analysis of the multi-dimensional data associated with distinct cells, exploring their potential biological significance. Nonetheless, it confronts challenges such as intricate technical complexities, substantial data volumes, and multiple dimensions of data, necessitating ongoing development and optimization of analytical methods and research models in the realm of multi-omics technology.

Besides, the single-cell transcriptome offers the capacity for large-scale simultaneous analysis of thousands of molecular attributes within a single cell, uncovering pivotal genes associated with distinct cell types and highlighting disparities among various cell categories. However, the underlying mechanisms governing these differential expressions remain largely unclear. In this context, single-cell multi-omics emerges as a potent tool capable of elucidating the internal interplay between gene expression and epigenetic regulation within the same cell. It provides a means to establish the direct connections between candidate regulatory elements and their target genes, allowing for the definition of regulatory elements and cell states specific to unique cell types. This approach aids in elucidating the root causes of gene expression disparities and unveils the regulatory network underpinning genes linked to tumors and diseases, along with the mechanisms governing them.

Finally, considerable strides have been taken in refining methods and applying single-cell and spatial multi-omics technologies. Some tools have incorporated multi-omics information in inferring CCC, precisely mapping single-cell data into spatial transcriptomics data. For instance, the updated version of CellPhoneDB v5 significantly improves the database and computational methods to infer, prioritize, and visualize CCC, utilizing other single-cell modalities such as spatial information or TF activity.^774^ Apart from this, CellChat v2 now enables the inference of CCC from multiple spatially resolved transcriptomics datasets as well. By optimizing the CCC algorithm through the integration of multi-omics data, the accuracy of cell-type classification can be improved, thereby enhancing the calculation results of cell proportions and log2 values. These techniques empower the exploration of the molecular hierarchy, bridging the gap from the genome to the phenotype within a single cell. They offer insights into the dynamic interplay between gene regulation from epigenome and gene expression from transcriptome or proteome across various biological processes, including development, aging, and diseases. In addition, these technologies facilitate the study of the impact of genetic variations acquired by individual cells on their unique functional and phenotypic characteristics, along with their influence on surrounding tissue functions and other factors.^775^ With ongoing advancements in single-cell technology, we can look forward to deepening our understanding of the intricate network of interactions between cells. In turn, this offers novel strategies and pathways for disease prevention and treatment. Since the method for inferring CCC possesses unique advantages and constraints, the utilization of these methodologies necessitates awareness of their strengths and weaknesses, and the careful choice of analytical parameters.^5^ While methodological and technical challenges persist, there are abundant opportunities for enhancing our comprehension of cellular interactions. Looking ahead, we can anticipate further breakthroughs in the realms of biology and medicine through continued advancements in single-cell research.

Investigating mechanisms underlying CCC remains a prominent area of exploration in physiology and the broader life sciences. Although we have made remarkable progress, there are still many challenges in understanding single-cell communication and interaction, which require further research and exploration to better solve current problems in biomedical research. Research has revealed that the intricacies of signal transduction pathways are exceedingly complex, involving protein interactions and the expression processes of associated genes. However, complex interconnections exist among various signal transduction pathways, forming an intricate dialog between signals and even an entire signal network system. Despite significant progress in recent years, facilitated by various “omics” approaches, exploring signal transduction mechanisms requires further in-depth research and examination. Therefore, developing cutting-edge tools for deciphering cellular interactions and their integration with multi-omics approaches is pivotal for advancing the treatment of diverse diseases and the progress of the medical field.

## Challenges and perspectives

CCC is a fundamental mechanism for multicellular organisms to adapt to internal and external environmental fluctuations, and to preserve homeostasis. Through CCC, biochemical and physical signals are dispatched and received between cells, influencing cell phenotypes and functions.^704^ However, the present research challenge is that current approaches for deciphering intercellular communication from scRNA-seq data predominantly analyze at the classification of cell subtypes or cluster level, often overlooking information in individual cells.

The challenges include:

1. Data parsing complexity: While scRNA-seq technology offers the capability to dissect intricate multicellular niches at single-cell resolution, it is essential to recognize that CCC does not operate at a population level but transpires at single-cell scale. Hence, the development of novel CCC inference methods is imperative. These methods should examine single-cell dynamics and their interplays, and capitalize on the full spectrum of information encapsulated within scRNA-seq data.^704^
2. Complex experimental design: Traditional approaches to investigating intercellular interactions often involve expensive equipment and intricate procedures. Moreover, these methods exhibit limited flexibility and are often incompatible with other analytical processes.^776^

Besides this, the current applicability of CCC is more widespread in the cell–cell interactions under physiological conditions. However, during pathological or post-treatment processes, cell-type transitions occur, impacting the accuracy of analysis. To improve accuracy, the following methods can be considered:

1. Use of multi-omics approaches: Combining single-cell transcriptomics, proteomics, and metabolomics can comprehensively analyze changes in cell–cell interactions, reducing analysis biases caused by cell-type transitions.
2. Development of precise cell markers: Developing new cell markers that can more accurately distinguish different cell types and monitor changes in cell states more finely.
3. Conducting validation experiments: Based on the analysis results, perform validation experiments to confirm the impact of cell-type transitions on the results and further validate the accuracy of the analysis.
4. Integration with clinical data: Integrating experimental data with clinical data for analysis can better understand the role of cell-type transitions in disease development and treatment processes, improving the accuracy and credibility of the analysis.

As OMICS technologies undergo rapid advancement, research into cellular communication networks has also made substantial progress. This research unveils the foundational structure and functions of cell communication networks and lays the experimental groundwork for application in various related fields.

In the future, research into cell communication networks is poised to attain greater depth, with specific prospects including: (1) Research based on multi-omics cell communication network. (2) Exploration of the dynamic changes in the structure and functionality of cell communication networks. (3) In-depth investigation of pivotal components and interactions within signaling pathways. (4) Research focused on drug-targeted therapy and the prediction of side effects. (5) Utilization of microfluidic systems has emerged as practical tools for researching cell–cell and cell-ECM communications. Microfluidic systems offer advantages such as low reagent consumption, precise management of reagents, high throughput, and seamless integration of functional components. They facilitate comprehensive studies of cellular interactions at population and single-cell levels.^776^ (6) The development of novel multi-omics analysis methods for inferring CCC.

In summary, the structural analysis and application of CCC networks have huge significance, and its research holds substantial potential for addressing a multitude of challenges in life sciences.

## Supplementary information

Supplementary Table 1

Supplementary Table 2
